# 5-Substituted Flavones—Another Class of Potent Triplex DNA-Specific Ligands as Antigene Enhancers

**DOI:** 10.3390/molecules29245862

**Published:** 2024-12-12

**Authors:** Landy Gu, Nghia Tran, Vanessa M. Rangel, Mandeep Singh, Krege M. Christison, Geoff P. Lin-Cereghino, Liang Xue

**Affiliations:** 1 Department of Chemistry, University of the Pacific, 3601 Pacific Avenue, Stockton, CA 95211, USA; l_gu2@u.pacific.edu (L.G.); n_tran44@u.pacific.edu (N.T.); vanessa.rangel@deltacollege.edu (V.M.R.); m_singh11@u.pacific.edu (M.S.); k_christison@u.pacific.edu (K.M.C.); 2 Department of Biological Sciences, University of the Pacific, 3601 Pacific Avenue, Stockton, CA 95211, USA; glincere@pacific.edu

**Keywords:** triplex-specific ligands, flavones, microcalorimetry, ligand-mediated triplex formation

## Abstract

In the field of drug development, the quest for novel compounds that bind to DNA with high affinity and specificity never ends. In the present work, we report the newest development in this field, namely, triplex DNA-specific binding ligands based on the 5-substituted flavone scaffold in our lab. Biophysical studies showed that the newly synthesized flavone derivatives (depending on the side chains) bind to triplex DNA with binding affinities better than or similar to 5-substituted 3,3′,4′,7-tetramethoxyflavonoids. These compounds selectively stabilize triplex DNA while having little effect on duplex DNA, as verified by various biophysical methods. A detailed structural analysis suggested that the binding of these compounds to triplex DNA depends on the type of amino groups in the side chains and the length of the side chains. Viscosity studies suggested that these ligands bind to triplex DNA via intercalation. A representative ligand, compound **4b**, showed a positive inhibitory effect on the activity of a restriction endonuclease (*DraI*) via ligand-mediated triplex formation. Several of these compounds exhibited excellent cytotoxicity toward various cancer cell lines (HT-29, HCT116, and HL-60), as indicated by the MTT assay. The work presented here is part of a continued effort from our laboratory to explore the novel structural motifs of natural product flavonoids for the development of triplex-specific ligands as antigene enhancers.

## 1. Introduction

**Figure 1:**
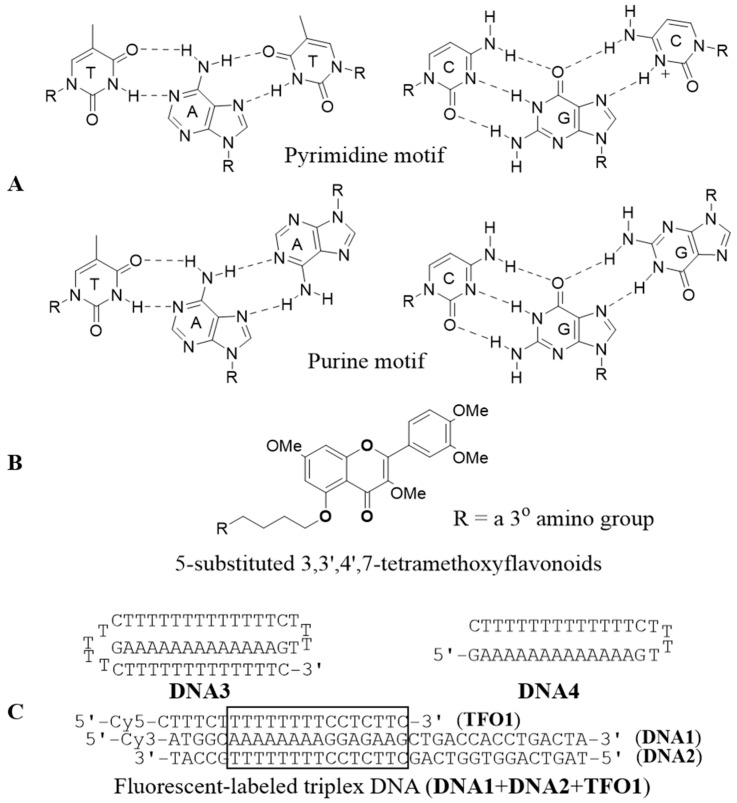
(**A**) Structures of base triplets for pyrimidine motif (T•AT and C^+^•GC) and purine motif (TA•A and CG•G). (**B**) The general structure of 5-substituted 3,3′,4′,7-tetramethoxyflavonoids. (**C**) The intramolecular DNA structures of **DNA3** (triplex) and **DNA4** (duplex) used in the ITC experiments, and the triplex structure formed by **DNA1**, **DNA2**, and **TFO1** used in the gel shift assay. The triplex-forming region is outlined with a box.

The sequence-specific targeting of duplex DNA has always been a focus of interest in drug development as it allows researchers to regulate DNA replication and transcription with reduced off-target effects. DNA sequences with at least 15–16 nucleotides in the human genome are considered distinct for selective recognition. Such recognition can be achieved using the antigene strategy, in which a short triplex-forming oligonucleotide (TFO) binds to duplex DNA in the major groove to form a triple helical structure in a sequence-specific manner [1]. In general, the formation of triplex DNA is categorized into either a pyrimidine or purine motif (Figure 1A) [2,3,4]. The former involves the binding of a pyrimidine TFO to homopurine–homopyrimidine duplex DNA via Hoogsteen hydrogen bonds. The pyrimidine TFO has the same strand polarity as the homopurine strand. The latter involves the binding of a purine TFO to homopurine–homopyrimidine duplex DNA via reverse Hoogsteen hydrogen bonds. The purine TFO strand has an opposite strand polarity to the homopurine strand. TFOs are essentially major groove ligands that can block the binding of nucleic acid processing proteins to the same DNA region (the antigene strategy) and subsequently interfere with biological activities, such as gene expression regulation [5,6,7] and DNA repair and recombination [8,9], inducing genetic instability [10]. Hence, the formation of triplexes has been highlighted as a useful tool in molecular assembly [11,12], molecular biology [13], diagnostics [14], and therapeutics [15]. However, triple helices are inherently unstable due to the repulsion between three negatively charged strands, making them dissociate at much lower temperatures than their duplex counterparts. Triplex formation is thermodynamically and kinetically unfavorable compared to duplex formation [16], limiting its biological applications. Antigene enhancers are a class of small synthetic or natural molecules that facilitate the binding of a TFO to duplex DNA and subsequently enhance triplex stability [17,18]. Amongst the triplex ligands reported, many exhibit poor selectivity for triplex over duplex DNA [19,20,21,22]. Hélène and his co-workers rationally designed a triplex-specific ligand, BQQ, by matching its aromatic surface area with base triplets to maximize π–π interactions upon intercalation [23]. Arya’s group reported the first triplex-specific groove binder, neomycin, from a series of aminoglycosides [24]. Neomycin binds into the Watson–Hoogsteen groove of triplex DNA while having minimal binding with duplex DNA. The high selectivity results from the unique spatial arrangement of the amino groups in the oligosaccharide structure.

**Scheme 1:**
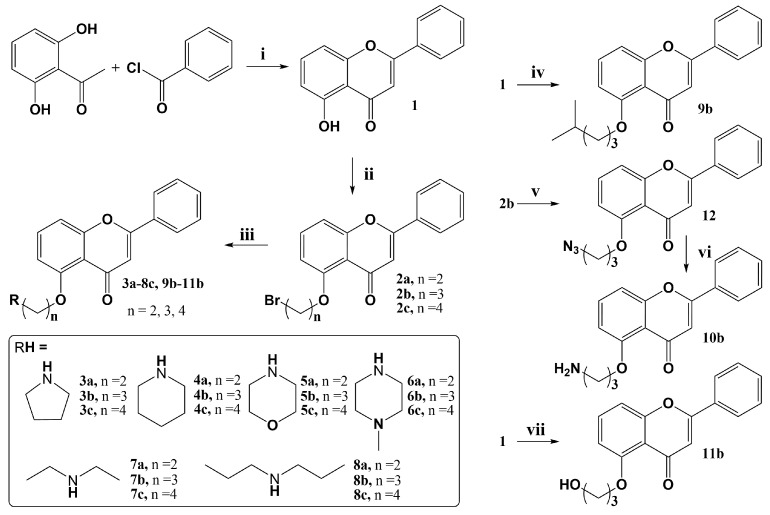
Synthesis of compounds **1**−**8c**, **9b**, **10b**, **11b**, and **12**. (i) K_2_CO_3_, acetone, reflux, 24 h, 40%; (ii) Br(CH_2_)_n_Br, DMF, K_2_CO_3_, r.t., 3 d, 25–53%; (iii) DMF, K_2_CO_3_, a 2° amine, r.t., 24 h, 21–92%; (iv) 1-bromo-4-methylpentane, DMF, K_2_CO_3_, 80 °C, overnight, 12%; (v) NaN_3_, DMF, r.t., 64 h, 95%; (vi) Ph_3_P, DMF-H_2_O, r.t., 91 h, 58%; (vii) 3-bromo-1-propanol, DMF, K_2_CO_3_, r.t., 2 d, 56%.

In the past decade, reports on scaffolds like benzopyridoindole [17], naphthylquinoline [25], neomycin [26], and quinolizinium [27] that can be used to develop a library of triplex-specific ligands have been scarce in the literature. However, such structural motifs have always attracted great interest due to their potential applications in the antigene strategy. We recently discovered a novel class of potent triplex-specific ligands (5-substituted 3,3′,4′,7-tetramethoxyflavonoids, Figure 1B) derived from a natural product, quercetin [28]. The amino-containing side chain at the 5-position of 3,3′,4′,7-tetramethoxyflavonoids is crucial for high binding affinities toward triplex DNA. A recent viscosity study in our lab showed that 5-substituted 3,3′,4′,7-tetramethoxyflavonoids most likely intercalate with triplex DNA due to the planarity of the flavonoid structure (unpublished data). Hence, the observed binding selectivity of these compounds for triplex DNA over duplex DNA probably results from their better aromatic surface stacking with DNA base triplets (more extended surface) than DNA base pairs. In light of this finding, we hypothesize that the four methoxy groups of 5-substituted 3,3′,4′,7-tetramethoxyflavonoids might not contribute to their binding with triplex DNA and hence can be removed from the structures. The removal of the four methoxy groups has apparent benefits. Specifically, we can simplify the synthetic steps and obtain a novel class of potential triplex-binding ligands with more drug-like properties (lower molecular weights and molecular volumes). In the present work, we report the further structure modification of flavonoid-based triplex-specific binding ligands (Scheme 1). The binding of newly developed 5-substituted flavones with triplex DNA was investigated by various biophysical methods, including thermal denaturation monitored by UV, gel electrophoresis, circular dichroism (CD), and isothermal titration calorimetry (ITC), while the binding mode of the ligands with triplex DNA was determined using viscosity studies. The inhibition of enzymatic activities by ligand-mediated triplex formation was demonstrated using a plasmid assay, and the cytotoxicity of the ligands against three cancer cell lines was measured by the MTT assay. The results presented in this paper unequivocally confirm the development of another class of highly specific and potent flavone-based triplex DNA-binding ligands from our laboratory.

## 2. Results and Discussion

### 2.1. Preparation of 5-Substituted Flavones

5-Substituted flavones can be readily synthesized using a three-step reaction scheme in good to excellent yields (Scheme 1). The reaction of 2′,6′-dihydroxyacetophenone with benzoyl chloride under basic conditions yielded 5-hydroxyflavone (**1**) via a modified Baker–Venkataraman reaction based on a published protocol [29]. The coupling of **1** with different dibromoalkanes (excess) was carried out in the presence of K_2_CO_3_ to yield compounds **2a**−**2c** following a modified approach [30]. Dibromoalkanes containing 2–4 carbons were used. The nucleophilic substitution of bromides (**2a**−**2c**) with a secondary amine gave the desired compounds (**3a**−**8c**). The symbols **a**, **b**, and **c** refer to compounds containing an ethylene [-(CH_2_)_2_-], a propylene [-(CH_2_)_3_-], and a butylene [-(CH_2_)_4_-] linker at the 5-position, respectively. The amino groups at the end of the side chains used in our study (Scheme 1) can be categorized into two types, including cyclic amines (**3a**−**6c**) and dialkyl amines (**7a**−**8c**). In addition, three analogs (**9b**, **10b**, and **11b**) were synthesized to study the end-group effect, containing an isopropyl group, a primary amino group, and a hydroxyl group at the end of the side chain, respectively. The reaction of **1** with 1-bromo-4-methylpentane yielded compound **9b**. The nucleophilic substitution of **2b** with sodium azide, followed by the reduction of the azido group using the Staudinger reaction, gave compound **10b**. Coupling **1** with 3-bromo-1-propanol yielded compound **11b**.

### 2.2. Thermal Stabilization of Triplex DNA by 5-Substituted Flavones

The collection of a library of 5-substituted flavones (**3a**−**11b**) allowed us to make a complete comparison of various factors (e.g., linker and end amino groups) that could affect triplex stabilization. The stabilization of triplex DNA by 5-substituted flavones was determined using thermal denaturation monitored by UV at 260, 280, and 284 nm [31]. The dissociation of triplex DNA with increasing temperatures often results in a biphasic hyperchromic UV transition. A transition at a lower temperature represents the dissociation of triplex DNA into duplex DNA and a single stand. A transition at a higher temperature represents the dissociation of duplex DNA into random coils. The melting temperature (T_m_) of DNA refers to the temperature at which 50% of DNA in a sample has denatured. The symbols T_m3→2_ and T_m2→1_ are designated as the melting temperature of triplex and duplex DNA, respectively. The ΔT_m_ is defined as the difference between the T_m_ values in the presence and absence of a ligand. To obtain a complete image of the effect of a ligand on the stabilization of triplex DNA, we chose an intermolecular polynucleotide triplex DNA, poly(dA)•2poly(dT), that is used as a model triplex for thermal denaturation, as it yields a well-separated biphasic transition. The wavelengths 280 and 284 nm were used for the cross-verification of triplex and duplex dissociation (280 nm for triplex and 284 nm for duplex) because the hyperchromicity of duplex and triplex melting profiles at these two wavelengths is dramatically different.

**Figure 2:**
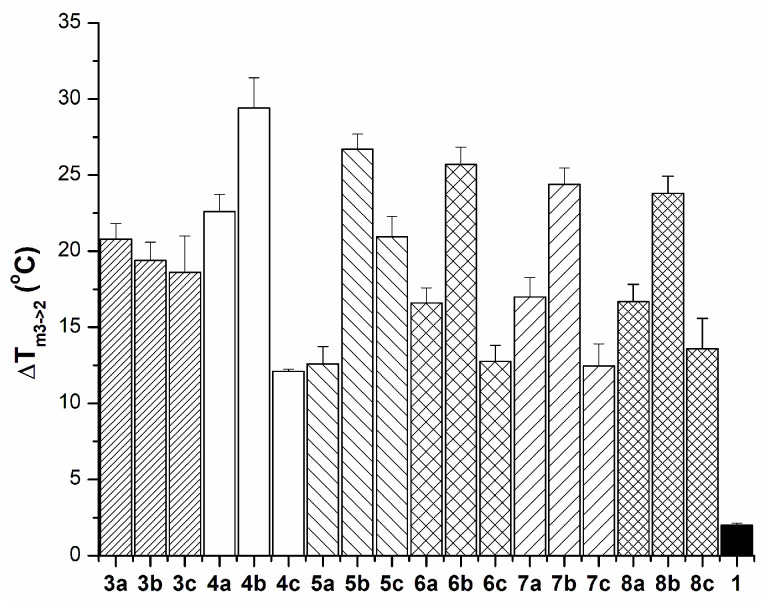
Changes in the UV melting temperatures (ΔT_m3→2_) of poly(dA)•2poly(dT) at 260 nm in the presence and absence of a 10 μM ligand (**1** and **3a**−**8c**). [DNA] = 15 µM/base triplet. Buffer: 10 mM sodium cacodylate; 150 mM KCl; pH 7.0. The symbol 3→2 refers to the dissociation of triplex DNA into duplex DNA.

First, the thermal denaturation experiments of poly(dA)•2poly(dT) (15 μM/base triplet) in the presence of a ligand (**1** and **3a**−**8c** containing a tertiary amino group) at a single-point concentration (10 μM) were conducted (Figure 2). Without a 5-substitued side chain, the control compound (5-hydroxyflavone, **1**) showed a minimal triplex stabilization effect. By contrast, all 5-substituted flavone derivatives (**3a**−**8c**) showed a significant stabilization effect [ΔT_m3→2_ > 19.4 °C for compounds with a propylene linker (**3b**−**8b**); ΔT_m3→2_ > 12.1 °C for compounds with a butylene linker (**3c**−**8c**); and ΔT_m3→2_ > 12.6 °C for compounds with an ethylene linker (**3a**−**8a**)] on poly(dA)•2poly(dT), indicating that the side chain is crucial for the binding of ligands to triplex DNA. Interestingly, all compounds (**3a**−**8c**) exhibited no stabilization effect on duplex DNA, poly(dA)•poly(dT), as the T_m2→1_ values remained around 72 °C. It is clear that the amino-containing side chains installed at the 5-position of flavones result in their remarkable selectivity for triplex over duplex DNA. In general, ligands with a propylene linker (three carbons) showed a more significant triplex stabilization effect than those with an ethylene (two carbons) or a butylene linker (four carbons). For instance, the ΔT_m3→2_ value of poly(dA)•2poly(dT) in the presence of compound **4b** was 29.4 °C, while compounds **4a** and **4c** stabilized triplex DNA at 22.6 °C and 12.1 °C, respectively. The observed trend suggests that the binding of triplex DNA by 5-substituted flavones is influenced by the position and orientation of the side chain, which most likely resides in a triplex groove. Compounds with a propylene linker (**4b**, **5b**, **6b**, **7b**, and **8b**) have an optimal fit into the binding pocket of triplex DNA. Compounds with a pyrrolidine group (**3a**, **3b**, and **3c**) are the only ones that do not follow the similar linker-length trend, suggesting that their binding to triplex DNA could be further affected by the position and interactions of the five-membered ring with triplex grooves.

As for the effect of tertiary amino groups, compounds with a six-membered amino-containing ring showed overall better triplex stabilization effects than compounds with a dialkylamino group. For instance, when compounds with the same propylene linker were compared, compounds **4b**, **5b**, and **6b** gave ΔT_m3→2_ values of 29.4 °C, 26.7 °C, and 25.7 °C, respectively. By contrast, compounds **7b** and **8b** showed ΔT_m3→2_ values of 24.4 °C and 23.8 °C, respectively. Compound **3b** with a pyrrolidine group gave the lowest ΔT_m3→2_ value (19.4 °C). Based on the thermal denaturation data, we conclude that all the tertiary amino groups used here can provide a strong triplex stabilization effect. Cyclic amino groups (especially six-membered rings), in general, are better than dialkyl amino groups for triplex binding when used as an end group in the side chain. Both observations are consistent with our published results on 5-substituted 3,3′,4′,7-tetramethoxyflavonoids containing a butylene linker. A direct comparison of the triplex stabilization effects of 5-substituted flavones and 5-substituted 3,3′,4′,7-tetramethoxyflavonoids [28] with the same linker and end amino groups leads to the following conclusions. (1) Most of the 5-substituted flavones with a butylene linker stabilize triplex DNA as strongly as the corresponding 5-substituted 3,3′,4′,7-tetramethoxyflavonoids except for the ones with a piperidine group (Supplementary Materials, Figure S85A). (2) Most of the 5-substituted flavones with a propylene linker stabilize triplex DNA much more strongly than the corresponding 5-substituted 3,3′,4′,7-tetramethoxyflavonoids except for the ones with a pyrrolidine group (Supplementary Materials, Figure S85B). This strongly supports our hypothesis that the four methoxy groups in 5-substituted 3,3′,4′,7-tetramethoxyflavonoids are not responsible for their binding to triplex DNA. For some ligands, such as the ones with a propylene linker, the methoxy groups even reduce their ability to bind to triplex DNA.

**Figure 3:**
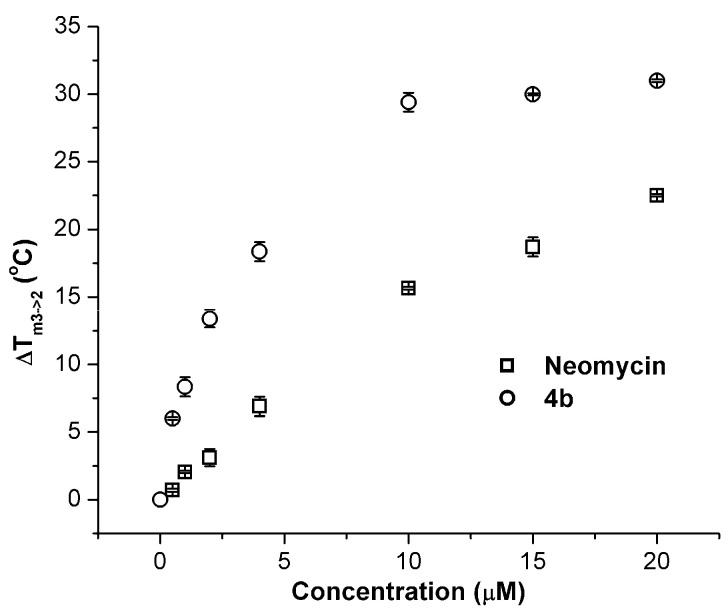
Comparison of changes in the UV melting temperatures (ΔT_m3→2_) of poly(dA)•2poly(dT) at 260 nm in the presence of **4b** and neomycin at various concentrations ranging from 0 to 20 μM. [DNA] = 15 µM/base triplet. Buffer: 10 mM sodium cacodylate; 150 mM KCl; pH 7.0. Error bars are shown with the hollow symbols (○ for **4b** and □ for neomycin).

The stabilization effect on poly(dA)•2poly(dT) by 5-substituted flavones was further confirmed with a concentration-dependent study. Compound **4b** was used as a representative ligand, and neomycin, a well-known triplex binder, was used as a control for comparison. The ΔT_m3→2_ values as a function of increasing ligand concentrations (0 to 20 μM) are shown in Figure 3. Both **4b** and neomycin stabilized poly(dA)•2poly(dT) in a concentration-dependent manner. Compound **4b** showed a much stronger stabilization effect than neomycin at each concentration used. For instance, at 4 μM, the ΔT_m3→2_ value of poly(dA)•2poly(dT) in the presence of compound **4b** was 18.4 °C, 11.5 °C higher than that of neomycin. Strikingly, compound **4b** even stabilized triplex DNA at low concentrations (e.g., 0.5 μM, ΔT_m3→2_ = 6.0 °C), where neomycin had no triplex stabilization effect. The ΔT_m3→2_ values with compound **4b** start to exhibit a clear plateau at a 10 μM ligand concentration, suggesting the saturation of binding sites by **4b** on triplex DNA.

### 2.3. The Amino Groups in the 5-Substituted Flavones Are Crucial for Triplex Binding

**Figure 4:**
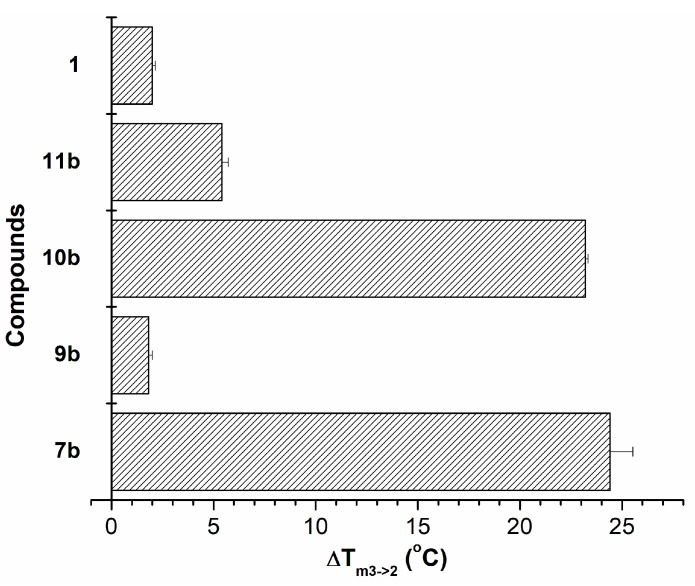
Comparison of changes in the UV melting temperatures (ΔT_m3→2_) of poly(dA)•2poly(dT) at 260 nm in the presence of **1**, **7b**, **9b**, **10b**, or **11b** at 10 μM. [DNA] = 15 µM/base triplet. Buffer: 10 mM sodium cacodylate; 150 mM KCl; pH 7.0.

To determine whether the tertiary amino groups of the 5-substituted flavones were responsible for the observed triplex stabilization effect, we carried out thermal denaturation experiments (Figure 4) of poly(dA)•2poly(dT) (15 μM/base triplet) in the presence of **9b**, **10b**, or **11b** (10 μM). All three compounds contained the same (propylene) linker for better comparison. Compound **9b**, with a pure alkyl side chain, did not show any triplex stabilization effect, indicating that the tertiary amino groups at the end of the side chains are essential for triplex binding. The amino groups could interact with triplex DNA via H-bonding or electrostatic interactions as they could be partially protonated under physiological conditions. Compound **10b**, with a primary amino group in the side chain, showed a strong triplex stabilization effect, with a ΔT_m3→2_ value of 23.2 °C. This value is slightly lower than those for compounds with a dialkyl amino group (**7b** and **8b**) and a few degrees lower than those for compounds with a cyclic amino group (**4b**, **5b**, and **6b**; Figure 2). Tertiary amines are stronger bases than primary amines; therefore, tertiary amino groups could carry more partial positive charges under physiological conditions, leading to stronger electrostatic interactions with triplex DNA. Replacing the amino group with a hydroxyl group drastically decreased the triplex stabilization effect. Compound **11b**, with an OH group at the end of the side chain, only increased the ΔT_m3→2_ value by 5.4 °C, at least 17.8 °C less than compounds **10b** and **7b** (Figure 4). Since the OH group can be a hydrogen-bond donor or acceptor, similarly to the NH_2_ group, the observed large discrepancy in T_m3→2_ increments between **11b** and **10b** most likely results from the electrostatic interactions of the partially charged amino group with triplex DNA under physiological conditions.

### 2.4. Inducing Triplex Formation by 5-Substituted Flavones Under Salt-Deficient Conditions

**Figure 5:**
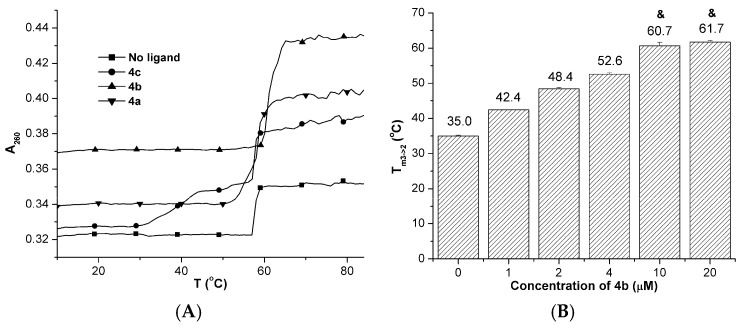
(**A**) Representative melting profiles of poly(dA)•2poly(dT) at 260 nm in the absence and presence of **4a**, **4b**, and **4c** at 10 μM under salt-deficient conditions. For clarity, the *y*-axis is offset. From left to right, the first transition refers to the dissociation of poly(dA)•2poly(dT) into poly(dA)•poly(dT) and poly(dT) and the second transition refers to the dissociation of poly(dA)•poly(dT) into poly(dA) and poly(dT). (**B**) Bar graphs showing the T_m_ values of poly(dA)•2poly(dT) in the absence and presence of **4b** at various concentrations ranging from 0 to 20 μM. The symbol & refers to a 3→1 transition. [DNA] = 15 µM/base triplet. Buffer: 10 mM lithium cacodylate; pH 7.0.

The strong triplex stabilization effect of 5-substituted flavones mentioned in the sections above prompted us to investigate whether they can induce triplex DNA formation under salt-deficient conditions, for which a lithium buffer (10 mM lithium cacodylate, pH 7.0) was used. Thermal denaturation experiments of poly(dA)•2poly(dT) in the absence and presence of **4a**, **4b**, and **4c** (10 μM) were conducted. These compounds were chosen because **4b** showed the best triplex stabilization effect. The melting profiles are shown in Figure 5A. Under this buffer condition, a DNA solution containing 1:1 poly(dA)•2poly(dT) and poly(dT) only yielded a monophasic transition with a T_m_ value of 58 °C, representing the dissociation of duplex DNA [poly(dA)•poly(dT)] into random coils. It is clear that triplex DNA cannot be formed under salt-deficient conditions. In the presence of 10 μM **4a**, a biphasic transition was observed. The first transition, with a T_m_ value of 55 °C, represents the dissociation of triplex into duplex DNA. The second transition, with a T_m_ value of 58 °C, indicates the dissociation temperature of the duplex into random coils. Compound **4a** can effectively induce the formation of triplex DNA under salt-deficient conditions; however, it cannot stabilize duplex DNA. A similar biphasic transition of poly(dA)•2poly(dT) was observed in the presence of **4c** (10 μM). The T_m_s of the first and second transition were 40 °C and 58 °C, respectively. The binding of **4c** to triplex DNA was much less efficient than **4a**, as the difference in T_m3→2_ values was 15 °C. Interestingly, a monophasic transition (60.7 °C) was observed for poly(dA)•2poly(dT) in the presence of **4b** (10 μM), which represents the direct dissociation of triplex DNA into random coils (refers to 3→1) without it passing through the commonly seen duplex dissociation stage. The 3→1 transition was also confirmed by examining the melting profiles at 280 nm and 284 nm. Further evidence of the 3→1 transition was obtained by conducting thermal denaturation of poly(dA)•2poly(dT) in the presence of **4b** with increasing concentrations from 0 to 20 μM under the same conditions (Figure 5B). Biphasic transitions of the melting profiles were observed from 0 to 4 μM of **4b**. The T_m3→2_ values were 42.4, 48.4, and 52.6 °C for the ligand concentrations of 1, 2 and 4 μM, respectively. At these concentrations, the dissociation of duplex DNA was clearly observed at approximately 58 °C. At 10 and 20 μM of **4b**, only monophasic transitions were observed, suggesting that with increasing concentrations of **4b**, the 3→2 transition gradually becomes a 3→1 transition. This type of dissociation event is normally observed with extremely potent triplex-binding ligands. Hélène and his co-workers reported a similar 3→1 transition on a rationally designed triplex-binding ligand, BQQ [23]. The results presented here unambiguously suggest that 5-substituted flavones are potent triplex-binding ligands as they can induce triplex formation even at low concentrations under salt-deficient conditions. In addition, the order of linker length for optimal triplex binding under salt-deficient conditions is propylene (**4b**) > ethylene (**4a**) > butylene (**4c**), which is consistent with the results of thermal denaturation shown in the sections above.

### 2.5. Visualization of Ligand-Induced Triplex Formation via PAGE

**Figure 6:**
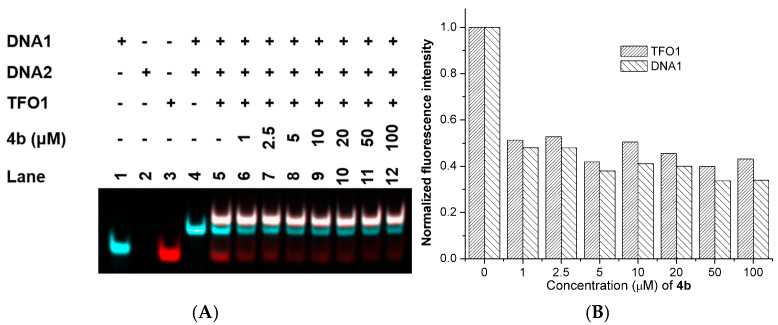
(**A**) The gel shift assay shows the formation of triplex DNA (formed by **DNA1**, **DNA2**, and **TFO1**) as a function of increasing concentrations of **4b**. Buffer: 90 mM Tris–acetate; pH 5.0. (**B**) Normalized fluorescence intensity of the duplex bands (Cy3) and the TFO bands (Cy5) in the presence of **4b** at various concentrations ranging from 0 to 100 μM.

The formation of triplex DNA induced by 5-substituted flavones was further visualized using a gel shift assay. The mobility of triplex, duplex, and single-stranded DNA is distinctive and can be easily differentiated by polyacrylamide gel electrophoresis (PAGE). In this experiment, we used a Cy3-labeled oligonucleotide DNA duplex template (**DNA1** with Cy3 and its complementary strand **DNA2**) and a Cy5-labeled triplex-forming oligonucleotide (**TFO1**). Compound **4b** was chosen as a representative ligand with a concentration range from 1 to 100 μM. As shown in Figure 6A, the Cy3-labeled oligonucleotide (**DNA1**, lane 1) and the Cy5-labeled **TFO1** (lane 3) have a cyan and red color, respectively. **DNA2**, without any labeling, has no fluorescent signals (lane 2). Because the gel imager only provides false colors for the fluorescent dyes, both cyan and red colors were selected for Cy3 and Cy5, respectively. The formation of the duplex is shown as a cyan band in lane 4. The oligonucleotide triplex used here contains C^+^•GC triplets that are known to prefer lower pHs for the formation of triple-helical structures; therefore, a Tris–acetate buffer at a pH of 5.0 was used. Lane 5 shows the formation of the triplex (for the triplex structure, see Figure 1C) as a band with a whitish color, which has a lower mobility than the duplex band. Small amounts of the duplex and **TFO1** are still present in lane 4, probably due to the incomplete formation of triplex DNA in the solution. The observation of the residual duplex and **TFO1** bands is reasonable because triplex DNA is not as stable as duplex DNA. The fluorescence intensities of the duplex band and the **TFO1** band in the absence of **4b** (lane 5) were set to 100%. In the presence of 1 μM of **4b**, the intensities of the duplex band and the **TFO1** band (Lane 6) immediately decreased by 48% and 51%, respectively, compared with those in Lane 5 (Figure 6B). In general, the intensities of the duplex band and the **TFO1** band (Lanes 6–12) decreased as a function of increasing concentrations of **4b**. It is to be noted that we did not observe the whitish triplex bands getting thicker with an increasing concentration of **4b**, probably because the intercalation of **4b** into triplex DNA (for an explanation, see the following section) interfered with the fluorescent signals. Nevertheless, these observations clearly indicate that the formation of triplex DNA is promoted by **4b**.

### 2.6. Binding of Triplex DNA with 5-Substituted Flavones via Intercalation

**Figure 7:**
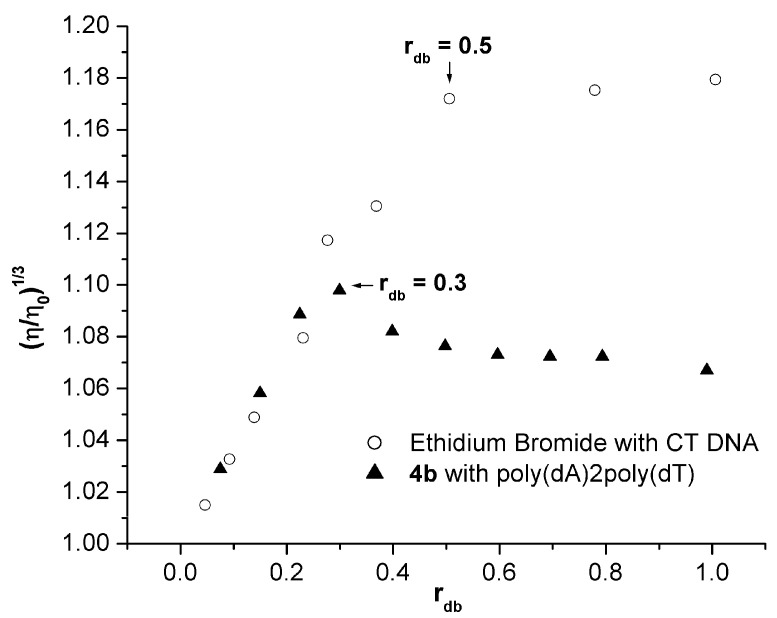
Viscosity changes in poly(dA)•2poly(dT) (▲) and calf thymus DNA (○) with increasing concentrations of **4b** and ethidium bromide (**EB**) ranging from 0 mM to 1.1 mM, respectively.

The main non-covalent ligand-to-DNA binding modes are limited to intercalation (π–π stacking) and groove binding due to the rigidity of DNA structures [32]. Non-specific electrostatic interactions are plausible and are often seen in metal cations [33]. The binding modes of intercalation and groove binding can be experimentally differentiated using the viscosity measurement [27,34,35]. The viscosity of DNA (duplex or triplex) increases proportionally as a function of increasing concentrations of an intercalator. The insertion of intercalators into DNA base pairs (or triplets) can elongate the length of DNA and subsequently make the DNA solution more viscous. Typically, the titration of an intercalator into DNA results in a slope of 0.5–1 in a viscosity plot. By contrast, groove binders bind into DNA grooves, having no effect on DNA length; therefore, the viscosity of the DNA solution remains unchanged. The difference in the viscosity changes in DNA solutions due to intercalators and groove binders can be easily measured using an Ostwald-type viscometer. In our experiments, a calf thymus (CT) DNA solution (1 mM in base pairs) was used to validate the viscosity assay, titrated with ethidium bromide (**EB**, a known intercalator) at various concentrations ranging from 0 to 1.1 mM. As shown in Figure 7, the viscosity of the CT DNA solution increased proportionally with increasing concentrations of **EB**, unambiguously indicating the intercalation of **EB** into duplex DNA. A clear plateau was observed at approximately 1:2 ligand-to-DNA base pairs, implying that at the saturation point, one **EB** was bound with two DNA base pairs. Poly(dA)•2poly(dT) was used in the viscosity study for triplex DNA, as longer polynucleotide triplexes should yield more distinct viscosity changes than short oligonucleotide triplexes. Here, compound **4b** was used as a representative ligand. The viscosity of the triplex DNA solution (0.5 mM in base triplets) varied linearly with increasing concentrations of **4b** until the **4b**/DNA ratio was above 0.3, at which point a clear plateau was observed (Figure 7). This observation suggests that the binding mode of 5-substituted flavones with triplex DNA is indeed intercalation. The saturation point of the binding of **4b** to poly(dA)•2poly(dT) was approximately one ligand per three DNA base triplets. The ligand–DNA ratios at the saturation points for both **EB** and **4b** are consistent with previous reports on intercalation based on the neighbor-exclusion principle [36]. The side chain present in **4b** leads to a larger ligand-to-DNA ratio at the saturation point. Our viscosity results experimentally prove that the significant thermal stabilization of triplex DNA by 5-substituted flavones results from the intercalation of the flavone moiety between DNA triplets. The side chain at the 5-position must reside in one of the three triplex grooves and be guided by intercalation, providing strong contacts with the functional groups in the groove. As indicated in the sections above, the length and end amino groups of a side chain are crucial to the binding of 5-substituted flavones with triplex DNA. The confirmation of the intercalation binding mode also explains the previous observation that 5-substituted flavones thermally stabilize triplex DNA better than (or at a level comparable to) 5-substituted 3,3′,4′,7-tetramethoxyflavonoids, depending on the linker length. The four methoxy groups of 5-substituted 3,3′,4′,7-tetramethoxyflavonoids could reduce the efficacy of their intercalation to base triplets due to steric hindrance. In addition, electron-donating methoxy groups could increase the electron density of the flavone rings, leading to less favorable interactions with the electron-rich aromatic nucleobases in DNA [37].

### 2.7. Thermodynamic Parameters Determined by ITC

The thermodynamic parameters (e.g., binding enthalpy and binding affinity) of a ligand binding to DNA can be directly measured using isothermal titration calorimetry (ITC) [28,38,39], providing insights into the ligand–DNA binding mode. In a typical ITC experiment, small aliquots of the ligand are injected into a DNA solution. The heat released or gained during each injection is integrated, corrected by subtracting the blank data (heat of dilution from titrations of the same ligand into a buffer solution), and plotted as a function of the molar ratio of ligand to DNA. The resulting curve is fitted with a preset binding model to obtain various parameters, such as the dissociation constant (K_d_), the number of binding sites, enthalpy, and entropy.

**Figure 8:**
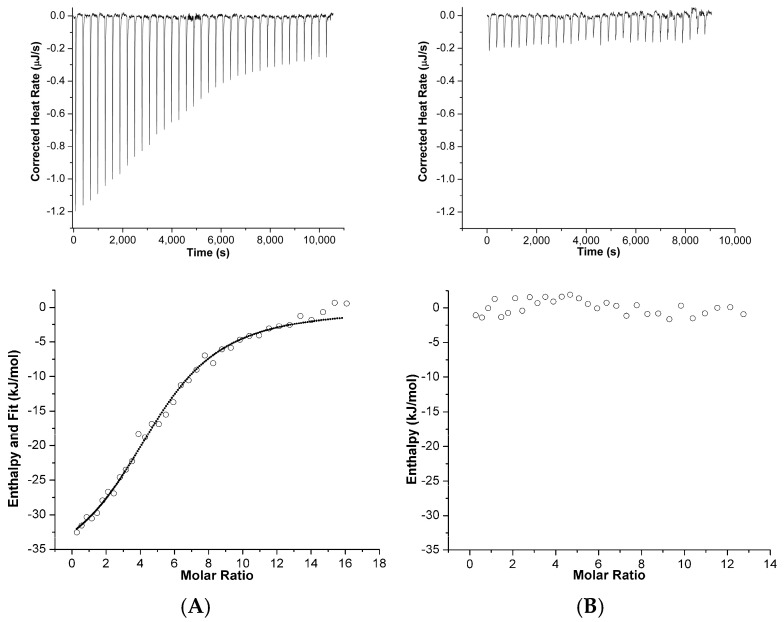
Upper panels: ITC profiles of **DNA3** (10 µM) (**A**) and **DNA4** (10 μM) (**B**) titrated with **4b** (100 µM) in a 10 mM sodium cacodylate buffer with 100 mM NaCl and pH 7.0 at 15 °C. Lower panels: The dotted lines represent the corrected injection heat values plotted as a function of the [**4b**]/[**DNA**] molar ratio. The smooth solid line reflects the calculated fit of the data using the independent model from the software NanoAnalyze. Downward peaks represent an exothermic event.

In our experiments, four ligands, including **1**, **4a**, **4b**, and **4c**, were chosen for comparison. Two oligonucleotides (**DNA3** and **DNA4**) were also used. **DNA3** contains three 15-nucleotide-long triplex-forming regions tethered by two four-thymidine loops, which fold back to form an intramolecular triplex DNA (Figure 1C) with a pyrimidine motif. **DNA4** has the same sequence as **DNA3** but does not contain the second four-thymidine loop and any of the nucleotides after it, meaning that it can only fold into an intramolecular duplex (Figure 1C). Figure 8 shows the representative ITC profiles (upper panels) resulting from a series of injections of **4b** into a solution of **DNA3** (A) and **DNA4** (B) in Na^+^ at 15 °C. This temperature was chosen to ensure the formation of triplex DNA. The heat of dilution collected from the injections of **4b** into the Na^+^ buffer under the same experimental conditions was used for baseline subtraction. The dotted lines (Figure 8, lower panels) represent the corrected injection heat values (obtained by subtracting the blank data for corrections of dilution effects) plotted against the [**4b**]/[DNA] molar ratio. The binding isotherms of ligands with DNA can be fitted with various binding models provided in the TA Instrument’s NanoAnalyze software (version 3.8.0) to achieve the best fit. At 15 °C, the binding isotherm of **4b** with **DNA3** (a triplex—Figure 8A, lower panel) best fit an independent model with a 95% confidence level and provided a dissociation constant (K_d_) of (1.01 ± 0.20) × 10^−5^ M (Table 1). The independent model used here models the interaction of ligands with a macromolecule that has either one binding site or multiple equivalent binding sites. On the other hand, the addition of **4b** into **DNA4** only yielded an ITC profile similar to the heat of dilution that could not be fitted with any model. The side-by-side comparison shown in Figure 8 clearly indicates that the 5-substituted flavones (**4b**) strongly interacted with triplex DNA while having no effect on duplex DNA, further validating the thermal denaturation results.

In addition, the titration of **1** into **DNA3** (a triplex) also yielded an ITC profile similar to the heat of dilution (Supplementary Materials, Figure S1), confirming that the amino-containing side chain is important for the binding of a ligand to triplex DNA. We also carried out the titration of **4a** or **4c** into a solution of **DNA3**. Both ligands had a similarly shaped ITC profile (Supplementary Materials, Figures S2 and S3) that could be fitted with the independent model. The dissociation constants (K_d_s) for **4a** and **4c** were (2.10 ± 1.12) × 10^−5^ M and (3.08 ± 1.26) × 10^−5^ M, respectively (Table 1). Compound **4b** with a propylene linker had the best binding affinity (lowest K_d_ value) for triplex DNA compared to **4a** and **4c**, which is consistent with the results from the thermal denaturation experiments. Compound **4c** with a butylene linker showed the smallest binding affinity value, implying that the longer alkyl chain may cause steric hindrance in the binding pocket.

The binding events of 5-substituted flavones (**4a**, **4b**, and **4c**) with triplex DNA (**DNA3**) are all enthalpy-driven with negative TΔS values (entropy unfavorable). All three ligands bind to **DNA3** with a free energy (ΔG) value of approximately −25 kJ mol^−1^. Compounds **4a** and **4c** bind to **DNA3** with more positive entropic values than **4b**, indicating that the linker length is crucial for the optimal binding of 5-substituted flavones with triplex DNA. A previous survey of thermodynamic signatures for ligand–DNA binding modes by Chaires [40] suggested that intercalation is predominantly enthalpically driven, which aligns with the binding mode determined by the viscosity studies. The π–π stacking and H-bonding interactions between an intercalator and base triplets, as well as the potential electrostatic interactions between partially charged amino groups and DNA backbones, contribute to the favorable enthalpic values. Intercalation lengthens and stiffens DNA, leading to unfavorable entropic changes.

The overall stoichiometry (n-value) for the binding of the ligand was determined to be approximately three to six ligands per DNA (Table 1). The n-value of **4b** with **DNA3** is 6, implying that six ligands bind to one triplex DNA. Given that there are 15 base triplets in **DNA3**, the n-value of 6 is equivalent to each ligand binding every 2.5 base triplets, which is close to the stochiometric ratio observed at the saturation point for **4b** in the viscosity study. The stoichiometric values for **4a** and **4c** are different from that for **4b**, probably resulting from the different linker lengths. However, these n-values are well within the range of binding stoichiometry needed for intercalation [27].

### 2.8. Binding of Triplex DNA with 5-Substituted Flavones Determined by Circular Dichroism

**Figure 9:**
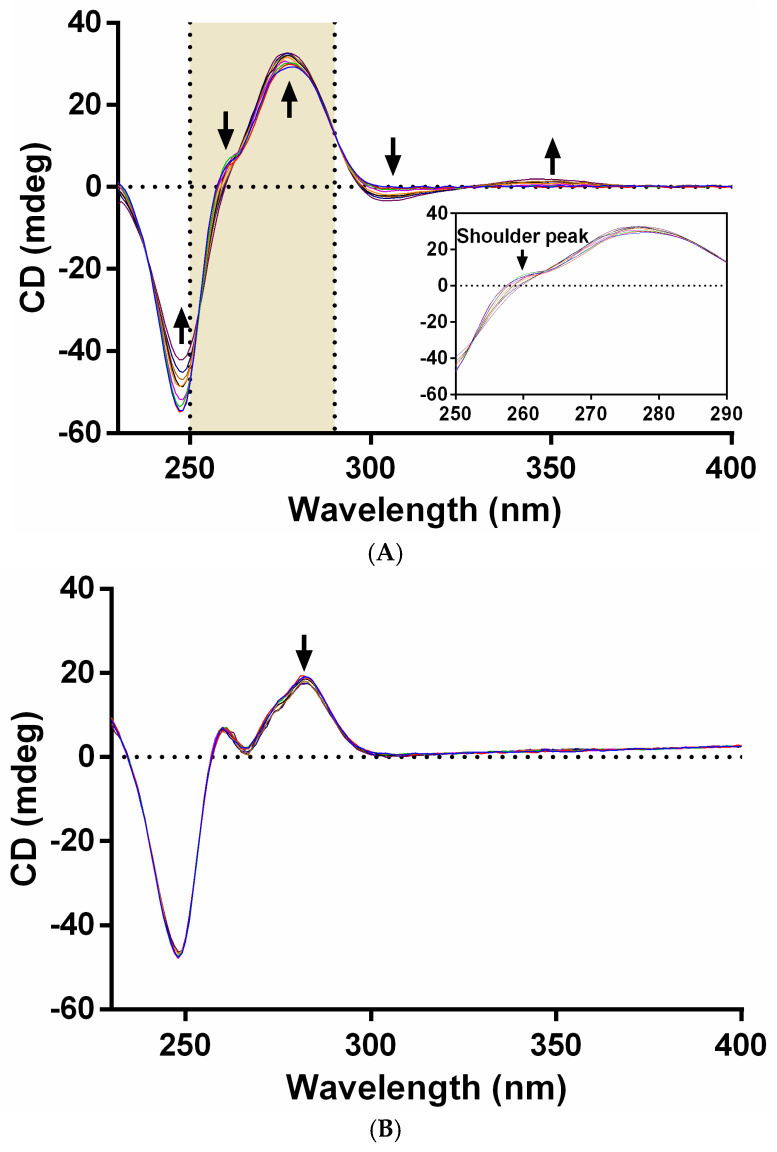
(**A**) CD spectra of **DNA3** (triplex DNA) recorded when titrated with **4b**; (**B**) CD spectra of **DNA4** (duplex DNA) recorded when titrated with **4b**. The direction of the arrows indicates the continuous changes at a specific wavelength in the CD spectra with each incremental amount of **4b** ranging from 0 to 50 μM (The CD curves are shown in different colors.). The inserted graph in (**A**) represents the zoom region from 250 to 290 nm (highlighted with a moccasin color), showing the changes in the shoulder peak.

The interactions of DNA with a ligand often result in conformational changes that can be readily measured by circular dichroism (CD). In our study, we chose **4b** as a representative compound and titrated it against a solution of **DNA3** (triplex DNA, 5 μM) and **DNA4** (duplex DNA, 5 μM) in a sodium cacodylate buffer (pH 7) at 15 °C, respectively. As shown in Figure 9A, the CD spectrum of **DNA3** alone shows a negative peak at 247 nm, a positive peak at 278 nm, and a positive shoulder peak at 260 nm, a clear indication of a DNA triple-helical structure in the solution [28]. The titration of **4b** into **DNA3** did not lead to drastic shape changes in the CD spectra, sometimes observed in ligand and G-quadruplex DNA interactions [41]. However, distinct conformational changes were observed. With the increase in the concentrations of **4b** from 0 to 50 μM, the intensities of the negative peak at 247 nm gradually decreased, and the intensities of the positive peak at 278 nm increased. More significantly, the shoulder peak at 260 nm gradually disappeared (see the inserted graph in Figure 9A). Two clear isoelliptic points at 252 nm and 263 nm were observed, suggestive of the equilibrium between free and bound ligands present in the solution. The binding of **4b** to **DNA3** was further characterized by the gradual appearance of a negative peak between 300 and 325 nm and a positive peak between 330 and 370 nm with increasing concentrations of **4b**. Compound **4b** is an achiral molecule that is CD-inactive. Upon its binding to **DNA3**, the rotation of the phenyl group of **4b** is restricted, and hence, it is present in an asymmetric environment, leading to the induction of CD signals for bound ligand molecules. By contrast, the CD spectrum of **DNA4** (duplex DNA) alone shows a negative peak at 248 nm, a small positive peak at 260 nm, and a large positive peak at 282 nm (Figure 9B), which is different from that of **DNA3** (triplex DNA). The titration of **4b** resulted in minimal conformational changes in **DNA4** as the CD spectra were almost identical with increasing concentrations of **4b**. Only a neglectable decrease in the peak intensities at 282 nm was noticed. Compound **4b** brought much less perturbation to the CD spectra of **DNA4**, implying weak-to-no interactions of **4b** with duplex DNA. The side-by-side comparison of the CD data from the titration of **4b** into duplex and triplex DNA acts as another cross-verification, confirming that **4b** binds strongly to triplex DNA while having a minimal effect on duplex DNA. The distinct conformational changes in triplex DNA upon the addition of **4b** also support the intercalation binding mode determined by the viscosity study. In general, intercalation unwinds and lengthens DNA, leading to more significant conformational changes in its structure than with groove binding [42].

### 2.9. Inhibition of Restriction Enzyme Activity via Ligand-Mediated Triplex Formation

**Figure 10:**
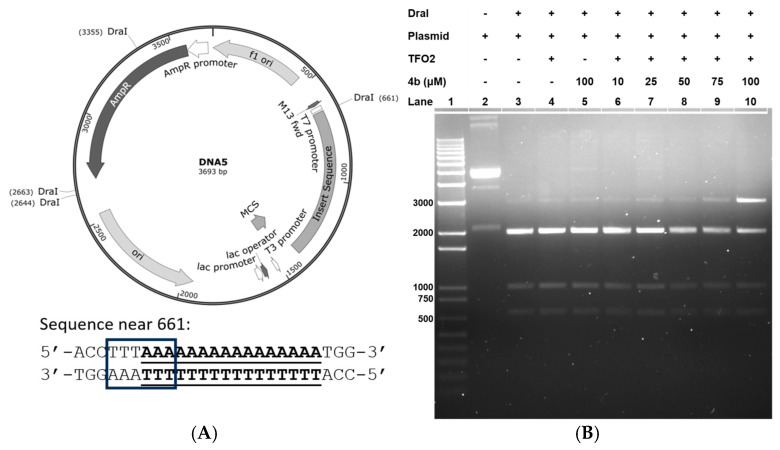
(**A**) The constructed 3693 bp plasmid (**DNA5**) contains four *DraI* cleavage sites at positions 661, 2644, 2663, and 3355. The DNA sequence near position 661 contains a 16-mer deoxyadenosine–thymidine sequence as the triplex-forming region (labeled as underlined bold text). The *DraI* cleavage site at position 661 is indicated by the rectangular box. (**B**) Agarose gel shows the cleavage of the plasmid (**DNA5**) by *DraI* under various conditions as indicated.

Upon confirmation of the selective binding of 5-substituted flavones to triplex DNA with high affinities using various biophysical tools, we further investigated their ability to assist TFOs in inhibiting enzymatic activities. Ideally, if a ligand induces triplex formation at a binding site of an enzyme, the formed triple-helical structure can block the binding of the enzyme to the same region and subsequently inhibit its activity. It has been reported that triplex structures can inhibit the activities of nucleic acid processing enzymes (e.g., DNA and RNA polymerases, restriction enzymes) [43]. The inhibited enzymatic effect could be restored upon the removal of the triplex stabilizing agent [44]. As a demonstration, compound **4b** was chosen as a representative ligand, and a plasmid DNA (**DNA5**) was designed and constructed as a substrate of the restriction endonuclease *DraI*. The plasmid known as pBluescript II SK(+) containing three *DraI* sites was used as the initial template for modification. *DraI* cleaves specifically at the sites with a sequence of 5′-TTT↓AAA-3′. The plasmid [pBluescript II SK(+)] was modified by inserting a 16 bp AT tract (oligopurine–oligopyrimidine) to create a triplex-forming region near the T7 promoter, followed by an mcherry sequence, to yield **DNA5** (Figure 10A; for the entire sequence, see Supplementary Materials, Figure S4). By design, **DNA5** includes a new *DraI* cleavage site located precisely at the triplex-forming region (Figure 10A). The complete digestion of the plasmid gave four fragments with sizes of 19 bp, 692 bp, 999 bp, and 1983 bp. The 19 bp fragment was too short to be observed on the nondenaturing agarose gel. The triplex formation at the T7 promoter, in theory, should inhibit the cleavage by *DraI* at this site, leading to a larger fragment with a size of 2982 bp (999 bp + 1983 bp) that could be readily identified using the agarose gel. The results of the digestion of **DNA5** by *DraI* under various conditions are shown in Figure 10B. Lane 1 shows the ladder as a reference, and lane 2 shows the plasmid **DNA5**. Lane 3 represents the digestion of **DNA5** by *DraI*. As expected, three fragments with sizes of 692 bp, 999 bp, and 1983 bp were observed. Lane 4 represents the digestion of **DNA5** by *DraI* in the presence of a TFO (a 16-mer oligothymidine, **TFO2**). A similar fragment pattern was observed in lanes 3 and 4, suggesting that under this condition, **TFO2** could not efficiently form a triplex structure at the T7 promoter to inhibit *DraI* activity. Lane 5 represents the digestion of **DNA5** by *DraI* in the presence of 100 μM of **4b**. The same three fragments, as shown in lane 3, were observed, suggesting that **4b** alone could not inhibit *DraI* activity. Lanes 6–10 show the digestion of **DNA5** by *DraI* in the presence of **TFO2** as a function of increasing **4b** concentrations. A larger band with a size of approximately 3000 bp started to emerge. The densitometric values of this band increased with increasing concentrations of **4b**, indirectly proving that ligand-mediated triplex formation occurred at the T7 promoter. A detailed densitometric study of each band is shown in the Supplementary Materials, Figure S5. In general, there was no overall densitometric change in the 693 bp fragment, as the cleavage at its sites was not affected by triplex formation. However, the band intensities of 999 bp and 1983 bp fragments decreased as a function of increasing concentrations of **4b** (lanes 6–10), while the band intensity of the 2982 bp fragment increased. In the presence of **TFO2** and 100 μM of **4b**, a large amount of the 2982 bp fragment appeared in lane 10. Taken together, we conclude that these observations must result from the triplex formation induced by **4b**. The results presented here suggest that it is plausible to use 5-substituted flavones as antigene enhancers to inhibit enzymatic activities.

### 2.10. 5-Substituted Flavones Are Cytotoxic Against Various Cancer Cells

A previous statistical analysis of the biological activities of 63 human colorectal tumor extracts showed that tumor extracts had an increase in triplex DNA-binding activity compared to normal tissue extracts. The increase in triplex DNA-binding activity in cancer cells is linked to the overall reduced survival rate of patients [45]. Because we have proven that 5-substituted flavones are a class of strong triplex-specific binding ligands, it is important to investigate their cytotoxicity against various cancer cell lines. In our experiments, we chose three types of cancer cells, including two colorectal cancer cell lines (HT-29 and HCT116) and one leukemia cancer cell line (HL-60). A single-point assay of 50 μM of the ligands was first conducted to screen the ligands with a meaningful cytotoxicity value for further investigations using the MTT assay. This colorimetric assay is based on a reduction reaction of tetrazolium dye [MTT, 3-(4,5-dimethylthiazol)-2-yl)-2,5-diphenyltetrazolium bromide] into formazan by cellular reductases. Because only live cells produce active reductases, the production of formazan (measured by absorbance) is proportional to the number of live cells. In our concentration-dependent experiments, six ligands, including **4a**, **4b**, **4c**, **8a**, **8b**, and **8c**, were then selected for the determination of IC_50_ values, which were calculated using the standard method of GraphPad Prism with a 95% confidence interval. Compound **1** was used as a control. The IC_50_ values for each ligand are shown in Table 2, and the dose-dependent curves are shown in Supplementary Materials, Figures S6–S8. Compound **1** (without a side chain) shows very weak cytotoxicity, with IC_50_ values larger than 50 μM across all three cancer cell lines. Several ligands had strong cytotoxicity against these cancer cell lines, with IC_50_ values in the low micromolar concentration range. For instance, compounds **4a**, **4b**, and **8b** showed IC_50_ values of 8.8, 6.7, and 4.6 μM for HL-60, respectively. Compounds **4b** and **8b** showed IC_50_ values of 14.2 μM and 12.5 μM for HT-29, respectively. For HCT116, only compound **8b** showed a relatively low IC_50_ value of 7.9 μM. Overall, compound **8b** had the best cytotoxicity toward all three cancer cell lines used here. It is interesting that both **4b** and **8b**, showing remarkable cytotoxicity toward cancer cells, have a propylene linker, following the same trend as that in the thermal denaturation data, namely, that compounds with a propylene linker have the best triplex stabilization effect. However, no direct correlation between triplex stabilization and cytotoxicity should be assumed at this point. The positive cell viability data presented here warrant further investigation into this subject in the future.

## 3. Experimental Section

### 3.1. Materials and General Methods

All chemicals for synthesis were purchased from MilliporeSigma (St. Louis, MO, USA) or Fisher Scientific (Norristown, PA, USA) and used without further purification. DNA oligonucleotides were purchased from Fisher Oligos or Integrated DNA Technologies (IDT). Polynucleotides were purchased from MilliporeSigma. The concentrations of polynucleotides were determined spectrophotometrically using the following extinction coefficients (in units of mol of nucleotide/L^−1^ cm^−1^): ε_260_ = 6000 for poly(dA)●poly(dT); ε_265_ = 9000 for poly(dT); and ε_260_ = 6600 for calf thymus (CT) DNA. The concentrations of oligonucleotides were determined spectrophotometrically using the following extinction coefficients (in units of mol of strand/L^−1^ cm^−1^) obtained from OligoAnalyzer (www.idtdna.com, Integrated DNA Technologies, Coralville, IA, USA): ε_260_ = 368,100 for **DNA1** (5′-/Cy3/ATGGCAAAAAAAAGGAGAAGCTGACCACCTGACTA-3′); ε_260_ = 312,100 for **DNA2** (5′-TAGTCAGGTGGTCAGCTTCTCCTTTTTTTTGCCAT-3′); ε_260_ = 174,900 for **TFO1** (5′-/Cy5/CTTTCTTTTTTTTTCCTCTTC-3′); ε_260_ = 483,700 for **DNA3** (5′-GAAAAAAAAAAAAAGTTTTCTTTTTTTTTTTTCTTTTCTTTTTTTTTTTTTC-3′); ε_260_ = 331,600 for **DNA4** (5′-GAAAAAAAAAAAAAGTTTTCTTTTTTTTTTTTTC-3′); and ε_260_ = 130,200 for **TFO2** (5′-TTTTTTTTTTTTTTTT-3′). UV spectra were recorded on a Varian Cary-100 Bio UV-Vis spectrophotometer (Varian Medical Systems, Palo Alto, CA, USA) equipped with a thermoelectrically controlled 2 × 6 cell holder. Isothermal titration calorimetry measurements were performed on a TA Instruments Affinity ITC LV (Waters, New Castle, DE, USA). Circular dichroism spectra were recorded on a JASCO J-810 spectropolarimeter (JASCO Inc., Easton, MD, USA) using a quartz cuvette with a 1 cm optical path length. ^1^H-NMR and ^13^C-NMR spectra were recorded on a JEOL ECA 600 MHz FT-NMR spectrometer (JEOL, Pleasanton, CA, USA). MS (DART-TOF) spectra for small molecules were recorded on a JEOL Accu TOF LC^TM^ time-of-flight mass spectrometer equipped with a DART^TM^ ion source or an Agilent LC-qTOF (Agilent Technologies, Santa Clara, CA, USA) with electrospray ionization (ESI). Viscosity data were collected using an Ostwald-type viscometer. The plasmid DNA (**DNA5**) was designed using SnapGene (Boston, MA, USA) and synthesized by GenScript Biotech (Piscataway, NJ, USA).

### 3.2. Synthesis

#### 3.2.1. Synthesis of Compound **1**

The synthesis of **1** is based on a published protocol [29]. To a dry acetone solution (100 mL) of 2′,6′-dihydroxyacetophenone (3.0 g, 20 mmol), anhydrous potassium carbonate (13.8 g, 100 mmol) was added. The resulting solution was stirred at room temperature for 10 min. Benzoyl chloride (2.3 mL, 20 mmol) was added, and the mixture was refluxed for 24 h under nitrogen. The reaction was cooled back down to room temperature before water (30 mL) was added, and the acetone was evaporated using a rotary evaporator. The reaction mixture was extracted using ethyl acetate (100 mL) and then rinsed with water (10 mL) and brine (10 mL × 4). The organic layer was subsequently dried over anhydrous sodium sulfate and concentrated under vacuum. Flash column chromatography of the residue [silica gel, ethyl acetate/hexane (92.5/7.5, *v*/*v*)] yielded compound **1** (1.9 g, 40%) as a yellow solid. IR (KBr) *ν*_max_: 1611, 1449, 1224, 994, 841, 798, 749 cm^−1^. ^1^H-NMR (600 MHz, CDCl_3_) δ: 7.93–7.91 (m, 2H), 7.57–7.54 (m, 4H), 7.01 (m, 1H), 6.82 (m, 1H), 6.75 (s, 1H). ^13^C-NMR (150 MHz, CDCl_3_) δ: 183.8, 164.8, 161.0, 156.6, 135.6, 132.0, 131.4, 129.3, 126.6, 111.6, 110.0, 107.2, 106.2. MS (ESI) *m*/*z*: calcd. for C_15_H_10_O_3_ [M+H]^+^: 239.07, found 239.25.

#### 3.2.2. General Procedure for the Synthesis of Compounds **2a**, **2b**, and **2c**

To a DMF solution (1.4 mL) of 5-hydroxyflavone (100 mg, 0.42 mmol), anhydrous potassium carbonate (173 mg, 1.26 mmol) was added. The reaction mixture was stirred at room temperature for 10 min, followed by the addition of an appropriate dibromoalkane (1.26 mmol). The resulting solution was stirred for three days at room temperature, diluted with a mixture of ethyl acetate and diethyl ether (100 mL, 1:1, *v*/*v*), and rinsed with water (10 mL) and brine (10 mL × 4). The organic layer was dried with anhydrous sodium sulfate and concentrated under vacuum. Flash column chromatography of the residue [silica gel, ethyl acetate/hexane (80/20, *v*/*v*)] yielded **2a**, **2b**, or **2c** (25–53%) as a white solid.

Compound **2a**. White solid (36 mg, 25%). IR (film) *ν*_max_: 3054, 2986, 2306, 1645, 1606, 1421 cm^−1^. ^1^H-NMR (600 MHz, CDCl_3_) δ: 7.97 (d, *J* = 7.7 Hz, 2H), 7.69 (m, 1H), 7.55 (m, 3H), 7.37 (m, 1H), 7.28 (d, *J* = 8.7 Hz, 1H), 6.92 (d, *J* = 8.3 Hz, 1H), 4.44 (t, *J* = 6.4 Hz, 2H), 3.77 (t, *J* = 6.9 Hz, 2H). ^13^C-NMR (150 MHz, CDCl_3_) δ: 178.8, 163.8, 158.5, 158.3, 135.2, 132.6, 130.7, 129.4, 126.8, 111.4, 109.8, 107.5, 107.4, 70.0, 28.2. HRMS (DART) *m*/*z*: calcd. for C_17_H_14_BrO_3_ [M+H]^+^: 345.0121, found 345.0132.

Compound **2b**. White solid (64 mg, 47%). IR (film) *ν*_max_: 3054, 2987, 2307, 1423 cm^−1^. ^1^H-NMR (600 MHz, CDCl_3_) δ: 7.91 (d, *J* = 6.8 Hz, 2H), 7.60 (m, 1H), 7.51 (m, 3H), 7.17 (d, *J* = 8.9 Hz, 1H), 6.91 (m, 1H), 6.86 (d, *J* = 7.8 Hz, 1H), 4.26 (t, *J* = 5.7 Hz, 2H), 3.85 (t, *J* = 6.1 Hz, 2H), 2.44 (m, 2H). ^13^C-NMR (150 MHz, CDCl_3_) δ: 178.8, 163.3, 159.2, 158.5, 135.1, 132.4, 130.8, 129.3, 126.7, 113.6, 110.4, 108.3, 107.6, 66.8, 32.1, 30.7. HRMS (DART) *m*/*z*: calcd. for C_18_H_16_BrO_3_ [M+H]^+^: 359.0277, found 359.0203.

Compound **2c**. White solid (53 mg, 53%). IR (film) *ν*_max_: 2938, 1659, 1478, 1450 cm^−1^. ^1^H-NMR (600 MHz, CDCl_3_) δ: 7.91 (dd, *J* = 6.7, 1.3 Hz, 2H), 7.57 (t, *J* = 8.4, 1H), 7.50 (m, 3H), 7.15 (d, *J* = 8.4 Hz, 1H), 6.91 (s, 1H), 6.82 (d, *J* = 8.2 Hz, 1H), 4.15 (t, *J* = 6.1 Hz, 2H), 3.58 (t, *J* = 6.3 Hz, 2H), 2.23 (m, 2H), 2.09 (m, 2H). ^13^C-NMR (150 MHz, CDCl_3_) δ: 178.5, 162.0, 159.2, 158.4, 134.3, 131.8, 131.3, 129.1, 126.4, 114.3, 110.2, 108.5, 107.9, 68.5, 34.2, 29.4, 27.6. HRMS (DART) *m*/*z*: calcd. for C_19_H_18_BrO_3_ [M+H]^+^: 373.0434, found 373.0399.

#### 3.2.3. General Procedure for the Synthesis of Compounds **3a**-**8c**

To a DMF solution (5 mL) of an appropriate bromo-containing compound (**2a**, **2b**, or **2c**, 0.15 mmol), anhydrous potassium carbonate (0.45 mmol) was added. The reaction mixture was stirred for 10 min, followed by the addition of an appropriate secondary amine (0.45 mmol). The reaction solution was stirred for 24 h at room temperature, diluted with a mixture of ethyl acetate and diethyl ether (100 mL, 1:1, *v*/*v*), and rinsed with water (10 mL) and brine (10 mL × 4). The organic layer was dried over anhydrous sodium sulfate and concentrated under vacuum. Flash column chromatography of the residue [silica gel, CH_2_Cl_2_/methanol/NH_4_OH (90/10/1, *v*/*v*/*v*)] yielded **3b**, **3c**, **4b**, **4c**, **5a**, **6a**, **6b**, **6c**, **7c**, **8b**, or **8c**. Several compounds (**3a**, **4a**, **5b**, **5c**, **7a**, **7b**, and **8a**) were purified using preparative TLC (pTLC) purification with a mixture of CH_2_Cl_2_/methanol (90/10, *v*/*v*) as eluent. The resulting compound was retrieved from the pTLC silica through washing with dichloromethane/diethylamine (100/3, *v*/*v*) and concentrated under vacuum.

Compound **3c**. White solid (27 mg, 50%). IR (film) *ν*_max_: 2957, 1650, 1603, 1479, 1461 cm^−1^. ^1^H-NMR (600 MHz, CDCl_3_) δ: 7.95 (d, *J* = 7.7 Hz, 2H), 7.67 (t, *J* = 7.7 Hz, 1H), 7.56 (overlapped, 4H), 7.22 (s, 1H), 6.85 (d, *J* = 8.1 Hz, 1H), 4.16 (m, 2H), 3.76 (m, 2H), 3.43 (m, 2H), 2.98 (m, 2H), 2.26 (m, 4H), 2.09 (m, 4H). ^13^C-NMR (150 MHz, CDCl_3_) δ: 178.2, 161.4, 158.8, 158.4, 134.0, 131.54, 131.50, 129.1, 126.2, 114.7, 110.5, 109.0, 107.6, 69.2, 55.6, 53.7, 26.2, 24.0, 23.6. HRMS (DART) *m*/*z*: calcd. for C_23_H_26_NO_3_ [M+H]^+^: 364.1907, found 364.1908.

Compound **4c**. Light-yellow solid (39 mg, 81%). IR (film) *ν*_max_: 2933, 1647, 1601, 1479, 1459 cm^−1^_._^1^H-NMR (600 MHz, CDCl_3_) δ: 7.88 (dd, *J* = 7.1, 1.7 Hz, 2H), 7.53 (t, 8.3 Hz, 1H), 7.49 (m, 3H), 7.10 (dd, *J* = 8.4, 0.8 Hz, 1H), 6.80 (d, *J* = 7.9 Hz, 1H), 6.68 (s, 1H), 4.12 (t, *J* = 6.2 Hz, 2H), 2.69–2.41 (overlapped, 6H), 1.96 (m, 2H), 1.87 (m, 2H), 1.67 (m, 4H), 1.46 (m, 2H). ^13^C-NMR (150 MHz, CDCl_3_) δ: 178.2, 161.2, 159.0, 158.4, 133.9, 131.6, 131.5, 129.1, 126.2, 114.8, 110.3, 109.1, 107.7, 69.2, 58.1, 53.8, 26.7, 24.2, 23.3, 22.2. HRMS (DART) *m*/*z*: calcd. for C_24_H_28_NO_3_ [M+H]^+^: 378.2064, found 378.2038.

Compound **5c**. White solid (21 mg, 42%). IR (film) *ν*_max_: 3054, 2986, 1636, 1423 cm^−1^. ^1^H-NMR (600 MHz, CDCl_3_) δ: 7.89 (m, 2H), 7.57–7.48 (overlapped, 4H), 7.12 (d, *J* = 8.4 Hz, 1H), 6.80 (d, *J* = 8.4 Hz, 1H), 6.69 (s, 1H), 4.14 (t, *J* = 6.0 Hz, 2H), 3.80 (m, 4H), 2.81–2.48 (overlapped, 6H), 1.98 (m, 2H), 1.90 (m, 2H). ^13^C-NMR (150 MHz, CDCl_3_) δ: 178.2, 161.1, 159.2, 158.4, 133.8, 131.6, 131.4, 129.0, 126.1, 114.8, 110.1, 109.1, 107.6, 69.2, 66.4, 58.4, 53.4, 26.8, 22.6. HRMS (DART) *m*/*z*: calcd. for C_23_H_26_NO_4_ [M+H]^+^: 380.1856, found 380.1804.

Compound **6c**. White solid (16 mg, 30%). IR (film) *ν*_max_: 3053, 2987, 1645, 1422 cm^−1^. ^1^H-NMR (600 MHz, CDCl_3_) δ: 7.89 (dd, *J* = 7.5, 2.0 Hz, 2H), 7.54 (t, *J* = 8.3 Hz, 1H), 7.51 (m, 3H), 7.12 (d, *J* = 8.3 Hz, 1H), 6.79 (d, *J* = 8.4 Hz, 1H), 6.67 (s, 1H), 4.13 (t, *J* = 5.7 Hz, 2H), 3.24–2.47 (overlapped, 10H), 2.41 (s, 3H), 1.97 (m, 4H). ^13^C-NMR (150 MHz, CDCl_3_) δ: 178.2, 161.3, 158.9, 158.4, 133.9, 131.52, 131.50, 129.1, 126.2, 114.8, 110.5, 109.1, 107.6, 69.0, 57.0, 52.4, 50.8, 44.6, 26.3, 22.2. HRMS (DART) *m*/*z*: calcd. for C_24_H_29_N_2_O_3_ [M+H]^+^: 393.2173, found 393.2134.

Compound **7c**. Light-yellow solid (24 mg, 48%). IR (film) *ν*_max_: 3054, 2987, 1645, 1422 cm^−1^. ^1^H-NMR (600 MHz, CDCl_3_) δ: 7.89 (dd, *J* = 7.0, 1.5 Hz, 2H), 7.55 (t, *J* = 8.0 Hz, 1H), 7.53 (m, 3H), 7.12 (d, *J* = 8.5 Hz, 1H), 6.81 (d, *J* = 8.0 Hz, 1H), 6.67 (s, 1H), 4.14 (t, *J* = 6.1 Hz, 2H), 2.94–2.57 (overlapped, 6H), 1.98 (m, 2H), 1.91 (m, 2H), 1.16 (t, *J* = 6.1 Hz, 6H). ^13^C-NMR (150 MHz, CDCl_3_) δ: 178.2, 161.1, 159.1, 158.4, 133.8, 131.6, 131.4, 129.1, 126.2, 114.8, 110.2, 109.1, 107.7, 69.3, 52.2, 46.8, 26.9, 22.6, 10.7. HRMS (DART) *m*/*z*: calcd. for C_23_H_28_NO_3_ [M+H]^+^: 366.2064, found 366.2096.

Compound **8c**. Light-yellow solid (48 mg, 92%). IR (film) *ν*_max_: 3054, 2987, 1646, 1422 cm^−1^. ^1^H-NMR (600 MHz, CD_3_OD) δ: 7.98 (dd, *J* = 7.3, 1.5 Hz, 2H), 7.66 (t, *J* = 9.0 Hz, 1H), 7.56 (m, 3H), 7.22 (d, *J* = 8.5 Hz, 1H), 6.98 (d, *J* = 8.3 Hz, 1H), 6.74 (s, 1H), 4.16 (t, *J* = 5.9 Hz, 2H), 2.58 (t, *J* = 7.6 Hz, 2H), 2.45 (t, *J* = 7.8 Hz, 4H), 1.88 (m, 2H), 1.75 (m, 2H), 1.48 (m, 4H), 0.88 (t, *J* = 7.3 Hz, 6H). ^13^C-NMR (150 MHz, CD_3_OD) δ: 179.1, 162.1, 159.1, 158.4, 134.5, 131.5, 131.1, 128.9, 126.0, 113.9, 109.7, 107.9, 107.8, 68.9, 55.7, 53.2, 26.6, 22.3, 19.2, 10.9. HRMS (DART) *m*/*z*: calcd. for C_25_H_32_NO_3_ [M+H]^+^: 394.2377, found 394.2305.

Compound **3b**. Light-tan solid (39 mg, 81%). IR (film) *ν*_max_: 3054, 2987, 1635, 1422 cm^−1^. ^1^H-NMR (600 MHz, CDCl_3_) δ: 7.89 (dd, *J* = 8.1, 1.4 Hz, 2H), 7.60 (t, *J* = 8.3 Hz, 1H), 7.54 (m, 3H), 7.19 (d, *J* = 8.5 Hz, 1H), 6.83 (d, *J* = 8.1 Hz, 1H), 6.66 (s, 1H), 4.25 (t, *J* = 5.6 Hz, 2H), 3.77–3.16 (overlapped, 6H), 2.49 (m, 2H), 2.19 (t, *J* = 6.6 Hz, 4H). ^13^C-NMR (150 MHz, CDCl_3_) δ: 178.6, 161.8, 158.4, 158.3, 134.2, 131.7, 131.4, 129.1, 126.2, 114.5, 110.8, 108.9, 107.8, 67.3, 54.3, 53.6, 26.5, 23.6. HRMS (DART) *m*/*z*: calcd. for C_22_H_24_NO_3_ [M+H]^+^: 350.1751, found 350.1724.

Compound **4b**. Light-tan solid (27 mg, 54%). IR (film) *ν*_max_: 3055, 2987, 1653, 1636, 1422 cm^−1^. ^1^H-NMR (600 MHz, CDCl_3_) δ: 7.86 (dd, *J* = 7.5, 2.0 Hz, 2H), 7.54 (t, *J* = 8.3 Hz, 1H), 7.51 (m, 3H), 7.11 (d, *J* = 8.4 Hz, 1H), 6.80 (d, *J* = 8.4 Hz, 1H), 6.65 (s, 1H), 4.18 (t, *J* = 6.0 Hz, 2H), 3.14–2.76 (overlapped, 6H), 2.33 (m, 2H), 1.83 (m, 4H), 1.56 (m, 2H). ^13^C-NMR (150 MHz, CDCl_3_) δ: 178.3, 161.2, 159.1, 158.4, 133.8, 131.6, 131.4, 129.0, 126.2, 114.8, 110.2, 109.2, 107.9, 67.9, 55.8, 54.5, 26.1, 25.4, 24.0. HRMS (DART) *m*/*z*: calcd. for C_23_H_26_NO_3_ [M+H]^+^: 364.1907, found 364.1860.

Compound **5b**. White solid (33 mg, 65%). IR (film) *ν*_max_: 3054, 2988, 2362, 1653, 1423 cm^−1^. ^1^H-NMR (600 MHz, CD_3_OD) δ: 7.97 (dd, *J* = 8.1, 1.6 Hz, 2H), 7.66 (t, *J* = 8.3 Hz, 1H), 7.55 (m, 3H), 7.22 (d, *J* = 8.8 Hz, 1H), 6.98 (d, *J* = 8.2 Hz, 1H), 6.73 (s, 1H), 4.18 (t, *J* = 6.0 Hz, 2H), 3.70 (t, *J* = 4.7 Hz, 4H), 2.67 (t, *J* = 7.1 Hz, 2H), 2.53 (m, 4H), 2.08 (m, 2H). ^13^C-NMR (150 MHz, CD_3_OD) δ: 179.2, 162.2, 159.0, 158.4, 134.6, 131.6, 131.1, 128.9, 126.0, 113.9, 109.8, 107.9, 107.8, 67.3, 66.3, 55.3, 53.5, 25.3. HRMS (DART) *m*/*z*: calcd. for C_22_H_24_NO_4_ [M+H]^+^: 366.1700, found 366.1674.

Compound **6b**. Light-yellow solid (25 mg, 48%). IR (film) *ν*_max_: 3055, 2987, 1653, 1605, 1457, 1421 cm^−1^. ^1^H-NMR (600 MHz, CD_3_OD) δ: 7.96 (dd, *J* = 7.6, 1.2 Hz, 2H), 7.65 (t, *J* = 8.3 Hz, 1H), 7.55 (m, 3H), 7.22 (d, *J* = 8.4 Hz, 1H), 6.97 (d, *J* = 8.3 Hz, 1H), 6.73 (s, 1H), 4.17 (t, *J* = 6.1 Hz, 2H), 3.18–2.33 (overlapped, 10H), 2.29 (s, 3H), 2.10 (m, 2H). ^13^C-NMR (150 MHz, CD_3_OD) δ: 179.1, 162.3, 158.8, 158.3, 134.6, 131.6, 131.0, 128.9, 126.0, 113.8, 110.0, 107.9, 107.8, 67.4, 53.7, 52.3, 48.1, 44.5, 25.2. HRMS (DART) *m*/*z*: calcd. for C_23_H_27_N_2_O_3_ [M+H]^+^: 379.2016, found 379.2033.

Compound **7b**. Tan solid (28 mg, 58%). IR (film) *ν*_max_: 3056, 2987, 1700, 1653, 1635, 1558, 1457 cm^−1^. ^1^H-NMR (600 MHz, CD_3_OD) δ: 7.98 (dd, *J* = 7.4, 1.8 Hz, 2H), 7.67 (t, *J* = 8.4 Hz, 1H), 7.55 (m, 3H), 7.21 (d, *J* = 8.4 Hz, 1H), 6.98 (d, *J* = 8.4 Hz, 1H), 6.74 (s, 1H), 4.17 (t, *J* = 6 Hz, 2H), 2.81 (t, *J* = 7.8 Hz, 2H), 2.61 (q, *J* = 7.2 Hz, 4H), 2.04 (m, 2H), 1.09 (t, *J* = 7.2 Hz, 6H). ^13^C-NMR (150 MHz, CD_3_OD) δ: 179.1, 162.2, 159.0, 158.4, 134.5, 131.5, 131.1, 128.9, 126.0, 113.9, 109.8, 107.8, 107.4, 67.4, 49.0, 46.6, 25.2, 10.1. HRMS (DART) *m*/*z*: calcd. for C_22_H_26_NO_3_ [M+H]^+^: 352.1907, found 352.1862.

Compound **8b**. White solid (25 mg, 48%). IR (film) *ν*_max_: 3054, 2991, 1652, 1423 cm^−1^. ^1^H-NMR (600 MHz, CD_3_OD) δ: 8.02 (dd, *J* = 7.6, 1.8 Hz, 2H), 7.75 (*t, J* = 8.4 Hz, 1H), 7.58 (m, 3H), 7.34 (d, *J* = 8.5 Hz, 1H), 7.05 (d, *J* = 8.2 Hz, 1H), 6.85 (s, 1H), 4.33 (t, *J* = 9.2 Hz, 2H), 3.30 (m, 2H), 3.12 (m, 4H), 2.29 (m, 2H), 1.78 (m, 4H), 0.98 (t, *J* = 7.4 Hz, 6H). ^13^C-NMR (150 MHz, CD_3_OD) δ: 179.4, 163.0, 158.2, 157.7, 135.0, 131.8, 130.9, 129.0, 126.1, 113.3, 110.8, 107.8, 107.5, 68.5, 55.7, 51.9, 23.5, 17.1, 10.2. HRMS (DART) *m*/*z*: calcd. for C_24_H_30_NO_3_ [M+H]^+^: 380.2220, found 380.2213.

Compound **3a**. Light-yellow solid (32 mg, 66%). IR (film) *ν*_max_: 3056, 2987, 1634, 1423 cm^−1^. ^1^H-NMR (600 MHz, CD_3_OD) δ: 8.00 (dd, *J* = 8.0, 1.5 Hz, 2H), 7.71 (t, *J* = 8.4 Hz, 1H), 7.55 (m, 3H), 7.30 (d, *J* = 8.3 Hz, 1H), 7.04 (d, *J* = 8.1 Hz, 1H), 6.78 (s, 1H), 4.32 (t, *J* = 5.5 Hz, 2H), 3.18 (t, *J* = 5.6 Hz, 2H), 2.91 (t, *J* = 5.7, 4H), 1.89 (m, 4H). ^13^C-NMR (150 MHz, CD_3_OD) δ: 179.2, 162.5, 158.40, 158.36, 134.7, 131.7, 131.0, 128.9, 126.0, 114.1, 110.5, 108.5, 107.8, 67.8, 54.4, 53.9, 22.9. HRMS (DART) *m*/*z*: calcd for C_21_H_22_NO_3_ [M+H]^+^: 336.1594, found 336.1614.

Compound **4a**. Light-yellow solid (24 mg, 49%). IR (film) *ν*_max_: 3055, 2987, 1507, 1423 cm^−1^. ^1^H-NMR (600 MHz, CD_3_OD) δ: 7.98 (m, 2H), 7.68 (t, *J* = 8.4 Hz, 1H), 7.53 (m, 3H), 7.23 (d, *J* = 8.4 Hz, 1H), 6.98 (d, *J* = 8.4 Hz, 1H), 6.74 (s, 1H), 4.28 (t, *J* = 6.0 Hz, 2H), 2.95 (t, *J* = 6 Hz, 2H), 2.69 (m, 4H), 1.63 (m, 4H), 1.48 (m, 2H). ^13^C-NMR (150 MHz, CD_3_OD) δ: 179.0, 162.3, 158.6, 158.4, 134.6, 131.6, 131.1, 128.9, 126.0, 114.0, 110.2, 108.2, 107.8, 67.2, 56.9, 54.8, 25.2, 23.6. HRMS (DART) *m*/*z*: calcd. for C_22_H_24_NO_3_ [M+H]^+^: 350.1751, found 350.1752.

Compound **5a**. White solid (35 mg, 68%). IR (film) *ν*_max_: 3055, 2986, 1700, 1653, 1559, 1420 cm^−1^. ^1^H-NMR (600 MHz, CD_3_OD) δ: 7.98 (m, 2H), 7.68 (t, *J* = 8.4 Hz, 1H), 7.55 (m, 3H), 7.25 (dd, *J* = 8.5, 0.9 Hz, 1H), 7.00 (dd, *J* = 8.3, 0.7 Hz, 1H), 6.75 (s, 1H), 4.29 (t, *J* = 5.4 Hz, 2H), 3.72 (t, *J* = 4.7 Hz, 4H), 2.94 (t, *J* = 5.5 Hz, 2H), 2.73 (t, *J* = 4.3 Hz, 4H). ^13^C-NMR (150 MHz, CD_3_OD) δ: 179.0, 162.3, 158.6, 158.4, 134.6, 131.6, 131.1, 128.9, 126.0, 114.0, 110.2, 108.1, 107.8, 67.7, 66.4, 56.8, 54.1. HRMS (ESI) *m*/*z*: calcd. for C_21_H_22_NO_4_ [M+H]^+^: 352.1543, found 352.1553.

Compound **6a**. White solid (43 mg, 21%). IR (film) *ν*_max_: 3053, 2985, 1643, 1558, 1507 cm^−1^. ^1^H-NMR (600 MHz, CD_3_OD) δ: 7.96 (m, 2H), 7.65 (t, *J* = 8.4 Hz, 1H), 7.54 (m, 3H), 7.23 (dd, *J* = 8.4, 1.0 Hz, 1H), 6.98 (dd, *J* = 8.3, 0.7 Hz, 1H), 6.71 (s, 1H), 4.25 (t, *J* = 5.6 Hz, 2H), 2.94 (t, *J* = 5.6 Hz, 2H), 2.90–2.30 (overlapped, 8H), 2.28 (s, 3H). ^13^C-NMR (150 MHz, CD_3_OD) δ: 179.0, 162.2, 158.6, 158.3, 134.5, 131.5, 131.1, 128.9, 126.0, 114.0, 110.1, 108.0, 107.8, 67.6, 56.2, 54.3, 53.1, 44.6. HRMS (ESI) *m*/*z*: calcd. for C_22_H_25_N_2_O_3_ [M+H]^+^: 365.1860, found 365.1874.

Compound **7a**. Light-yellow solid (17 mg, 38%). IR (film) *ν*_max_: 3053, 2987, 1700, 1653, 1635 cm^−1^. ^1^H-NMR (600 MHz, CD_3_OD) δ: 8.03 (dd, *J* = 8.2, 2.0 Hz, 2H), 7.79 (t, *J* = 8.3 Hz, 1H), 7.59 (m, 3H), 7.44 (d, *J* = 8.5 Hz, 1H), 7.15 (d, *J* = 8.2 Hz, 1H), 6.88 (s, 1H), 4.53 (t, *J* = 5.1 Hz, 2H), 3.66 (t, *J* = 5.2 Hz, 2H), 3.42 (m, 4H), 1.40 (t, *J* = 7.2 Hz, 6H). ^13^C-NMR (150 MHz, CD_3_OD) δ: 179.1, 162.3, 158.7, 158.4, 134.6, 131.6, 131.1, 128.9, 126.0, 114.0, 110.2, 108.2, 107.8, 67.5, 50.9, 47.4, 10.2. HRMS (DART) *m*/*z*: calcd. for C_21_H_24_NO_3_ [M+H]^+^: 338.1751, found 338.1752.

Compound **8a**. Light-yellow solid (18 mg, 34%). IR (film) *ν*_max_: 3057, 2987, 1653, 1636, 1457 cm^−1^. ^1^H-NMR (600 MHz, CD_3_OD) δ: 8.01 (dd, *J* = 7.4, 1.4 Hz, 2H), 7.70 (t, *J* = 8.4 Hz, 1H), 7.57 (m, 3H), 7.29 (d, J = 8.3 Hz, 1H), 7.04 (d, *J* = 8.5 Hz, 1H), 6.79 (s, 1H), 4.27 (t, *J* = 5.6 Hz, 2H), 3.13 (m, 2H), 2.69 (m, 4H), 1.59 (m, 4H), 0.92 (t, *J* = 7.5 Hz, 6H). ^13^C-NMR (150 MHz, CD_3_OD) δ: 179.2, 162.5, 158.5, 158.4, 134.6, 131.6, 131.1, 128.9, 126.0, 114.1, 100.4, 108.5, 107.8, 67.3, 56.4, 52.2, 19.2, 10.7. HRMS (DART) *m*/*z*: calcd. for C_23_H_28_NO_3_ [M+H]^+^: 366.2064, found 366.2116.

#### 3.2.4. Synthesis of Compound **9b**

5-Hydroxyflavone (208 mg, 0.873 mmol) was dissolved in DMF (5 mL) and stirred for 10 min, followed by the addition of anhydrous potassium carbonate (362 mg, 2.62 mmol). The reaction mixture was stirred for 10 min. 1-Bromo-4-methylpentane (436 mg, 2.7 mmol) was added and stirred at 80 °C overnight under nitrogen. The reaction mixture was diluted in a mixture of ethyl acetate and diethyl ether (100 mL, 1:1, *v*/*v*) and rinsed with water (10 mL) and brine (10 mL × 4). The organic layer was dried over anhydrous sodium sulfate and concentrated under vacuum. Flash column chromatography of the residue [silica gel, ethyl acetate/hexane (80/20, *v*/*v*)] yielded compound **9b** (35 mg, 12%) as a white solid. IR (film) *ν*_max_: 3054, 2988, 1652 cm^−1^. ^1^H-NMR (600 MHz, CD_3_OD) δ: 7.98 (dd, *J* = 7.8, 1.8 Hz, 2H), 7.66 (t, *J* = 8.3 Hz, 1H), 7.54 (m, 3H), 7.22 (d, *J* = 8.4 Hz, 1H), 6.97 (d, *J* = 8.4 Hz, 1H), 6.74 (s, 1H), 4.11 (t, *J* = 6.6 Hz, 2H), 1.88 (m, 2H), 1.63 (m, 1H), 1.42 (m, 2H), 0.94 (d, *J* = 6.5 Hz, 6H). ^13^C-NMR (150 MHz, CD_3_OD) δ: 179.3, 162.2, 159.2, 158.4, 134.5, 131.5, 131.1, 128.9, 126.0, 113.9, 109.6, 107.9, 107.7, 69.5, 34.9, 27.7, 26.5, 21.7. HRMS (ESI) *m*/*z*: calcd. for C_21_H_23_O_3_ [M+H]^+^: 323.1642, found 323.1643.

#### 3.2.5. Synthesis of Compound **11b**

To a DMF solution (5 mL) of 5-hydroxyflavone (250 mg, 1.05 mmol), anhydrous potassium carbonate (0.870 g, 6.295 mmol) was added. The reaction mixture was stirred for 10 min, followed by the addition of 3-bromo-1-propanol (854 μL, 9.44 mmol), and then further stirred at room temperature for 2 days. The reaction was monitored using TLC (R_f_ = 0.30, hexane/ethyl acetate = 30:70 *v*/*v*, silica gel 60 GF_254_) by UV. The reaction was partitioned between ethyl acetate (50 mL) and water (5 mL). The organic layer was further washed with water (5 mL) and a saturated NaCl solution (5 mL), dried over anhydrous sodium sulfate, and concentrated under vacuum. Flash column chromatography of the residue yielded compound **11b** (175 mg, 56%) as a white solid. IR (KBr): 1635, 1460, 1382, 1270, 1074, 755 cm^−1^. ^1^H-NMR (600 MHz, CDCl_3_): δ 7.85–7.83 (m, 1H), 7.56–7.55 (m, 1H), 7.53–7.46 (m, 3H), 7.10 (d, *J* = 8.4 Hz, 1H), 6.77 (d, *J* = 8.4 Hz, 1H), 6.70 (s, 1H), 5.03 (broad, 1H), 4.22 (t, *J* = 5.7 Hz, 2H), 3.93 (t, *J* = 4.8 Hz, 2H), 2.17–2.14 (m, 2H). ^13^C-NMR (150 MHz, CDCl_3_): δ 178.5, 161.7, 158.6, 158.1, 134.2, 131.6, 131.2, 129.1, 126.2, 114.1, 110.3, 108.8, 106.9, 69.7, 61.9, 32.0. HRMS (ESI) *m*/*z*: calcd. for C_18_H_16_O_4_ [M+H]^+^: 297.1121, found 297.1133.

#### 3.2.6. Synthesis of Compound **12**

To a DMF solution (10 mL) of **2b** (115 mg, 0.320 mmol), sodium azide (62.4 mg, 0.960 mmol) was added. The reaction mixture was stirred at room temperature for 64 h. The reaction was monitored using TLC (R_f_ = 0.24, hexane/ethyl acetate = 80:20 *v*/*v*, silica gel 60 GF_254_) by UV. The reaction mixture was partitioned between ethyl acetate (45 mL) and water (5 mL). The organic layer was washed with water (5 mL × 3), dried over anhydrous sodium sulfate, and concentrated under vacuum. Flash column chromatography of the residue yielded compound **12** as an off-white solid (98 mg, 95%). IR (KBr): 2096, 1644, 1604, 1460, 1377, 1269, 1099, 752 cm^−1^. ^1^H-NMR (600 MHz, CDCl_3_): δ 7.86 (d, *J* = 7.2 Hz, 2H), 7.54–7.47 (m, 4H), 7.11 (d, *J* = 8.4 Hz, 1H), 6.78 (d, *J* = 8.4 Hz, 1H), 6.75 (s, 1H), 4.16 (t, *J* = 6.0 Hz, 2H), 3.74 (t, *J* = 6.5 Hz, 2H), 2.15 (quint, *J* = 6.0 Hz, 2H). ^13^C-NMR (150 MHz, CDCl_3_): δ 178.2, 161.5, 158.9, 158.3, 134.0, 131.6, 131.4, 129.1, 126.2, 114.7, 110.5, 108.8, 107.9, 65.9, 48.1, 28.8. HRMS (ESI) *m*/*z*: calcd. for C_18_H_15_N_3_O_3_ [M+H]^+^: 322.1186, found 322.1216.

#### 3.2.7. Synthesis of Compound **10b**

To a DMF solution (10 mL) of **12** (82.0 mg, 0.255 mmol), triphenyl phosphine (334 mg, 1.28 mmol) was added. The reaction mixture was stirred at room temperature for 34 h, followed by the addition of water (1 mL), and was further stirred for 67 h. The reaction was monitored using TLC (R_f_ = 0.15, CH_2_Cl_2_/MeOH/NH_4_OH = 90:10:1 *v*/*v*/*v*, silica gel 60 GF_254_) by UV. The reaction was partitioned between CH_2_Cl_2_ (45 mL) and water. The organic layer was washed with brine (5 mL × 2), dried over anhydrous sodium sulfate, and concentrated under vacuum. Compound **10b** was purified using preparative TLC (pTLC) purification with a mixture of CH_2_Cl_2_/MeOH/NH_4_OH (90:10:1 *v*/*v*/*v*) as eluent. The resulting compound was retrieved from the pTLC silica through washing with a mixture of CH_2_Cl_2_/MeOH/NH_4_OH (90:10:1 *v*/*v*/*v*) and concentrated under vacuum (off-white solid, 44 mg, 58%). IR (KBr): 1638, 1600, 1382, 1094, 753 cm^−1^. ^1^H-NMR (600 MHz, CD_3_OD): δ 7.95 (m, 2H), 7.72 (t, *J* = 8.4 Hz, 1H), 7.57–7.52 (m, 3H), 7.28 (d, *J* = 8.4 Hz, 1H), 7.01 (d, *J* = 8.4 Hz, 1H), 6.74 (s, 1H), 4.30 (t, *J* = 5.4 Hz, 2H), 3.30–3.28 (m, 2H), 2.28–2.26 (m, 2H). ^13^C-NMR (150 MHz, CD_3_OD): δ 179.1, 162.8, 158.1, 157.6, 134.9, 131.8, 130.8, 128.9, 126.1, 113.3, 110.8, 107.6, 107.3, 69.0, 38.8, 25.9. HRMS (ESI) *m*/*z*: calcd. for C_18_H_17_NO_3_ [M+H]^+^: 296.1281, found 296.1301.

### 3.3. Thermal Denaturation of poly(dA)•2poly(dT) Monitored by UV

A solution (1 mL) of poly(dA)•poly(dT) (15 μM/base pair) and poly(dT) (15 μM/base) in sodium cacodylate buffer (10 mM, pH 7.0) and KCl (150 mM) in the presence and absence of a ligand at the desired concentration was heated at 95 °C for 5 min, slowly cooled to 25 °C, and incubated at 4 °C overnight. The UV melting spectra of samples were recorded in 1 cm path-length quartz cuvettes at 260 nm, 280 nm, and 284 nm as a function of temperature (25–90 °C, heating rate: 0.2 °C/min). Melting temperatures (T_m_s) were determined using the first derivative method. All experiments were carried out in duplicate.

### 3.4. Thermal Denaturation of poly(dA)•2poly(dT) Monitored by UV Under Salt-Deficient Conditions

A solution (1 mL) of poly(dA)•poly(dT) (15 μM/base pair) and poly(dT) (15 μM/base) in a lithium cacodylate buffer (10 mM, pH 7.0) in the presence and absence of a ligand (**4a**, **4b**, or **4c**, 10 μM) was heated at 95 °C for 5 min, slowly cooled to 25 °C, and incubated at 4 °C overnight. The UV melting spectra of samples were recorded in 1 cm path-length quartz cuvettes at 260 nm, 280 nm, and 284 nm as a function of temperature (25–90 °C, heating rate: 0.2 °C/min). Melting temperatures (T_m_s) were determined using the first derivative method. All experiments were carried out in duplicate.

### 3.5. Viscosity Experiments

The poly(dA)●2poly(dT) sample (2 mL) was prepared by mixing poly(dA)●poly(dT) (500 μM/base triplet) and poly(dT) (500 μM/base triplet) in a sodium cacodylate buffer (10 mM) and KCl (150 mM) at a pH of 7.0. The DNA solution was heated at 95 °C for 5 min, slowly cooled to 25 °C, and incubated at 4 °C overnight to form the corresponding triplex. Calf thymus DNA (1 mM, 2 mL) was prepared in a sodium cacodylate buffer (10 mM) and KCl (150 mM) at a pH of 7.0. Small aliquots of a concentrated stock solution of the desired ligand (**4b** for triplex and ethidium bromide for duplex) were added to the poly(dA)●2poly(dT) sample (500 μM) or calf thymus DNA (1 mM) in an Ostwald viscometer at room temperature to obtain the desired ligand–DNA ratios. After each addition of ligand, the flow time, the time taken for the level of the solution to pass between the two marks on the viscometer, was recorded using a stopwatch. The measurements were triplicated for each ligand–DNA ratio. The relative viscosities were calculated based on published equations [34].

### 3.6. Visualization of Triplex Formation by Native PAGE

The triplex samples for native PAGE were prepared by mixing **DNA1** (3 pmol), **DNA2** (3 pmol), and **TFO1** (3 pmol) in 20 μL of a Tris–acetate buffer (90 mM, pH 5.0) in the absence and presence of ligand **4b** (1, 2.5, 5, 10, 20, 50, and 100 μM). The duplex sample for native PAGE was prepared by mixing **DNA1** (3 pmol) and **DNA2** (3 pmol) in 20 μL of a Tris–acetate buffer (90 mM, pH 5.0). The DNA (triplex and duplex) solutions were heated to 95 °C for 5 min, slowly cooled down to room temperature, and stored at 4 °C overnight. Samples of **DNA1** (3 pmol) only, **DNA2** (3 pmol) only, and **TFO1** (3 pmol) only were also prepared, as control samples. DNA separations were carried out using a 12% nondenaturing polyacrylamide gel made in a Tris–acetate buffer (90 mM, pH 5.0). DNA bands were visualized using the corresponding fluorescence channels for Cy3 and Cy5 on an Invitrogen iBright FL1500 imaging system and quantified by iBright analysis software version 5.3.0.

### 3.7. Preparation of DNA Solutions (DNA3 or DNA4) for ITC

Stock solutions of **DNA3** or **DNA4** were first dialyzed in 500 mL of a buffer (10 mM sodium cacodylate, 100 mM NaCl, pH 7.0) for 6 h and then further dialyzed in another fresh buffer (500 mL) overnight at room temperature. The concentration of **DNA3** or **DNA4** after dialysis was determined spectrophotometrically by UV. **DNA3** or **DNA4** (10 μM) was mixed in a 10 mM sodium cacodylate (pH 7.0) and 100 mM NaCl buffer, heated to 95 °C for 5 min, slowly cooled down to room temperature, and stored at 4 °C overnight.

### 3.8. Isothermal Titration Calorimetry (ITC)

In a typical experiment, 5 µL aliquots of a ligand (100 μM) were injected using a 264 µL rotating syringe into an isothermal sample cell containing 185 μL of **DNA3** or **DNA4** (10 µM) at 15 °C. The corresponding control experiment was carried out by injecting 5 µL aliquots of a ligand (100 μM) into a buffer solution alone. The syringe rotational speed was 125 rpm. The duration of each injection was 4 s, and the delay between injections was 300 s. An initial baseline was collected at 100 s before the first injection. A heat burst curve (microjoules per second vs. seconds) was generated from each injection. The area under each curve was calculated using the NanoAnalyzer software (Version 3.8.0) to yield a measurement of the heat associated with that injection. The heat of the ligand binding to DNA associated with that injection was obtained by subtracting the heat associated with each ligand–buffer injection from the corresponding heat associated with each ligand–DNA injection. The following buffer conditions were used in the ITC experiments: 10 mM sodium cacodylate buffer; 100 mM NaCl; pH 7.0.

### 3.9. Circular Dichroism Titration Measurements

Aliquots of **4b** (in DMSO) were gradually titrated into a solution (2 mL) of **DNA3** (5 μM) or **DNA4** (5 μM) in sodium cacodylate (10 mM, pH 7.0) and 100 mM NaCl at 15 °C. The final concentrations of **4b** were 0, 1, 2.5, 5, 10, 15, 20, 25, and 50 μM. The % DMSO in the solution was kept at no more than 1%. After each addition, the solution was mixed by gently swirling the close-capped cuvette. The interval time between each addition was 5 min for equilibrium. CD spectra were recorded from 500 to 230 nm with a 100 nm/min scan speed.

### 3.10. Plasmid Amplification

To a 50 μL aliquot of competent *E. coli* cells, 2 μL of plasmid (10 ng/μL) was added. The mixture was incubated on ice for 0.5 h before being subjected to a 42 °C heat shock for 30 s, and then it was put into an ice bath for 2 min. Lysogeny broth (LB, 200 μL) was added to the transformation vial, which was left to shake for 1 h at 37 °C and 200 rpm. The *E. coli* cells were plated on LB/amp plates and left to incubate at 37 °C overnight. The next day, colonies were selected and inoculated in a liquid culture for growth in the shaker at 37 °C overnight. The plasmid minipreps were performed using either the Zyppy Plasmid Miniprep Kit (Zymo Research, Irvine, CA, USA) or Qiagen Plasmid Midi Kit (Redwood City, CA, USA), following the manufacturer’s instructions. The DNA concentrations were measured at 260 nm using the Nano Drop 200c (ThermoFisher Scientific, Waltham, MA, USA).

### 3.11. Inhibition of DraI Activity by Ligand-Mediated Triplex Formation

Samples were prepared by mixing 300 ng of plasmid, **TFO2** (10 μM), *DraI* (20 units), and NaCl (50 mM) in the rCutSmart Buffer (New England Biolabs, USA) in the absence and presence of ligand **4b** (10, 25, 50, 75, and 100 μM). Control samples of the plasmid without *DraI*, the plasmid with *DraI* only, and the plasmid and **TFO2** were also prepared. Samples were incubated in a 37 °C water bath for 2 h before they were incubated at 65 °C for 20 min to inactivate *DraI*. The resulting DNA was resolved on a 1% agarose gel and visualized by staining with ethidium bromide using a BioRad ChemiDoc^TM^ XRS+ imaging system. The quantification of DNA fragments was carried out using Image Lab analysis software version 6.1.0.

### 3.12. Cell Culture Protocols for Cancer Cell Lines

Three mammalian cell lines were chosen from the NCI-60 panel for cytotoxicity studies: HL-60, HT-29, and HCT116. All three cell lines were maintained in RPMI 1640 (Gibco, Waltham, MA, USA) supplemented with 10% fetal bovine serum (HyClone, Logan, UT, USA), and 100 units/mL penicillin–streptomycin (Thermo Fisher), and incubated at 37 °C in a humidified 5% CO_2_ incubator. Typically, media renewal occurred every 2–3 days, and cells were passaged every 5–6 days.

### 3.13. Cytotoxicity Studies by the MTT Assay

All the experiments were carried out in triplicate. Cells were plated at the following densities—HL-60: 2 × 10^5^ cells/mL; HT-29: 1 × 10^5^ cells/mL; and HCT116: 1 × 10^5^ cells/mL—in 96-well plates and left to recover in the 37 °C incubator overnight. Cells were then treated with the desired ligand at various concentrations (0, 0.1, 0.5, 1, 2, 5, 10, 25, and 50 μM) and incubated at 37 °C for 24 h. After the addition of a solution of MTT dissolved in PBS (5 mg/mL) for a final concentration of 0.5 mg/mL per well, the cells were re-incubated at 37 °C for 4 h. The resulting plates were spun down in a centrifuge (526× *g*) for 5 min, the media was removed, and 100 μL of DMSO was added to dissolve the formazan crystals. The quantification of viable cells was carried out by measuring the absorbance at 570 nm using a microplate reader (Tecan Infinite M Plex, Männedorf, Switzerland).

Untreated cells and media with no cells were used as controls. Cell viability was calculated using the following equation:

$$Viability(\%)=\frac{\left(A_{treated}\right)-\left(A_{media}\right)}{\left(A_{untreated}\right)-\left(A_{media}\right)}\times 100\%$$

*A_treated_*—the absorbance of the solution containing treated cells; *A_media_*—the absorbance of the media; and *A_untreated_*—the absorbance of the solution containing untreated cells. The IC_50_ values were calculated using GraphPad Prism 10 software with the four-parameter dose–response.

## 4. Conclusions

In summary, our lab has successfully developed another class of potent flavone-based triplex-specific ligands. The amino-containing side chain at the 5-position in the flavone derivatives is important for binding affinity and selectivity. The length of the side chain can be used to fine-tune the thermal stabilization effect of 5-substituted flavones on triplex DNA. 5-Substituted flavones can inhibit enzymatic activities in vitro via ligand-mediated triplex formation, which affirms that triplex-binding ligands could be used as antigene enhancers in potential therapeutic applications. Several 5-substituted flavones with strong triplex binding affinities provide excellent cytotoxicity toward colorectal and leukemia cancer cells. Taking into account the previous report from our lab [28], we conclude that natural products, flavonoids, are promising scaffolds for the development of triplex DNA-binding ligands. Investigations of the potential of various flavonoid derivatives as triplex-specific ligands are currently being conducted in our lab, and the results will be reported in due course.

## Figures and Tables

**Table 1:** Thermodynamic parameters for ligands (**1**, **4a**, **4b**, **4c**, neomycin) binding to **DNA3** (triplex) and **DNA4** (duplex), obtained by ITC.

Ligand	n	K_d_ (×10^−5^ M)	ΔG (kJ/mol)	ΔH (kJ/mol)	−TΔS (kJ/mol)
**1**	N/A	N/A	N/A	N/A	N/A
**4a**	3.8 ± 0.5	2.10 ± 1.12	−25.8	−45.1 ± 14.0	19.3
**4b**	6.1 ± 0.2	1.01 ± 0.20	−27.6	−36.9 ± 1.6	9.4
**4c**	3.0 ± 0.7	3.08 ± 1.26	−24.9	−66.7 ± 27.4	41.8
**4b** ^a^	N/A	N/A	N/A	N/A	N/A

Note: N/A = not applicable. ^a^ This row is the titration of **4b** with **DNA4** (duplex). All other titrations are with **DNA3** (triplex).

**Table 2:** IC_50_ values obtained for various ligands against three cancer cell lines using the MTT assay.

Ligand	HL-60 (μM)	HT-29 (μM)	HCT116 (μM)
**1**	N/A	57.2	N/A
**4c**	19.0	29.0	19.2
**4b**	6.7	14.2	29.7
**4a**	8.8	18.9	39.0
**8c**	22.7	16.9	24.9
**8b**	4.6	12.5	7.9
**8a**	20.6	40.1	45.3

Note: The IC_50_ values were extrapolated using the model four-parameter dose–response curve from GraphPad Prism 10. N/A = no effect. For all data, confidence intervals > 95%.

## Data Availability

The original contributions presented in this study are included in the article/Supplementary Materials. Further inquiries can be directed to the corresponding author.

## Supplementary Materials

The following supporting information can be downloaded at https://www.mdpi.com/article/10.3390/molecules29245862/s1. Figure S1−S3, ITC profiles; Figure S4, the sequence of the constructed plasmid **DNA5**; Figure S5, densitometric analysis of the four *DraI* cleavage fragments from **DNA5**; Figure S6−S8, cell viability analysis; Figure S9−S60, ^1^H- and ^13^C-NMR spectra of all compounds; Figure S61−S84, HRMS spectra of representative compounds; Figure S85, comparison of ΔT_m3→2_ values between 5-substituted 3,3′,4′,7′-tetramethoxyflavonoids and 5-substituted flavones with the same linker and end amino groups.
