# Supplementary material for: 5-Substituted Flavones—Another Class of Potent Triplex DNA-Specific Ligands as Antigene Enhancers
Source: Molecules. 2024 Dec 12;29(24):5862. doi: 10.3390/molecules29245862 (PMC11678237; doi:10.3390/molecules29245862)
Supplement: Supplementary file 1 [file molecules-29-05862-s001.zip › molecules-3333454-supplementary.pdf]

Supplementary data: 5-Substituted flavones – Another class of potent DNA triplex-specific binding ligands as antigene enhancers

Landy Gu,[a] Nghia Tran,[a] Vanessa M. Rangel,[a] Mandeep Singh,[a] Krege M. Christison,[a] Geoff P. Lin-Cereghino,[b] and Liang Xue*[a]

[a]Department of Chemistry, University of the Pacific, 3601 Pacific Avenue, Stockton, California 95211 (USA)

[b]Department of Biological Sciences, University of the Pacific, 3601 Pacific Avenue, Stockton, California 95211 (USA)

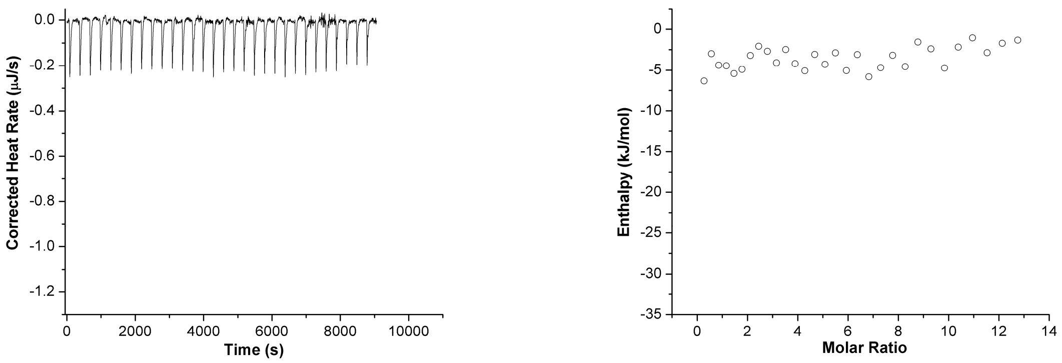

Supporting Information Figure S1. Left: ITC profile of **DNA3** (triplex, 10 µM) titrated with **1** (100 µM) in a 10 mM sodium cacodylate buffer with 100 mM NaCl, pH 7.0 at 15 °C. Right: The dotted lines represent the corrected injection heats plotted as a function of the [**1**]/[**DNA**] molar ratio. The smooth solid line reflects the calculated fit of the data using the independent model from the software NanoAnalyze.

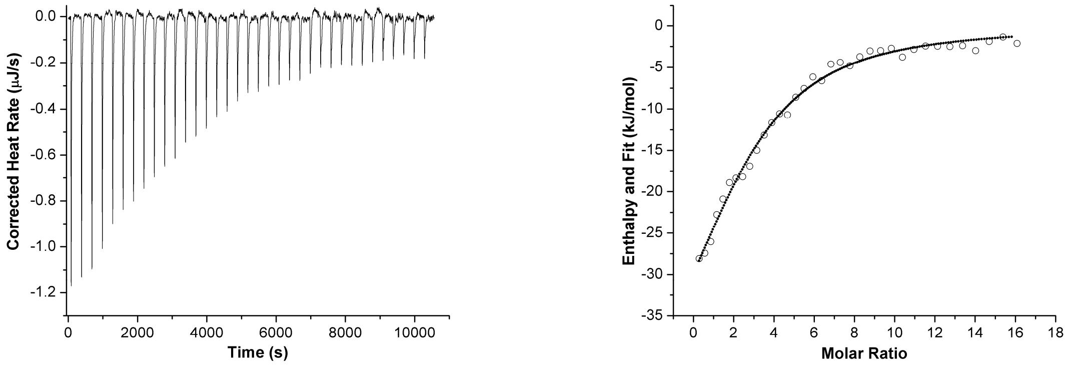

Supporting Information Figure S2. Left: ITC profile of **DNA3** (triplex, 10 μM) titrated with **4a** (100 μM) in a 10 mM sodium cacodylate buffer with 100 mM NaCl, pH 7.0 at 15 °C. Right: The dotted lines represent the corrected injection heats plotted as a function of the [**4a**]/[**DNA**] molar ratio. The smooth solid line reflects the calculated fit of the data using the independent model from the software NanoAnalyze.

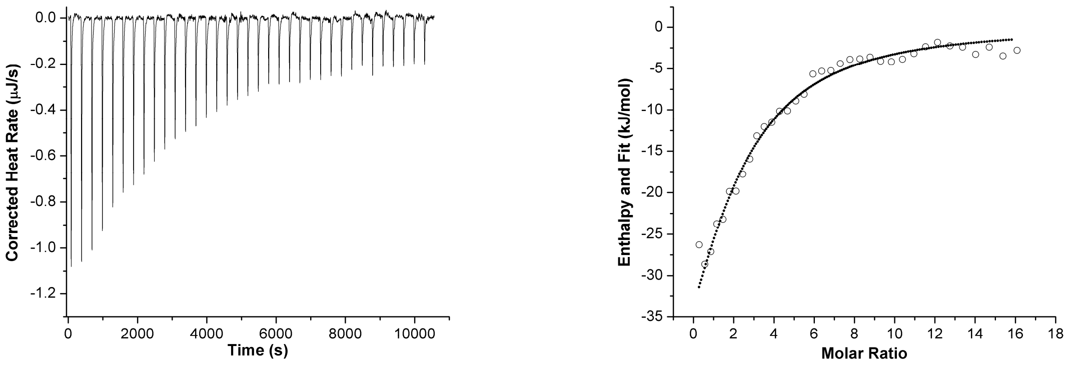

Supporting Information Figure S3. Left: ITC profile of **DNA3** (triplex, 10 μM) titrated with **4c** (100 μM) in a 10 mM sodium cacodylate buffer with 100 mM NaCl, pH 7.0 at 15 °C. Right: The dotted lines represent the corrected injection heats plotted as a function of the [**4c**]/[**DNA**] molar ratio. The smooth solid line reflects the calculated fit of the data using the independent model from the software NanoAnalyze.

CAGTCACGACGTTGTAAAACGACGGCCAGTGAGCGCGCGTAATACGACTCACTATAGGGCGAATTGGGTACCTTT
AAAAAAAAAAAAAAAATGGTATCAAAAGGAGAGGAAGATAATATGGCGATTATTAAAGAATTCATGCGTTTTAAG
GTGCATATGGAAGGTAGTGTTAATGGTCACGAATTCGAGATCGAGGGTGAGGGCGAGGGTCGTCCGTATGAAGG
CACTCAAACCGCAAAATTGAAGGTAACGAAAGGCGGACCGCTCCCTTTCGCTTGGGATATCCTGTCCCCGCAGTT
TATGTACGGCAGCAAAGCGTATGTCAAACACCCGGCTGATATCCCGGATTACCTGAAGTTGTCGTTCCCGGAAGGT
TTTAAGTGGGAGCGCGTGATGAACTTTGAGGACGGTGGCGTCGTGACGGTGACCCAAGACAGCTCCCTGCAGG
ACGGCGAGTTCATCTACAAGGTGAAACTGCGTGGTACGAACTTCCCAAGCGATGGCCCGGTTATGCAGAAAAAG
ACCATGGGTTGGGAGGCCAGCTCTGAACGCATGTACCCGGAGGACGGCGCGCTGAAAGGTGAAATCAAGCAGC
GTTTGAAGTTAAAGGACGGCGGCCACTACGACGCGGAAGTTAAAACCACCTATAAGGCCAAAAAGCCGGTTCAG
CTGCCGGGTGCGTACAACGTGAACATTAAGCTGGATATTACCAGCCATAATGAAGACTACACCATTGTTGAACAAT
ATGAGCGCGCAGAGGGTCGTCATAGCACCGGTGGTATGGATGAACTGTATAAAGGTACCGGGCCCCCCCTCGAG
GTCGACGGTATCGATAAGCTTGATATCGAATTCCTGCAGCCCGGGGGATCCACTAGTTCTAGAGCGGCCGCCACCG
CGGTGGAGCTCCAGCTTTTGTTCCCTTTAGTGAGGGTTAATTGCGCGCTTGGCGTAATCATGGTCATAGCTGTTTC
CTGTGTGAAATTGTTATCCGCTCACAATTCCACACAACATACGAGCCGGAAGCATAAAGTGTAAAGCCTGGGGTGC
CTAATGAGTGAGCTAACTCACATTAATTGCGTTGCGCTCACTGCCCGCTTTCCAGTCGGGAAACCTGTCGTGCCAG
CTGCATTAATGAATCGGCCAACGCGCGGGGAGAGGCGGTTTGCGTATTGGGCGCTCTTCCGCTTCCTCGCTCACT
GACTCGCTGCGCTCGGTCGTTCGGCTGCGGCGAGCGGTATCAGCTCACTCAAAGGCGGTAATACGGTTATCCACA
GAATCAGGGGATAACGCAGGAAAGAACATGTGAGCAAAAGGCCAGCAAAAGGCCAGGAACCGTAAAAAGGCC
GCGTTGCTGGCGTTTTTCCATAGGCTCCGCCCCCCTGACGAGCATCACAAAAATCGACGCTCAAGTCAGAGGTGG
CGAAACCCGACAGGACTATAAAGATACCAGGCGTTTCCCCCTGGAAGCTCCCTCGTGCGCTCTCCTGTTCCGACCC
TGCCGCTTACCGGATACCTGTCCGCCTTTCTCCCTTCGGGAAGCGTGGCGCTTTCTCATAGCTCACGCTGTAGGTAT
CTCAGTTCGGTGTAGGTCGTTCGCTCCAAGCTGGGCTGTGTGCACGAACCCCCCGTTCAGCCCGACCGCTGCGCC
TTATCCGGTAACTATCGTCTTGAGTCCAACCCGGTAAGACACGACTTATCGCCACTGGCAGCAGCCACTGGTAACA
GGATTAGCAGAGCGAGGTATGTAGGCGGTGCTACAGAGTTCTTGAAGTGGTGGCCTAACTACGGCTACACTAGAA
GGACAGTATTTGGTATCTGCGCTCTGCTGAAGCCAGTTACCTTCGGAAAAAGAGTTGGTAGCTCTTGATCCGGCA
AACAAACCACCGCTGGTAGCGGTGGTTTTTTTGTTTGCAAGCAGCAGATTACGCGCAGAAAAAAAGGATCTCAA
GAAGATCCTTTGATCTTTTCTACGGGGTCTGACGCTCAGTGGAACGAAAACTCACGTTAAGGGATTTTGGTCATG
AGATTATCAAAAAGGATCTTCACCTAGATCCTTTTAAATTAAAAATGAAGTTTTAAATCAATCTAAAGTATATATGAG
TAAACTTGGTCTGACAGTTACCAATGCTTAATCAGTGAGGCACCTATCTCAGCGATCTGTCTATTTCGTTCATCCATA
GTTGCCTGACTCCCCGTCGTGTAGATAACTACGATACGGGAGGGCTTACCATCTGGCCCCAGTGCTGCAATGATAC
CGCGAGACCCACGCTCACCGGCTCCAGATTTATCAGCAATAAACCAGCCAGCCGGAAGGGCCGAGCGCAGAAGT
GGTCCTGCAACTTTATCCGCCTCCATCCAGTCTATTAATTGTTGCCGGGAAGCTAGAGTAAGTAGTTCGCCAGTTAA
TAGTTTGCGCAACGTTGTTGCCATTGCTACAGGCATCGTGGTGTCACGCTCGTCGTTTGGTATGGCTTCATTCAGCT
CCGGTTCCCAACGATCAAGGCGAGTTACATGATCCCCCATGTTGTGCAAAAAAGCGGTTAGCTCCTTCGGTCCTCC
GATCGTTGTCAGAAGTAAGTTGGCCGCAGTGTTATCACTCATGGTTATGGCAGCACTGCATAATTCTCTTACTGTCA
TGCCATCCGTAAGATGCTTTTCTGTGACTGGTGAGTACTCAACCAAGTCATTCTGAGAATAGTGTATGCGGCGACC
GAGTTGCTCTTGCCCGGCGTCAATACGGGATAATACCGCGCCACATAGCAGAACTTTAAAAGTGCTCATCATTGGA
AAACGTTCTTCGGGGCGAAAACTCTCAAGGATCTTACCGCTGTTGAGATCCAGTTCGATGTAACCCACTCGTGCAC
CCAACTGATCTTCAGCATCTTTTACTTTCACCAGCGTTTCTGGGTGAGCAAAAACAGGAAGGCAAAATGCCGCAA
AAAAGGGAATAAGGGCGACACGGAAATGTTGAATACTCATACTCTTCCTTTTTCAATATTATTGAAGCATTTATCAG
GGTTATTGTCTCATGAGCGGATACATATTTGAATGTATTTAGAAAAATAAACAAATAGGGGTTCCGCGCACATTTCC
CCGAAAAGTGCCAC-3’

Supporting Information Figure S4. The entire sequence of the constructed plasmid (**DNA5**).

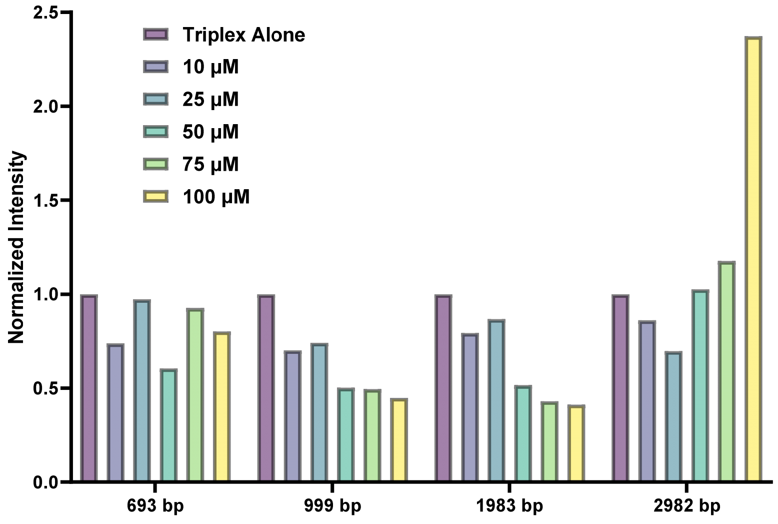

Supporting Information Figure S5. Densitometric analysis of the four *DraI* cleavage fragments from **DNA5** under various conditions. The values are normalized to the triplex band alone.

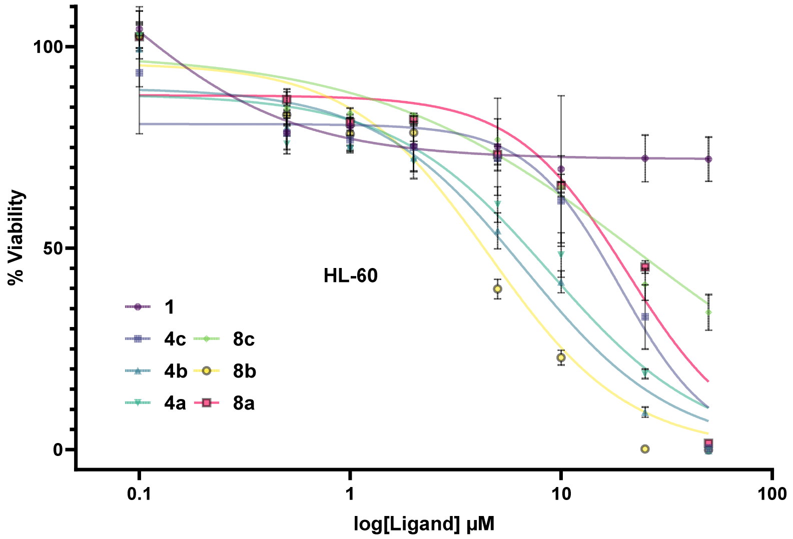

Supporting Information Figure S6. Viability (%) of HL-60 cancer cells as a function of increasing concentrations of ligands (**1**, **4a**, **4b**, **4c**, **8a**, **8b**, and **8c**)

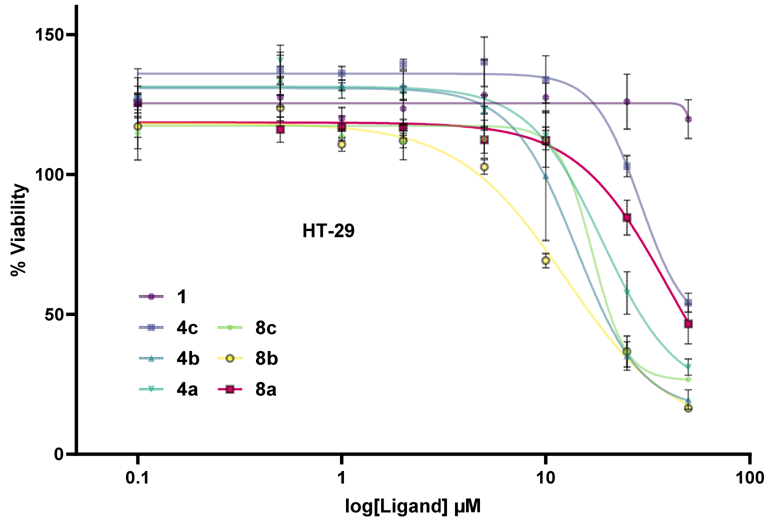

Supporting Information Figure S7. Viability (%) of HT-29 cancer cells as a function of increasing concentrations of ligands (**1**, **4a**, **4b**, **4c**, **8a**, **8b**, and **8c**)

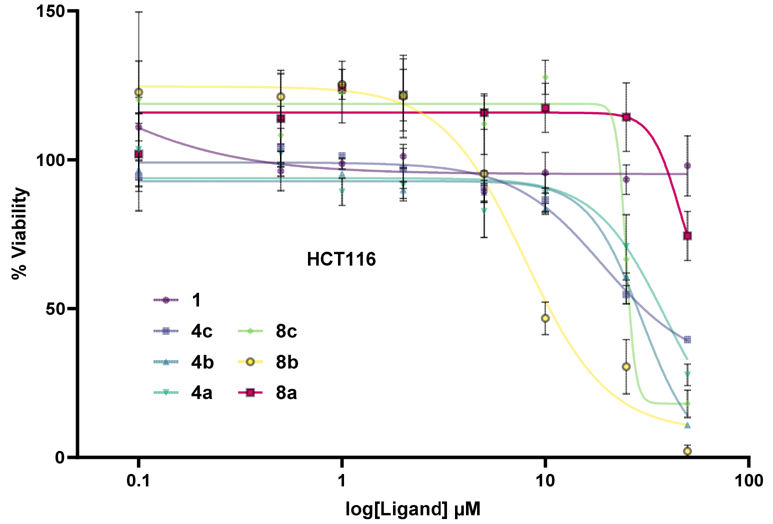

Supporting Information Figure S8. Viability (%) of HCT116 cancer cells as a function of increasing concentrations of ligands (**1**, **4a**, **4b**, **4c**, **8a**, **8b**, and **8c**)

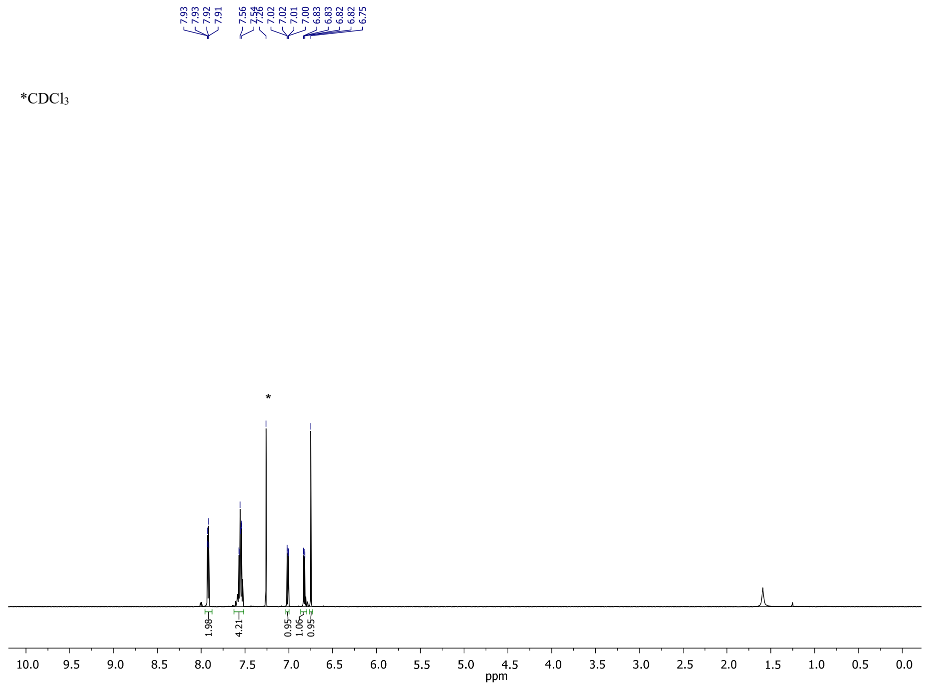

Supporting Information Figure S9. ^{1}H NMR of **1**.

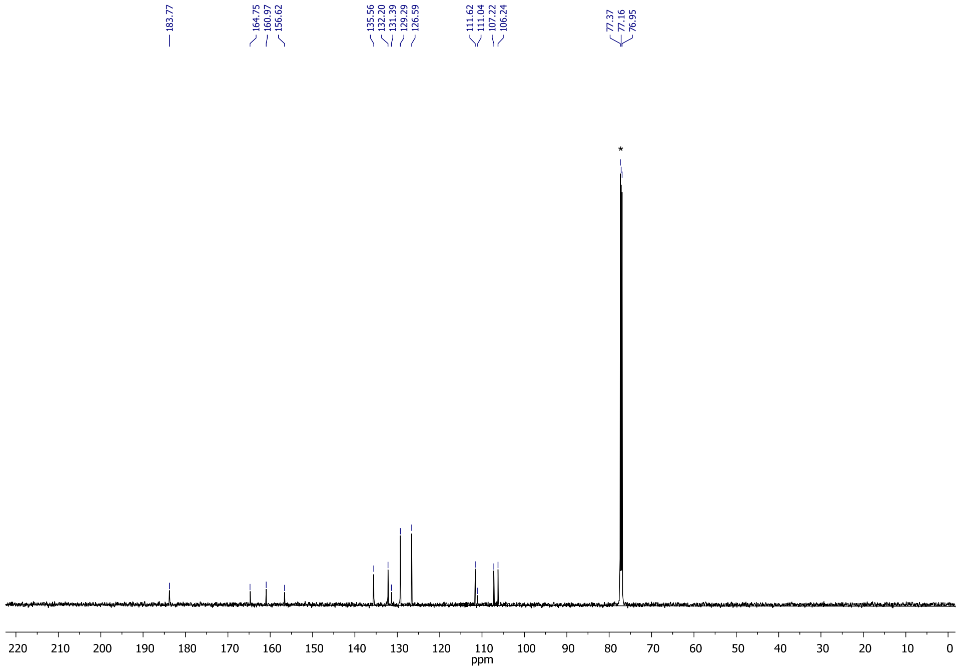

Supporting Information Figure S10. ^{13}C NMR of **1**.

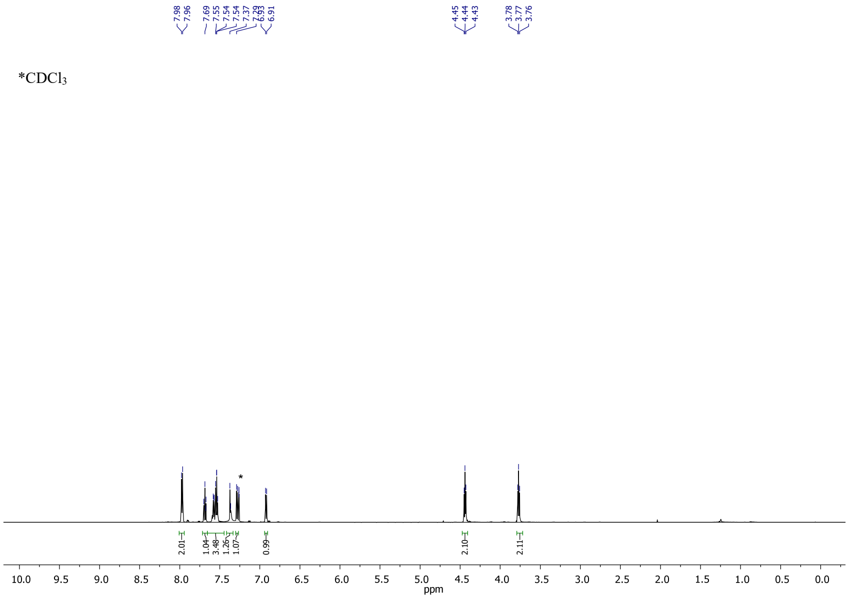

Supporting Information Figure S11. ^{1}H NMR of **2a**.

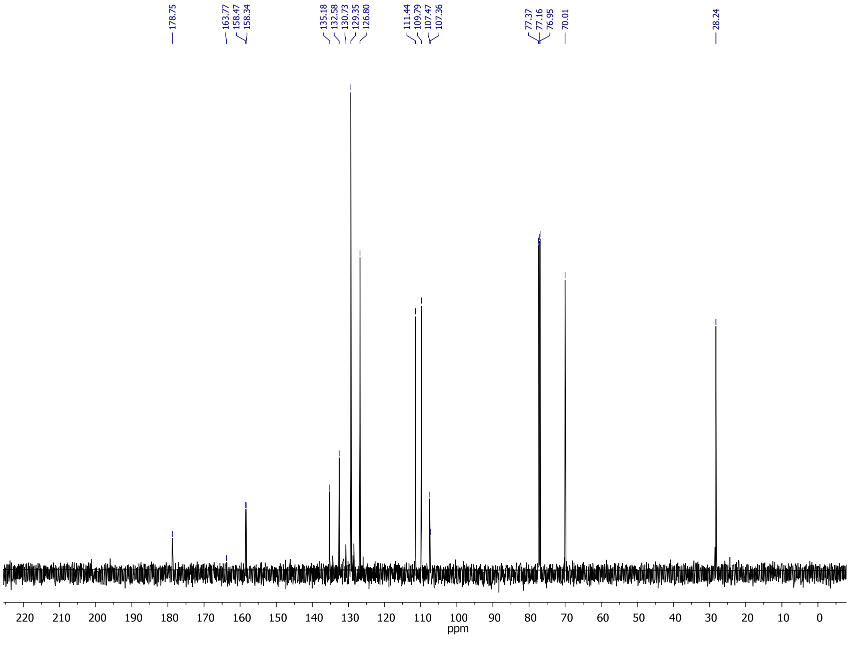

Supporting Information Figure S12. ^{13}C NMR of **2a**.

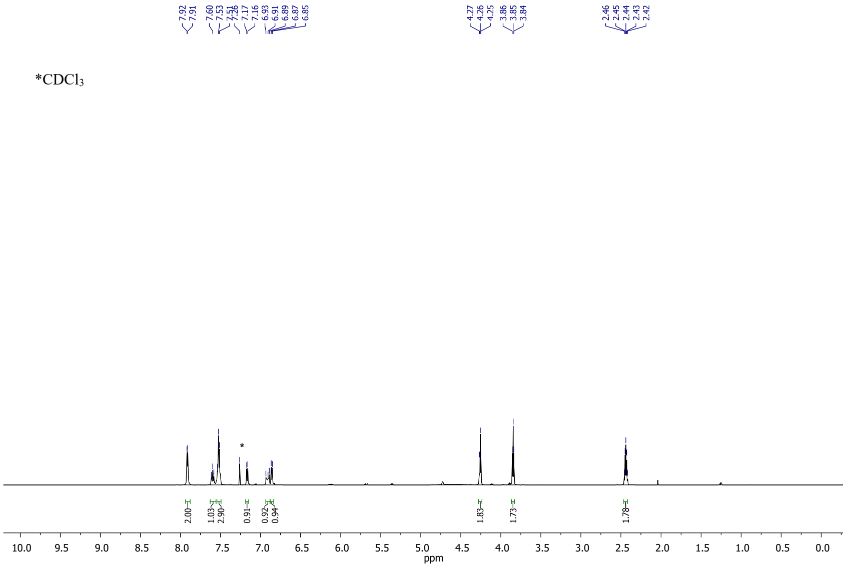

Supporting Information Figure S13. ^{1}H NMR of **2b**.

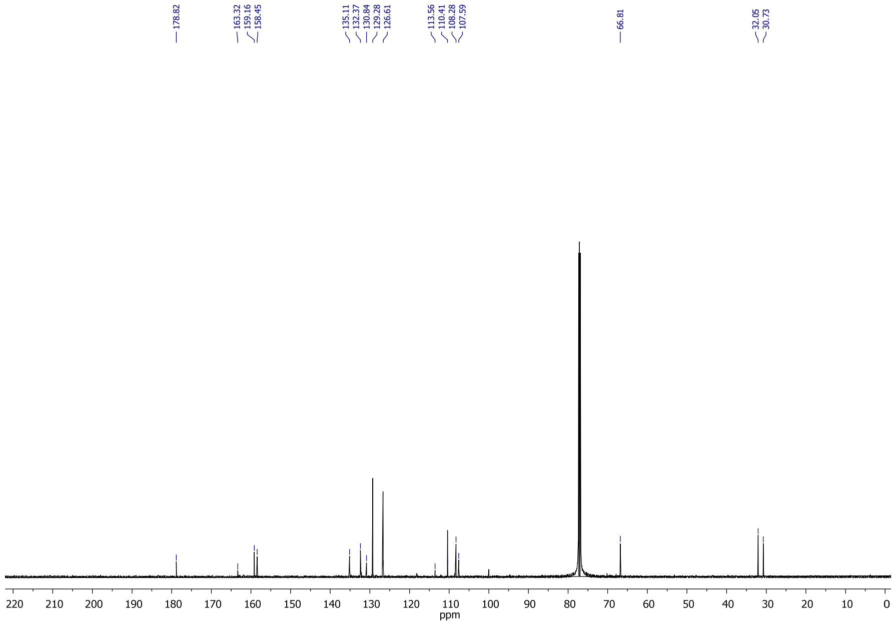

Supporting Information Figure S14. ^{13}C NMR of **2b**.

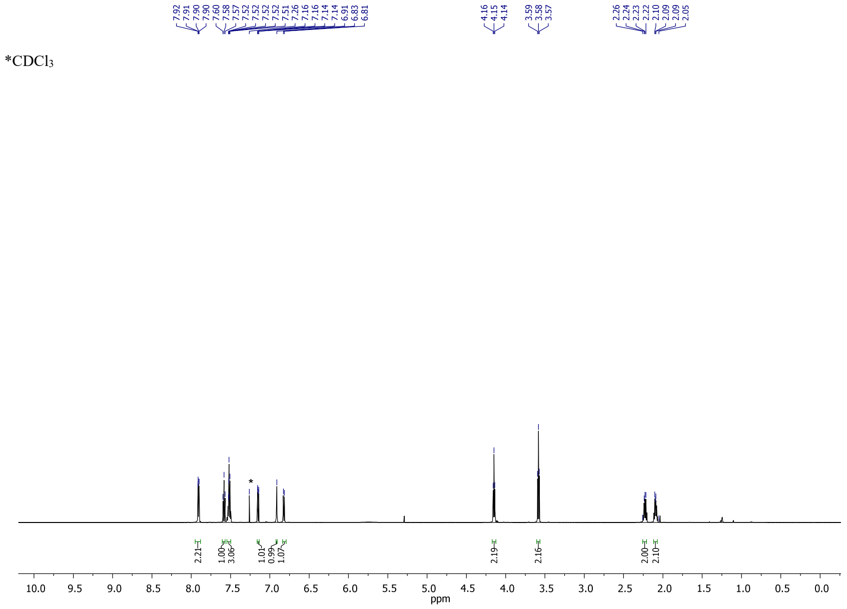

Supporting Information Figure S15. ^{1}H NMR of **2c**.

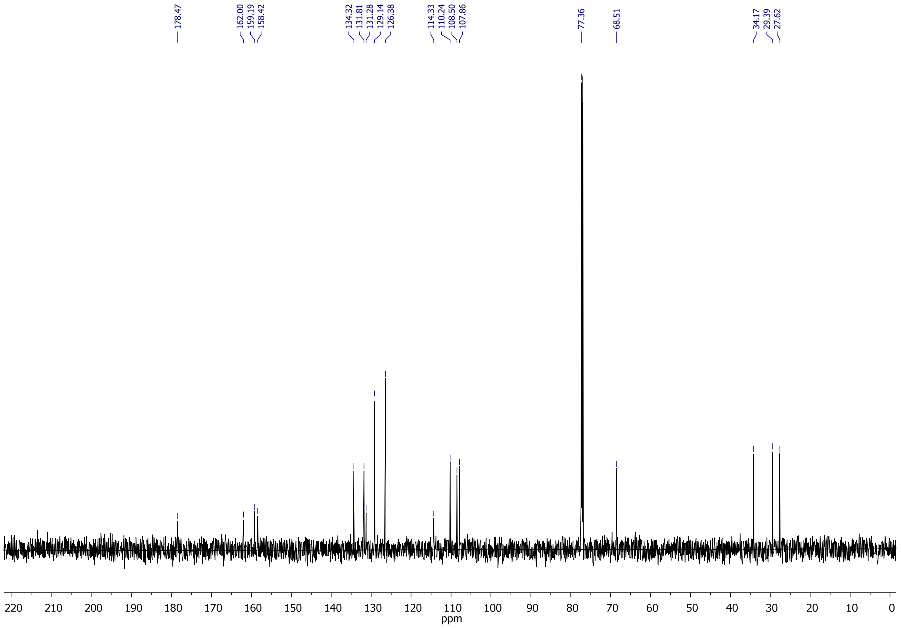

Supporting Information Figure S16. ^{13}C NMR of **2c**.

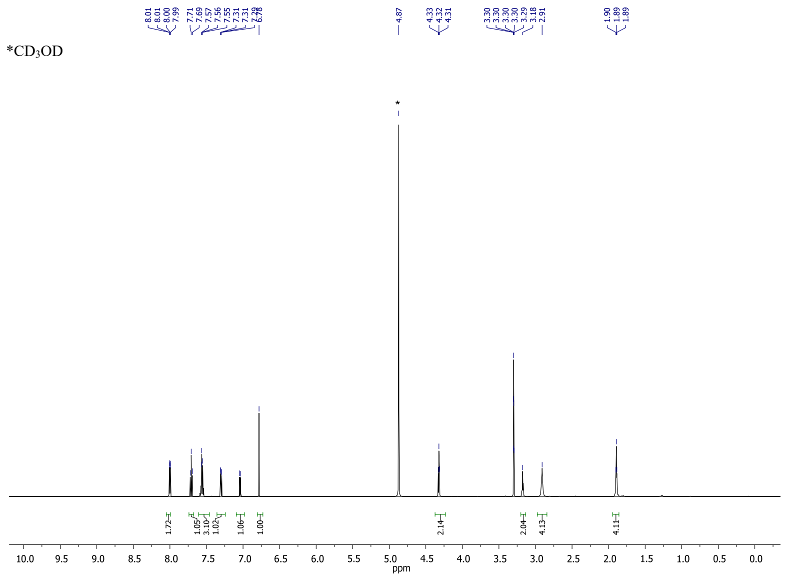

Supporting Information Figure S17. ^{1}H NMR of **3a**.

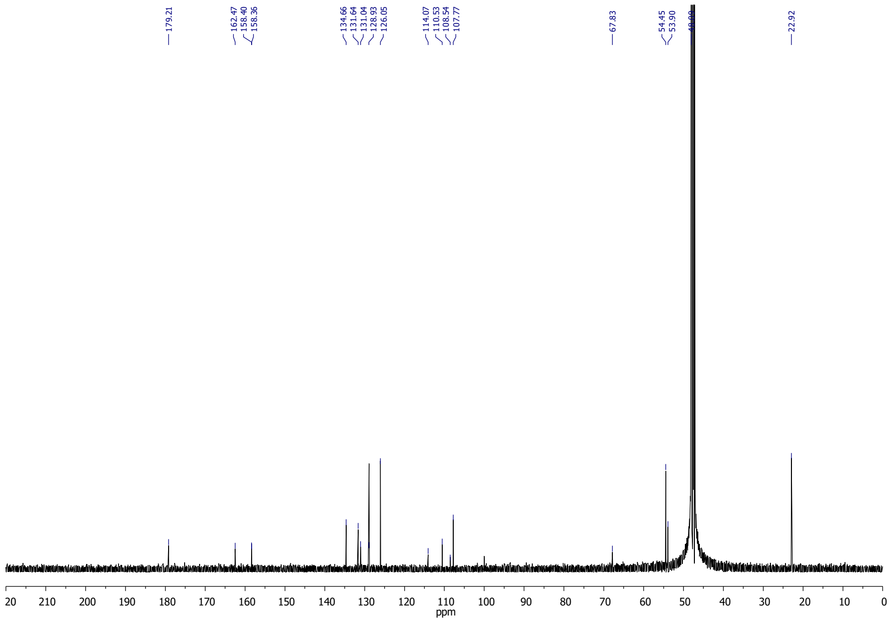

Supporting Information Figure S18. ^{13}C NMR of **3a**.

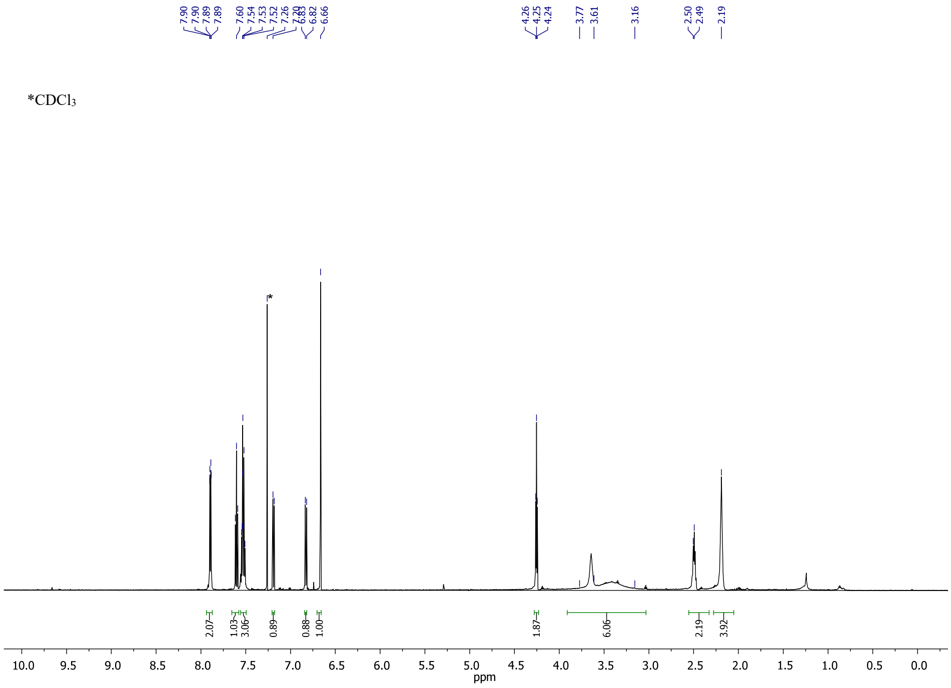

Supporting Information Figure S19. ^{1}H NMR of **3b**.

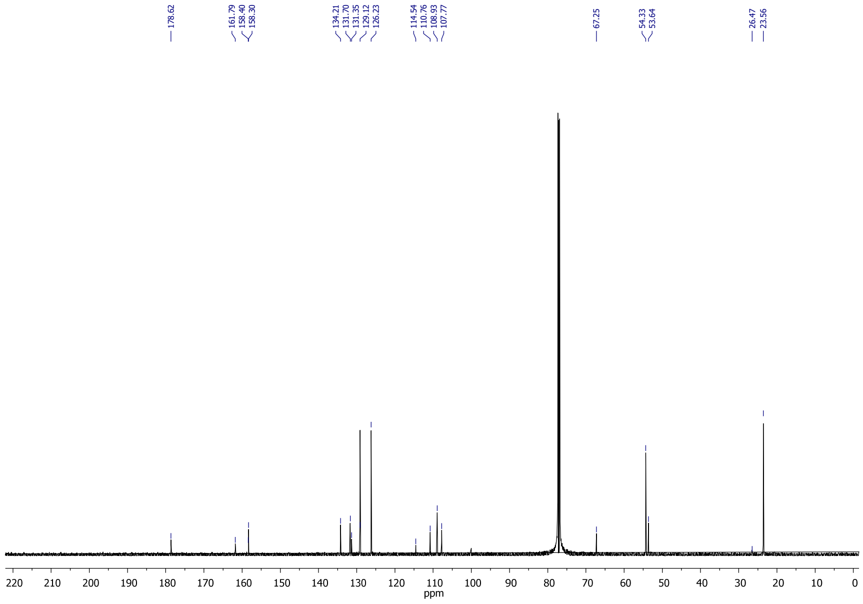

Supporting Information Figure S20. ^{13}C NMR of **3b**.

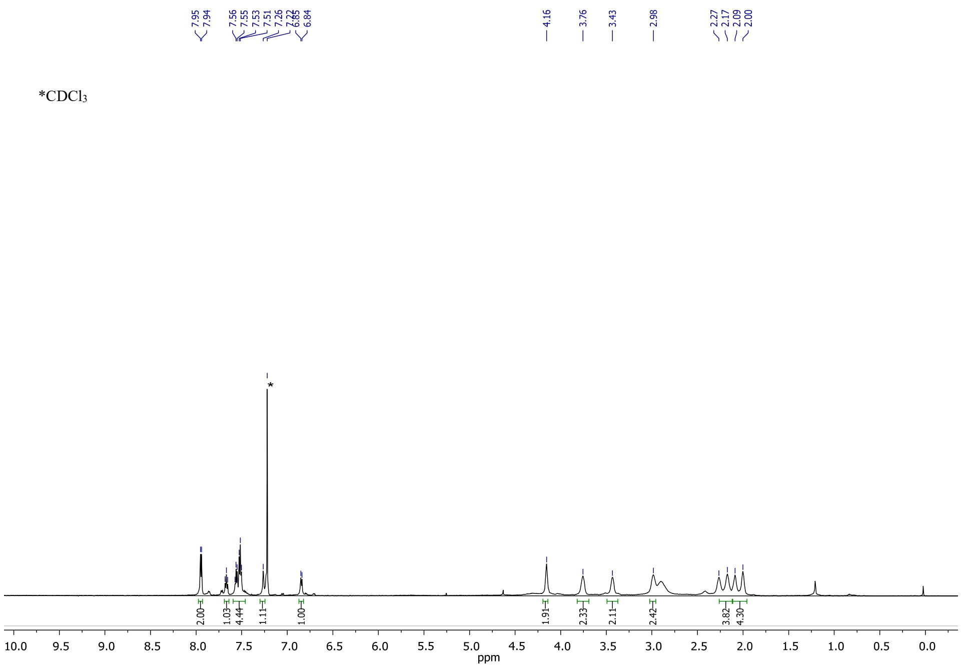

Supporting Information Figure S21. ^{1}H NMR of **3c**.

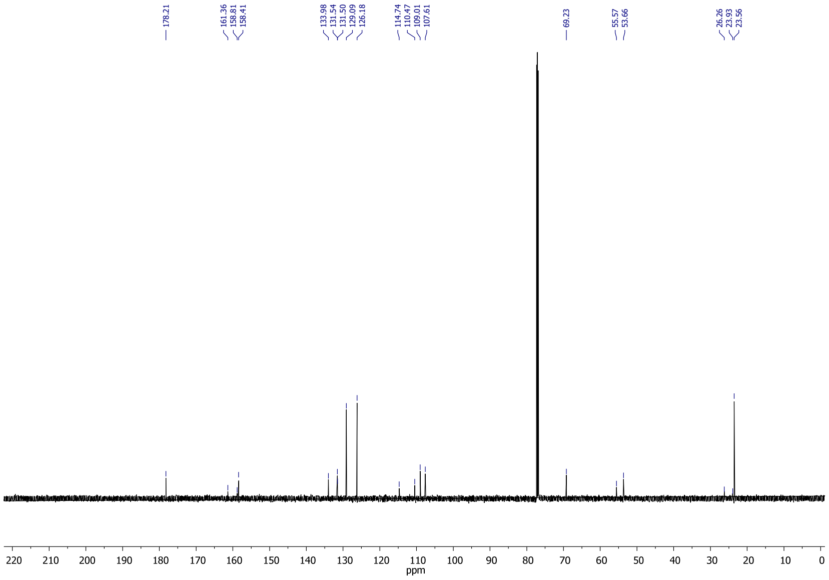

Supporting Information Figure S22. ^{13}C NMR of **3c**.

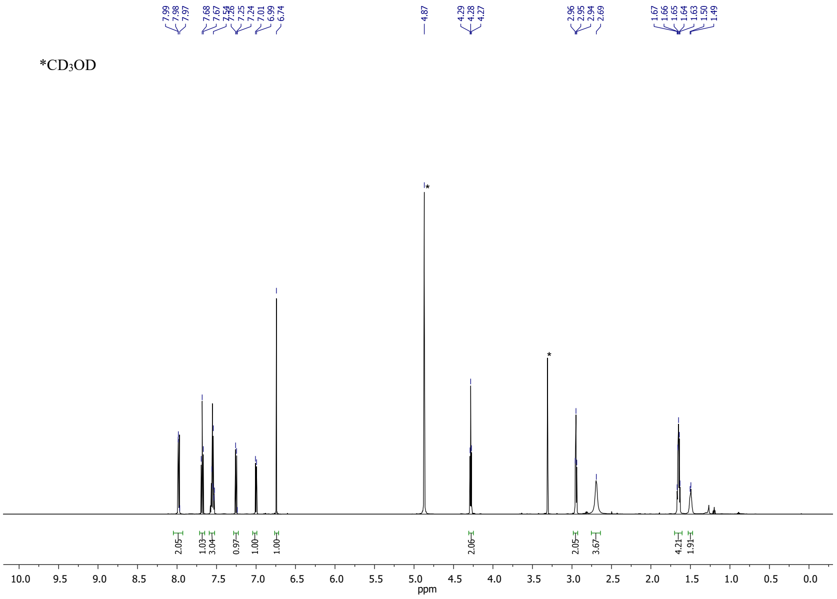

Supporting Information Figure S23. ^{1}H NMR of **4a**.

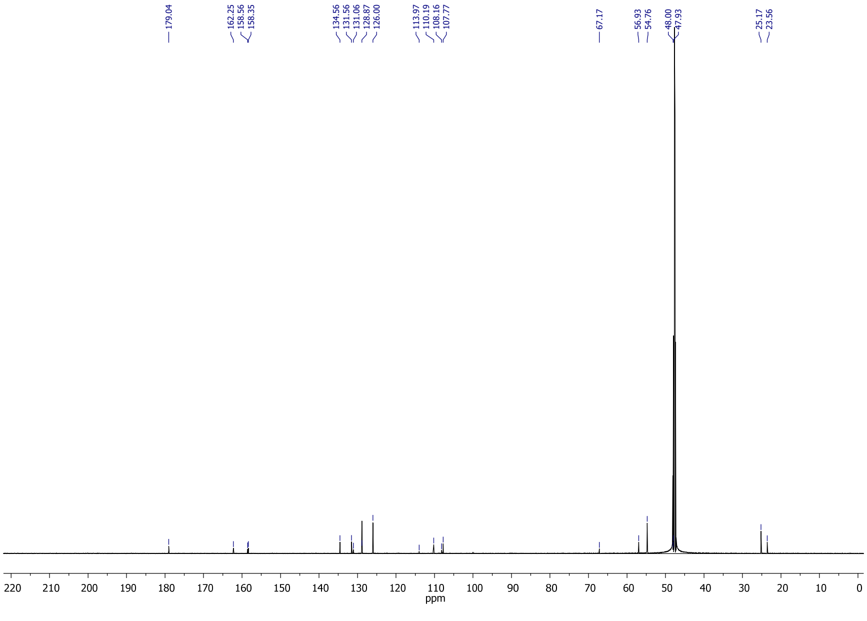

Supporting Information Figure S24. ^{13}C NMR of **4a**.

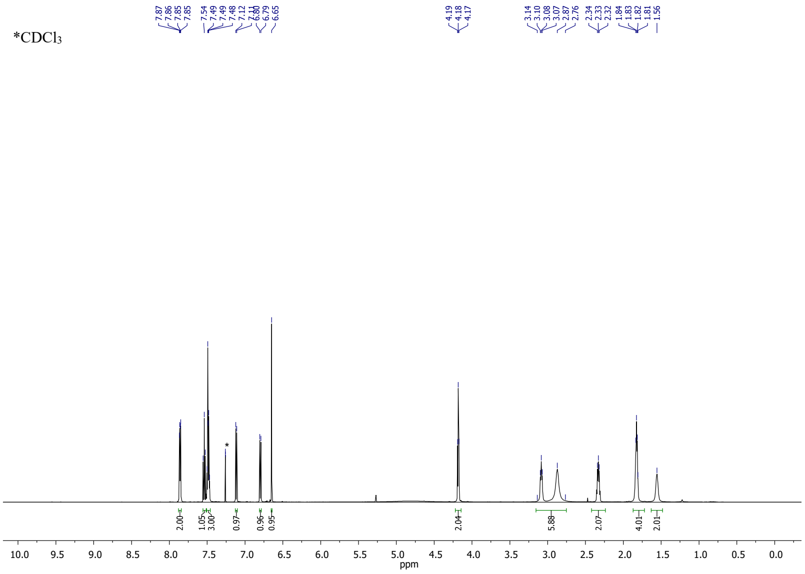

Supporting Information Figure S25. ^{1}H NMR of **4b**.

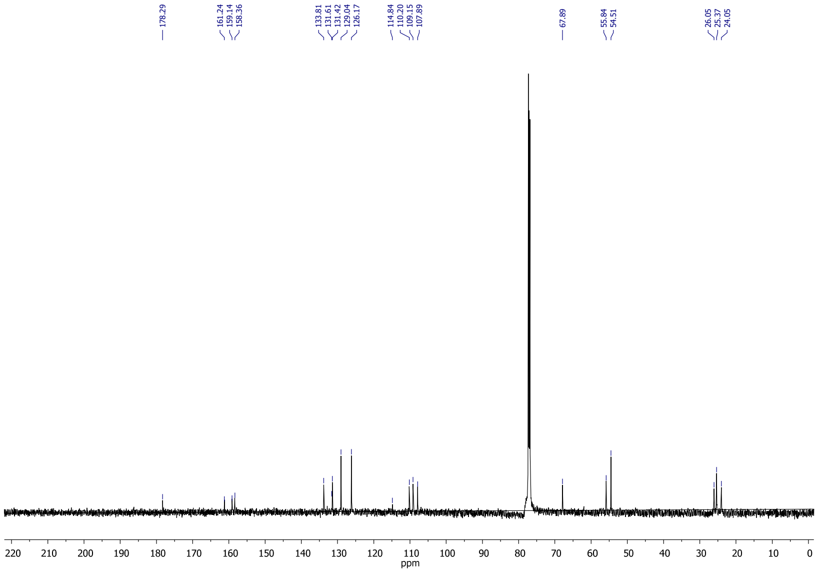

Supporting Information Figure S26. ^{13}C NMR of **4b**.

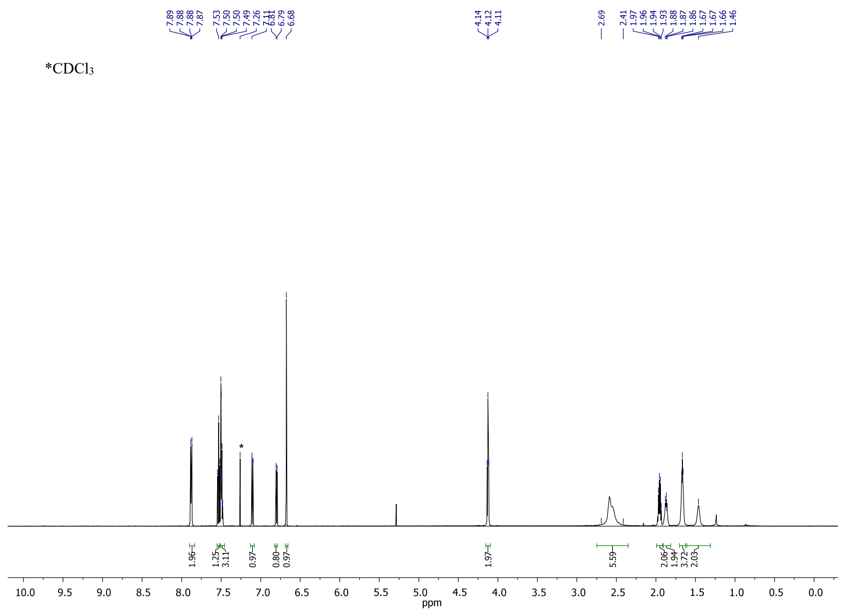

Supporting Information Figure S27. ^{1}H NMR of **4c**.

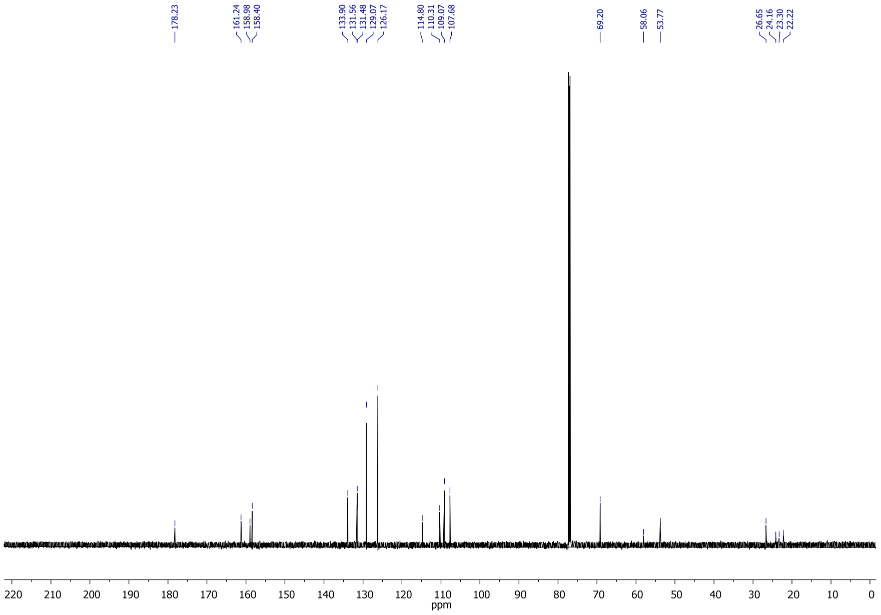

Supporting Information Figure S28. ^{13}C NMR of **4c**.

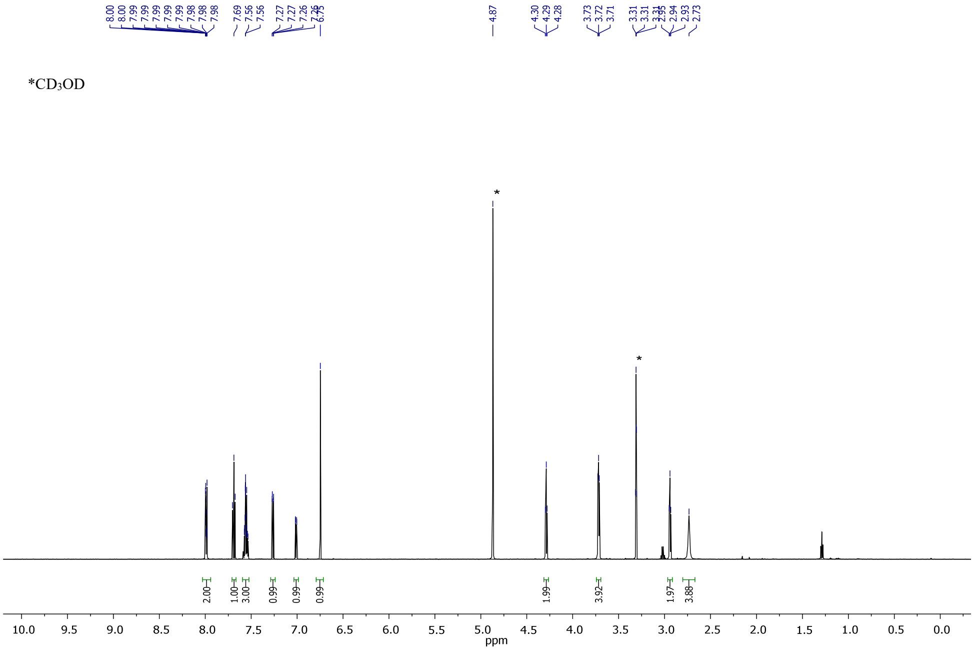

Supporting Information Figure S29. ^{1}H NMR of **5a**.

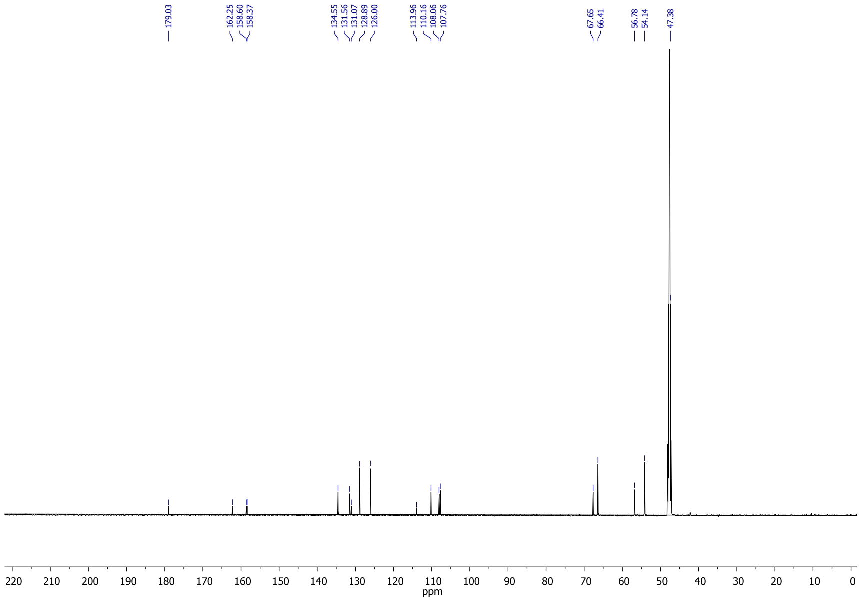

Supporting Information Figure S30. ^{13}C NMR of **5a**

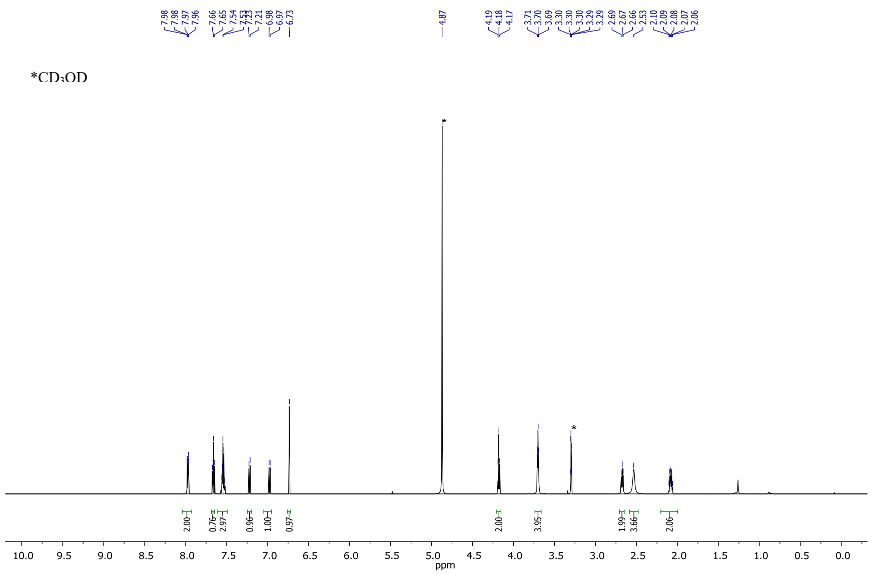

Supporting Information Figure S31. ^{1}H NMR of **5b**.

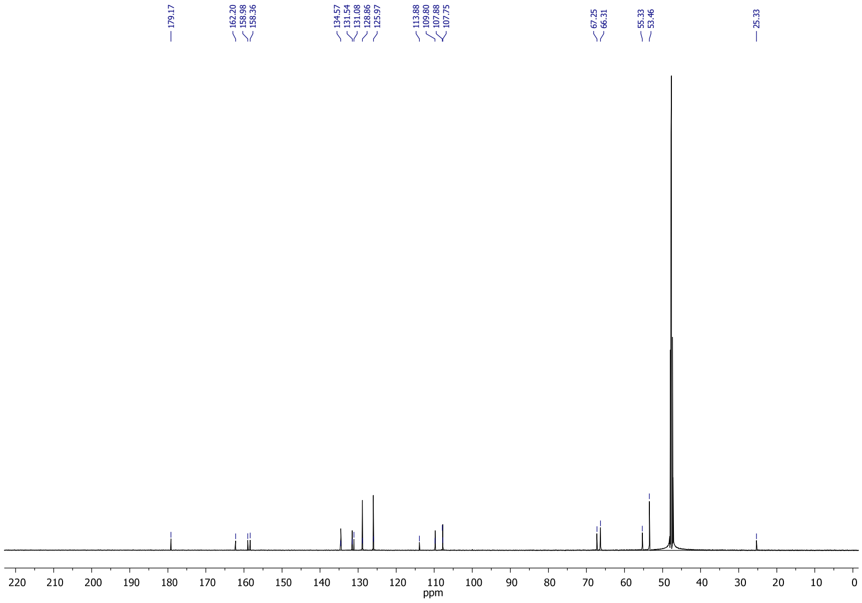

Supporting Information Figure S32. ^{13}C NMR of **5b**.

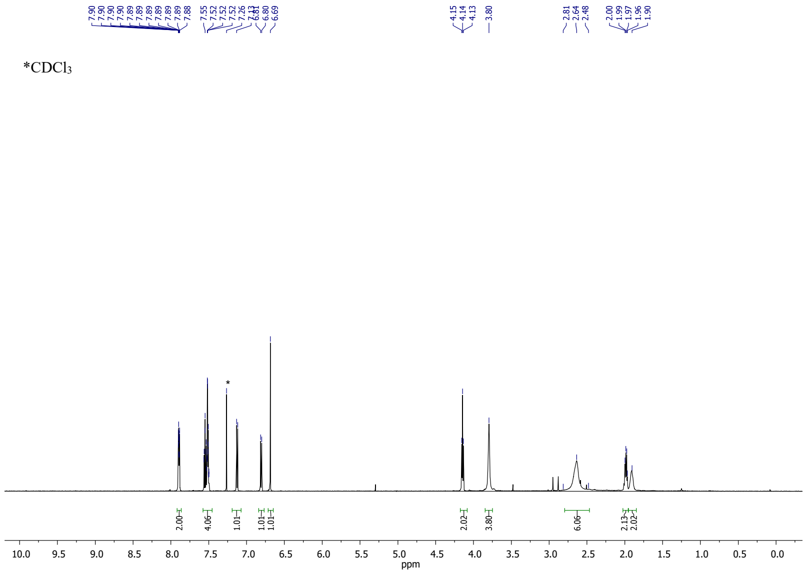

Supporting Information Figure S33. ^{1}H NMR of **5c**.

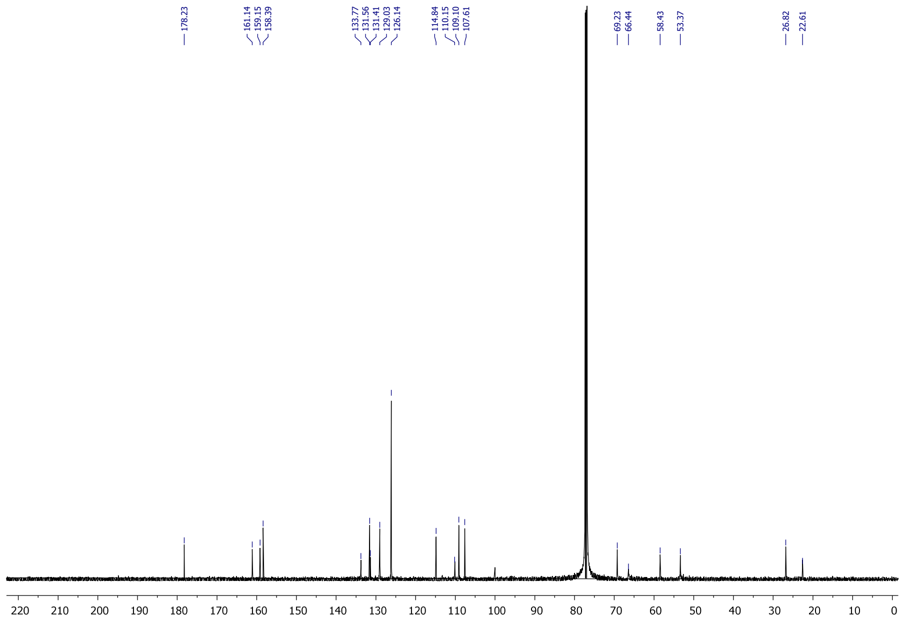

Supporting Information Figure S34. ^{13}C NMR of **5c**.

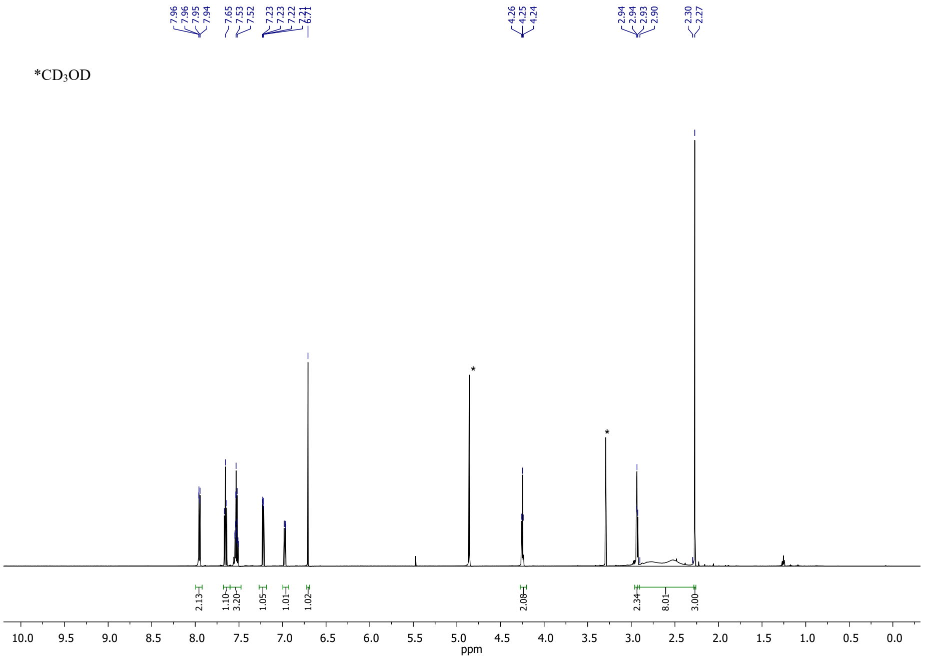

Supporting Information Figure S35. ^{1}H NMR of **6a**.

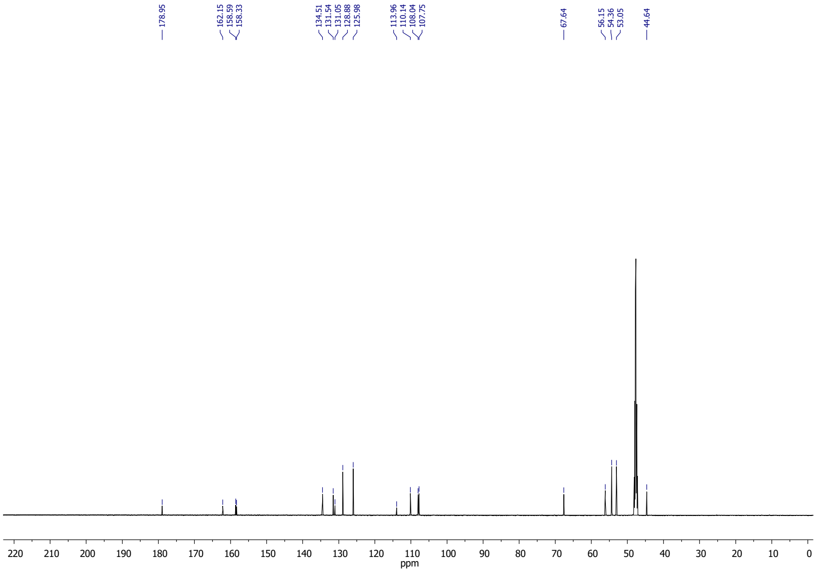

Supporting Information Figure S36. ^{13}C NMR of **6a**.

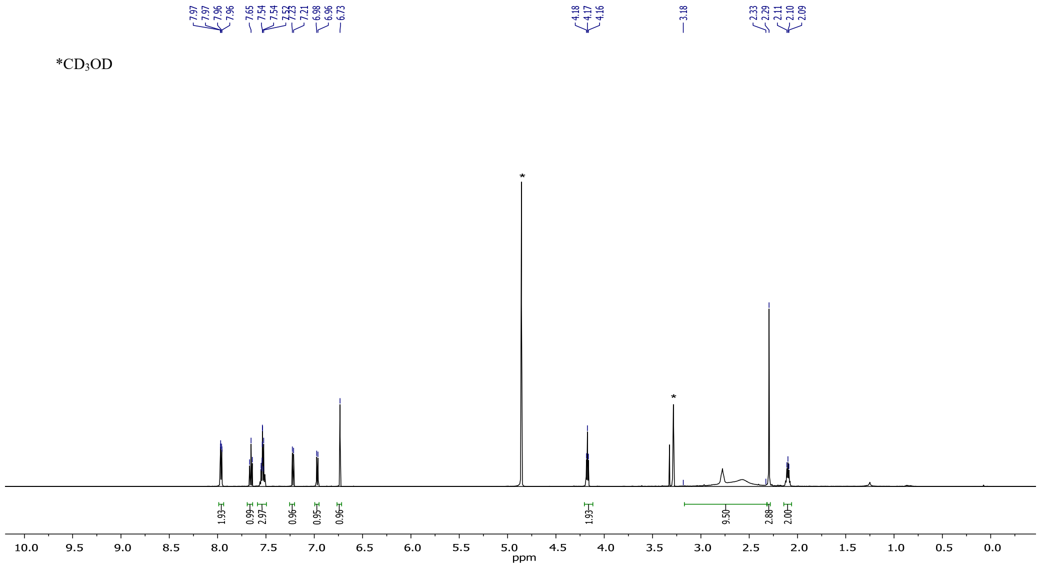

Supporting Information Figure S37. ^{1}H NMR of **6b**.

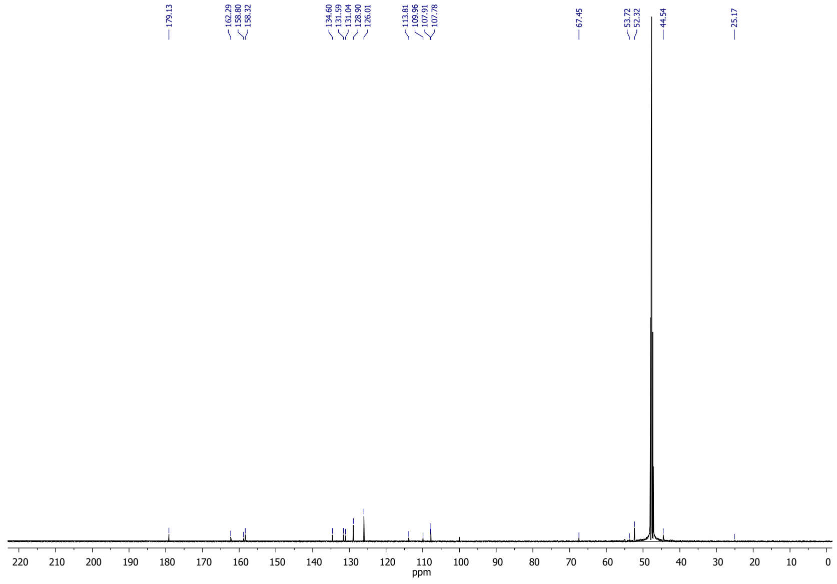

Supporting Information Figure S38. ^{13}C NMR of **6b**.

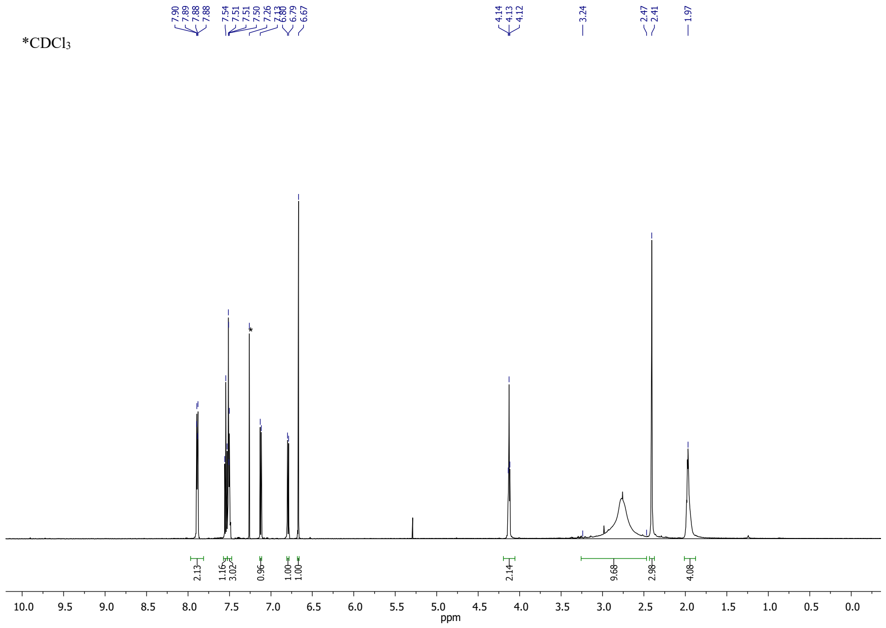

Supporting Information Figure S39. ^{1}H NMR of **6c**.

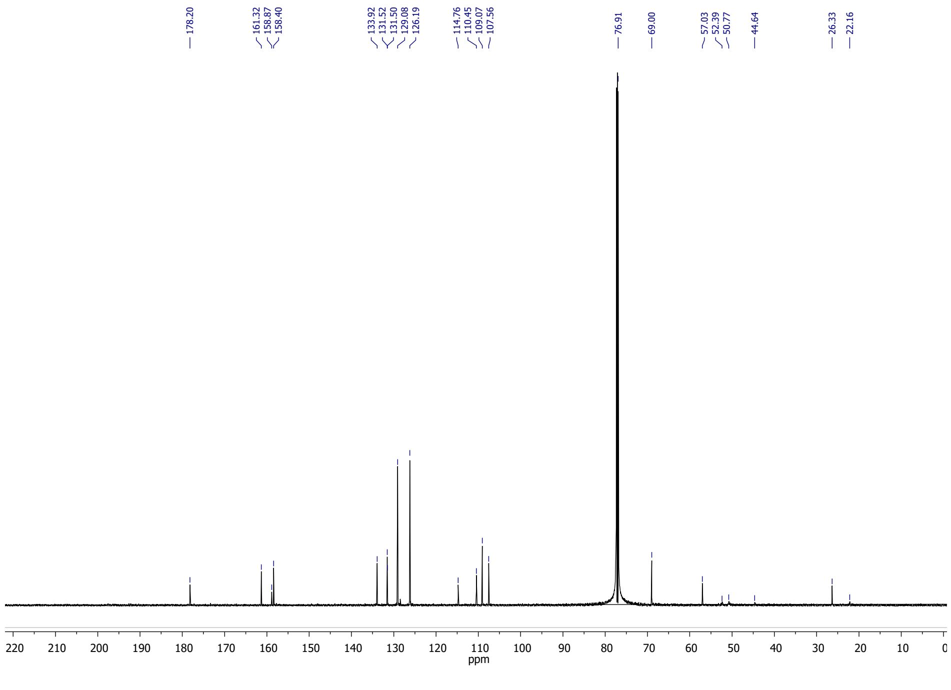

Supporting Information Figure S40. ^{13}C NMR of **6c**.

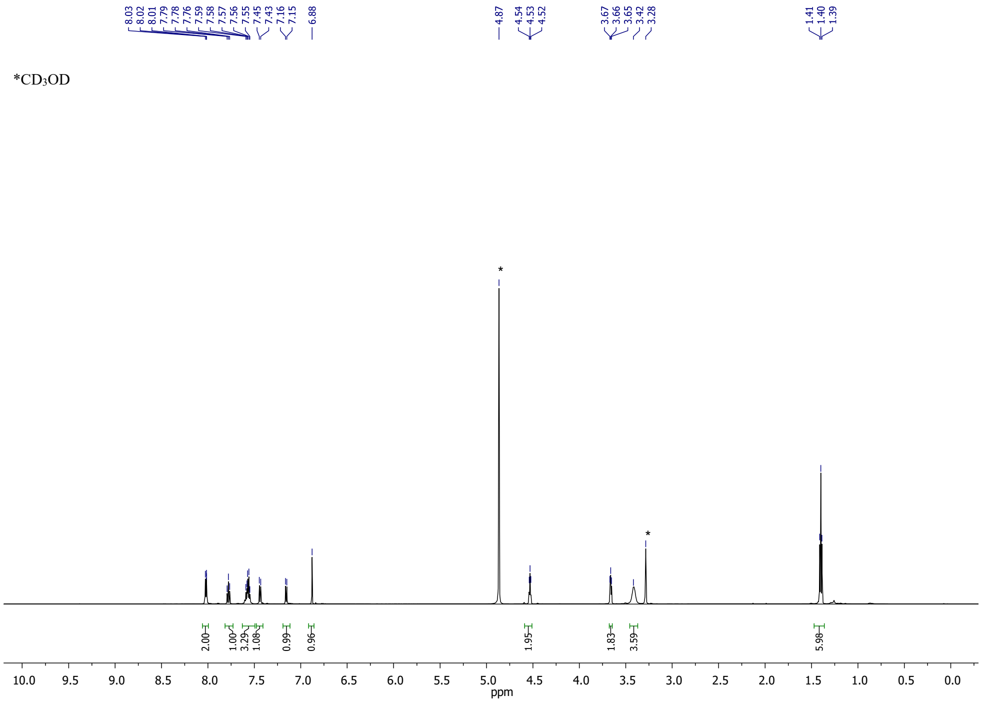

Supporting Information Figure S41. ^{1}H NMR of **7a**.

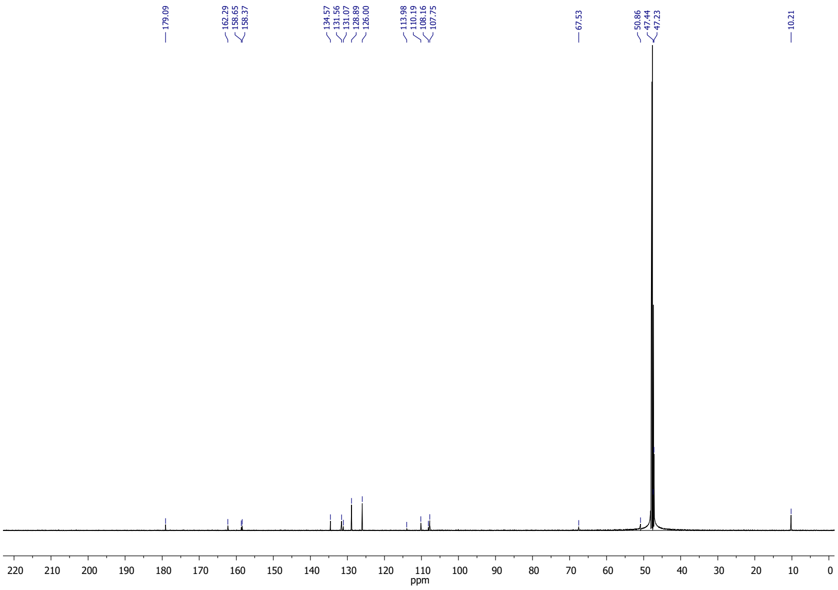

Supporting Information Figure S42. ^{13}C NMR of **7a**.

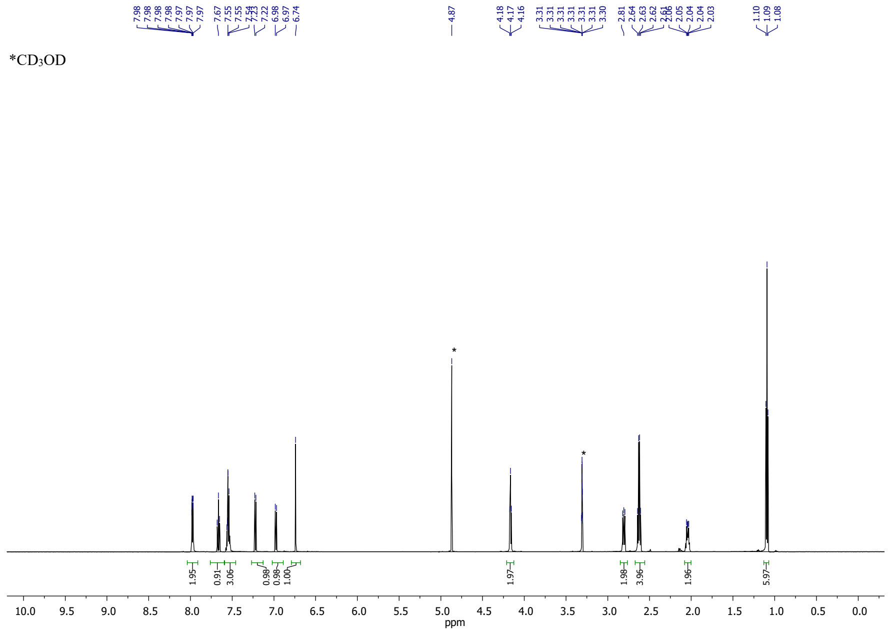

Supporting Information Figure S43. ^{1}H NMR of **7b**.

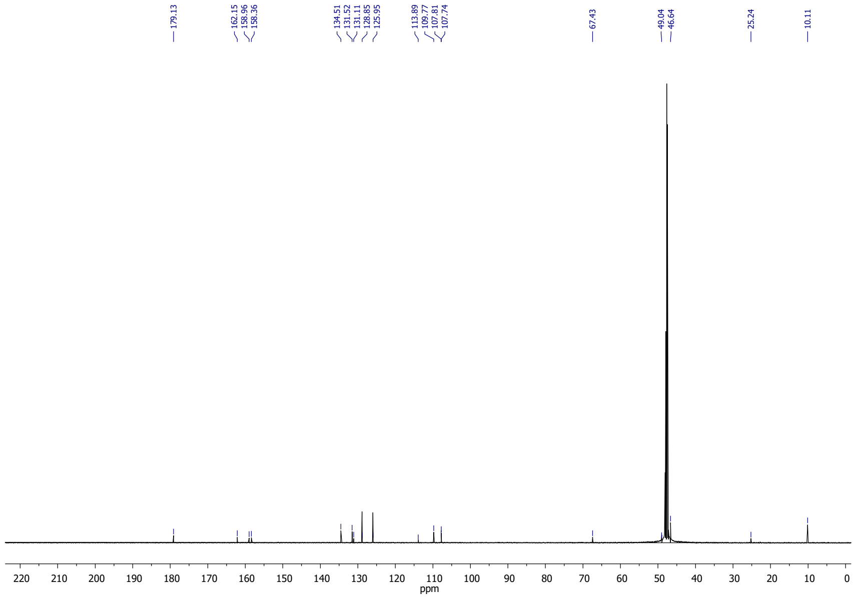

Supporting Information Figure S44. ^{13}C NMR of **7b**.

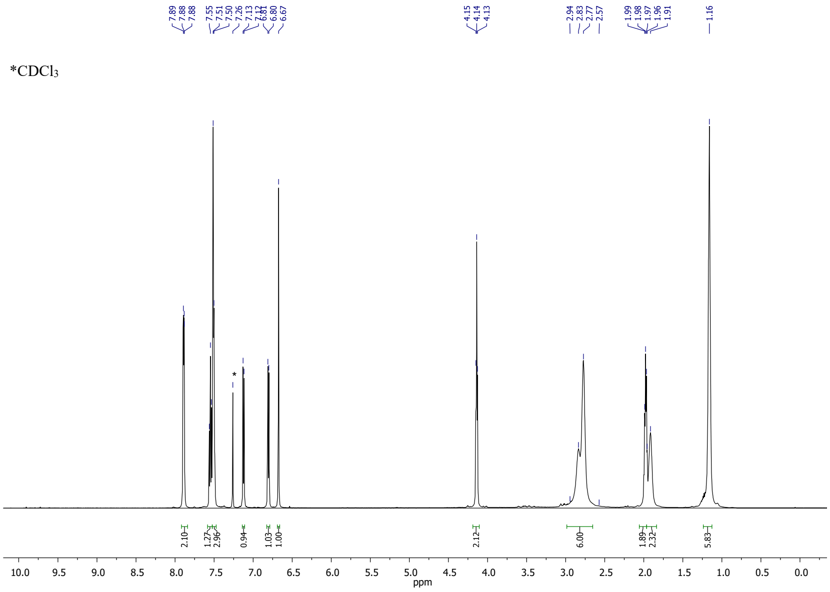

Supporting Information Figure S45. ^{1}H NMR of **7c**.

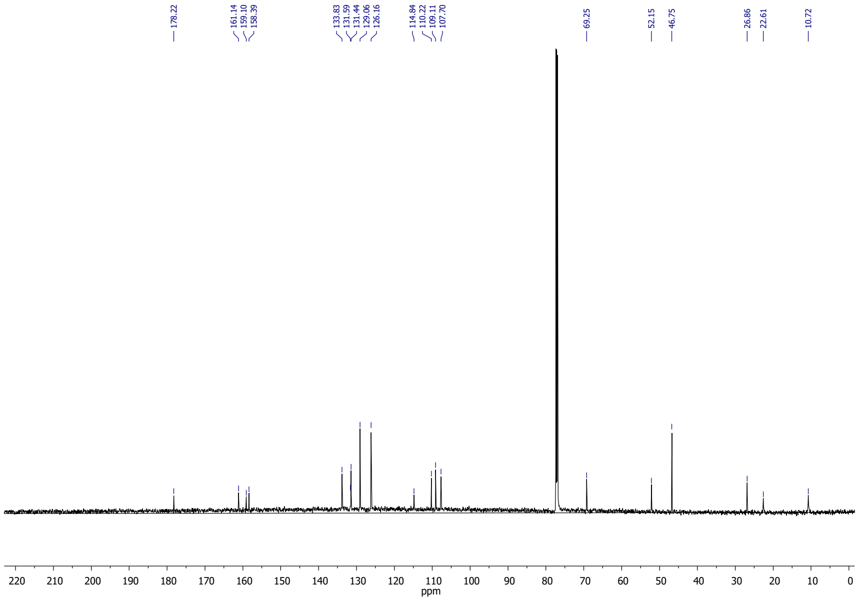

Supporting Information Figure S46. ^{13}C NMR of **7c**.

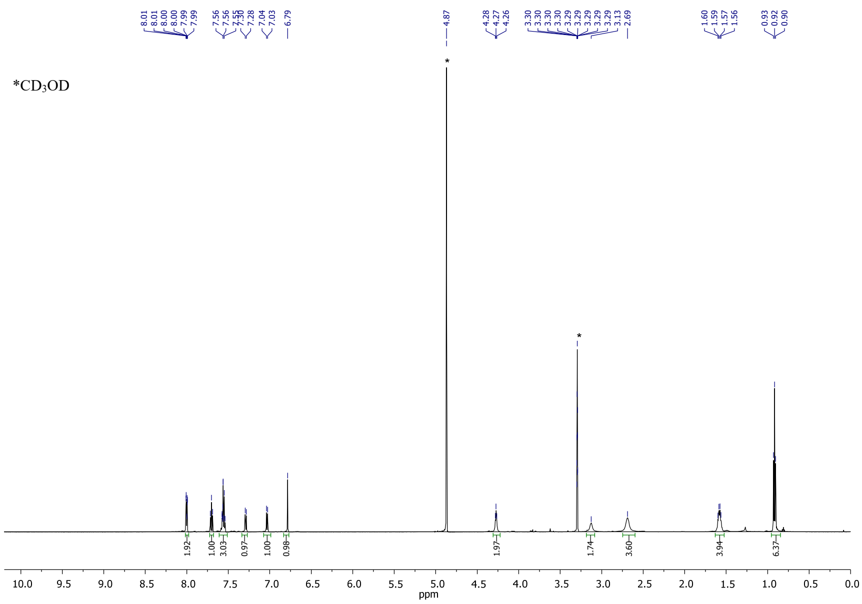

Supporting Information Figure S47. ^{1}H NMR of **8a**.

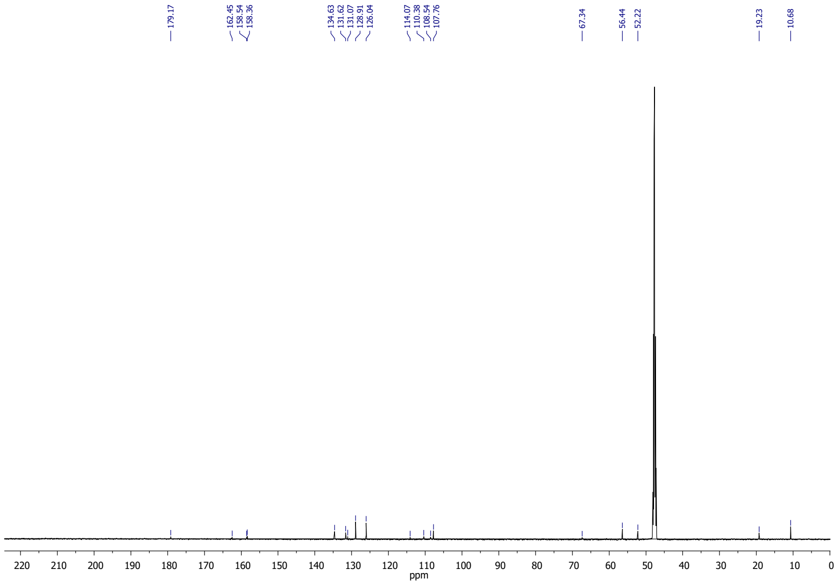

Supporting Information Figure S48. ^{13}C NMR of **8a**.

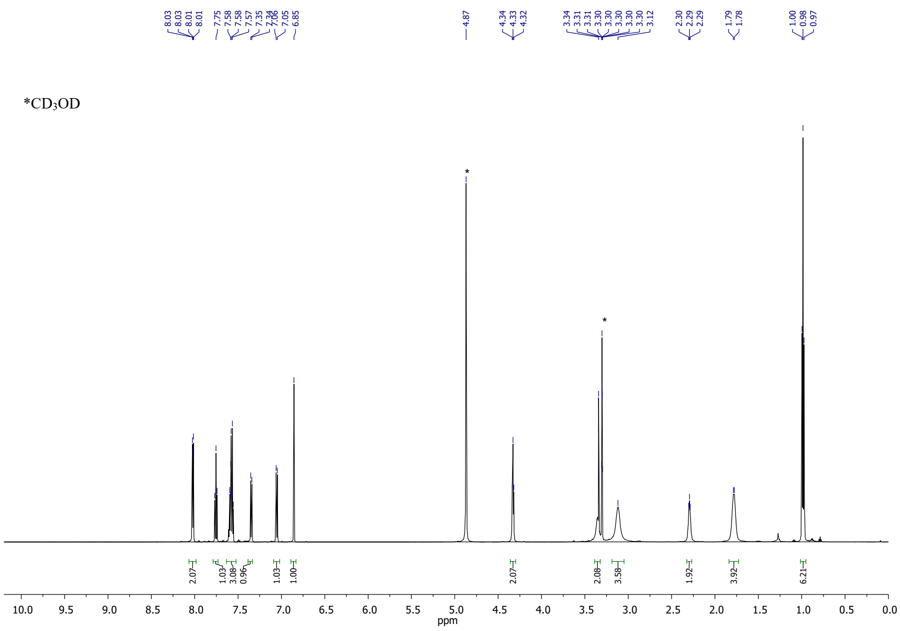

Supporting Information Figure S49. ^{1}H NMR of **8b**.

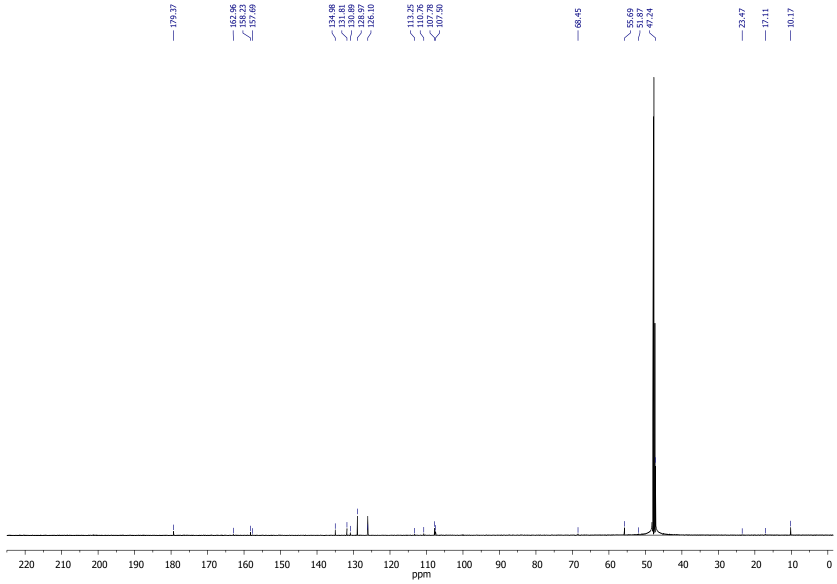

Supporting Information Figure S50. ^{13}C NMR of **8b**.

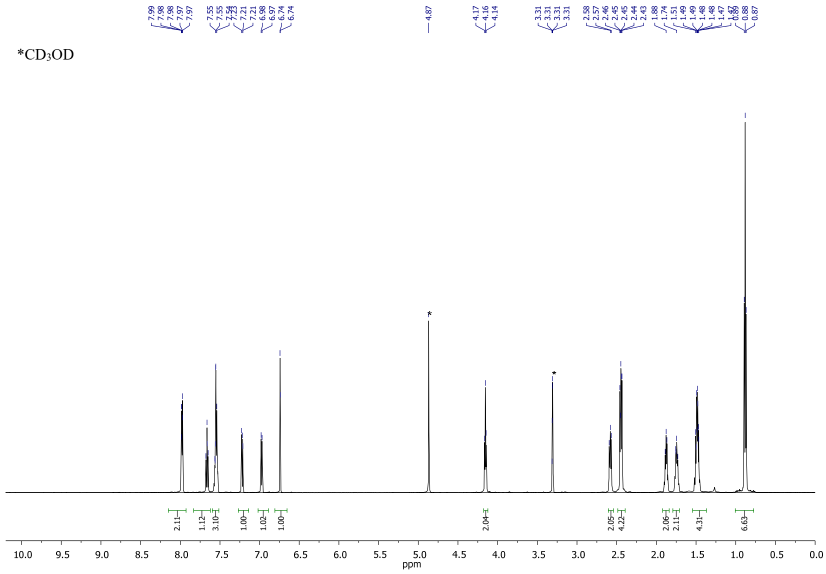

Supporting Information Figure S51. ^{1}H NMR of **8c**.

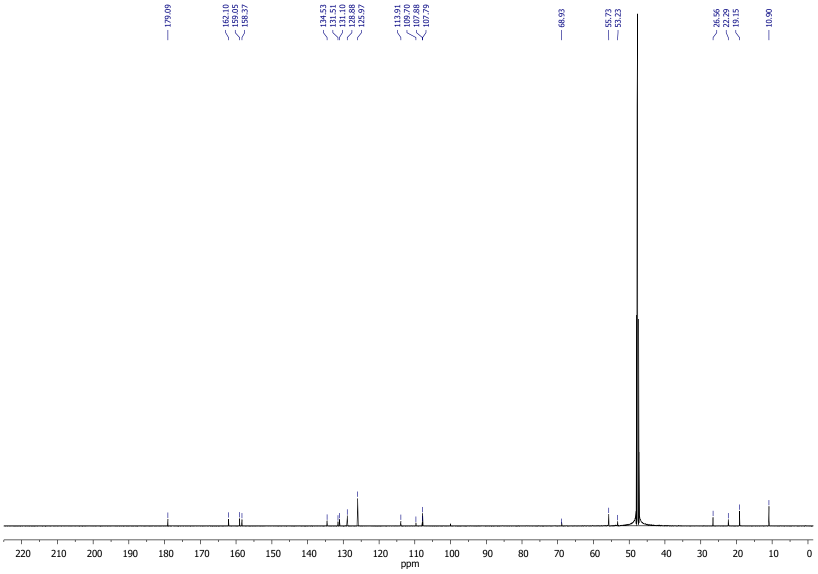

Supporting Information Figure S52. ^{13}C NMR of **8c**.

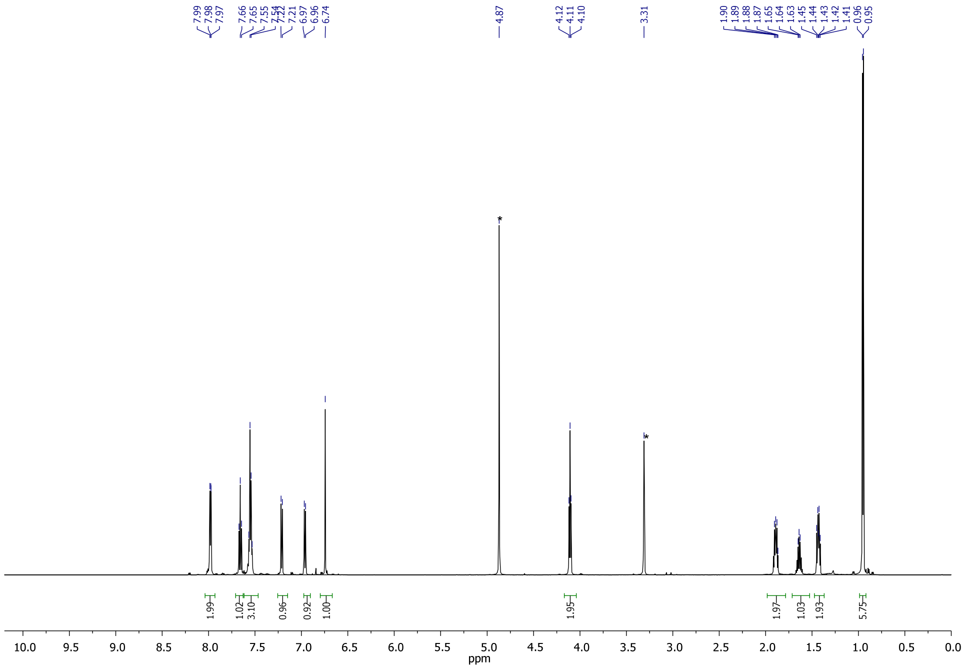

Supporting Information Figure S53. ^{1}H NMR of **9b**.

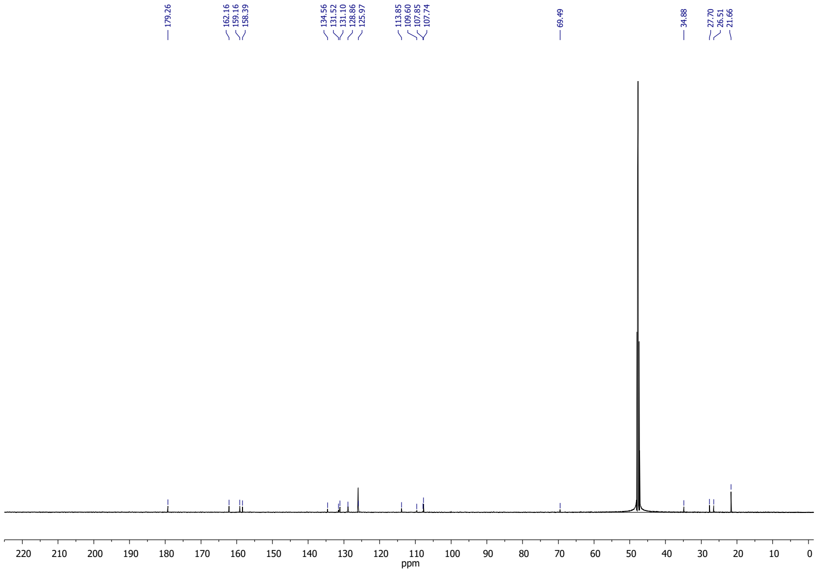

Supporting Information Figure S54. ^{13}C NMR of **9b**.

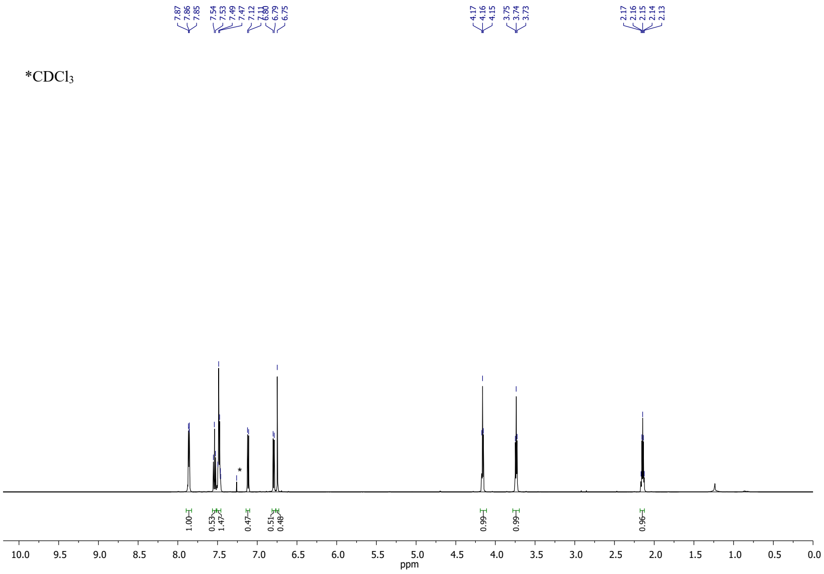

Supporting Information Figure S55. ^{1}H NMR of **12**.

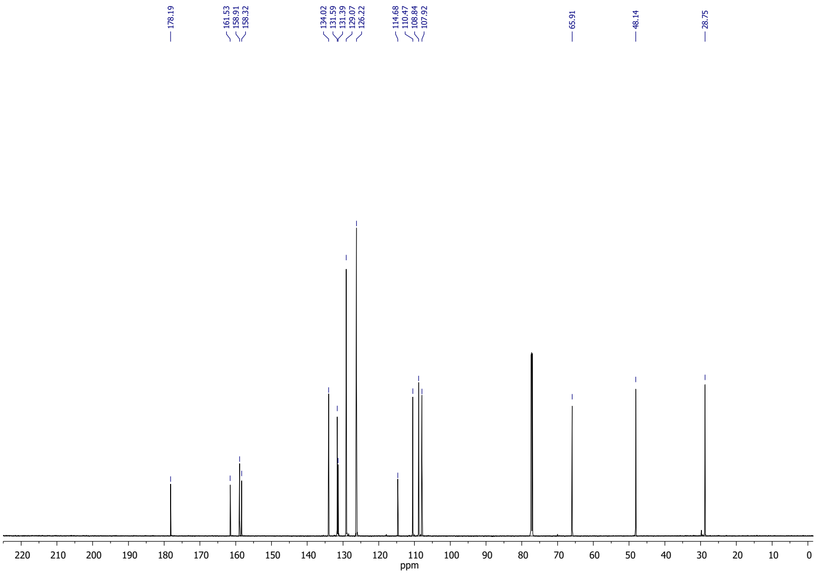

Supporting Information Figure S56. ^{13}C NMR of **12**.

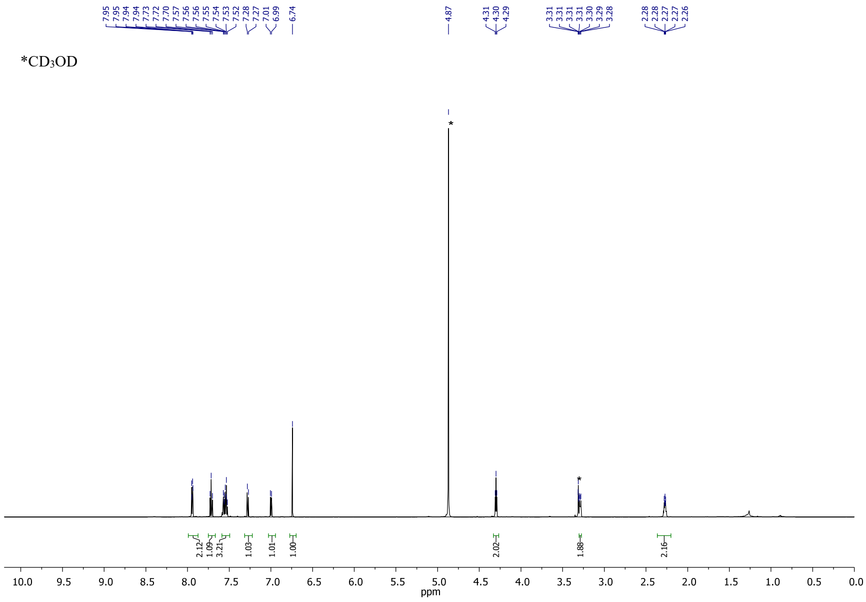

Supporting Information Figure S57. ^{1}H NMR of **10b**.

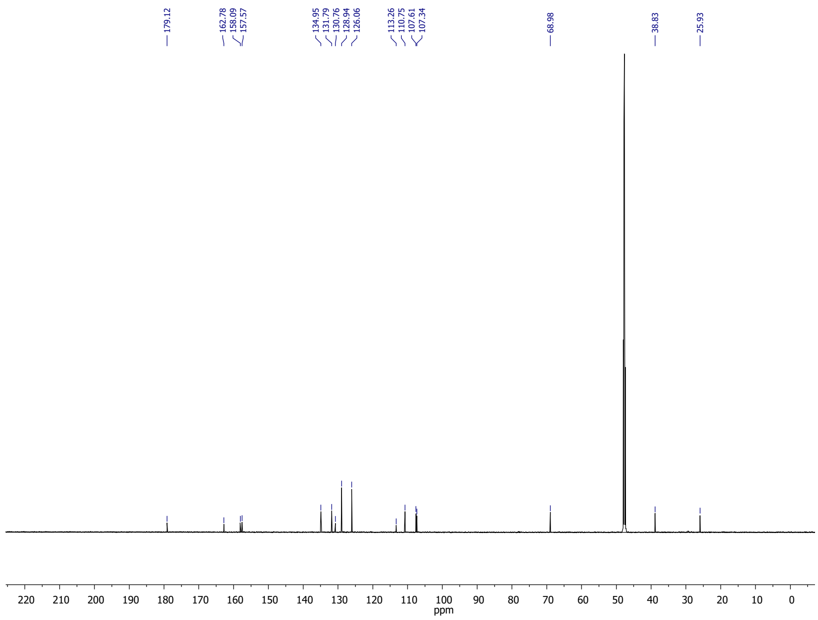

Supporting Information Figure S58. ^{13}C NMR of **10b**.

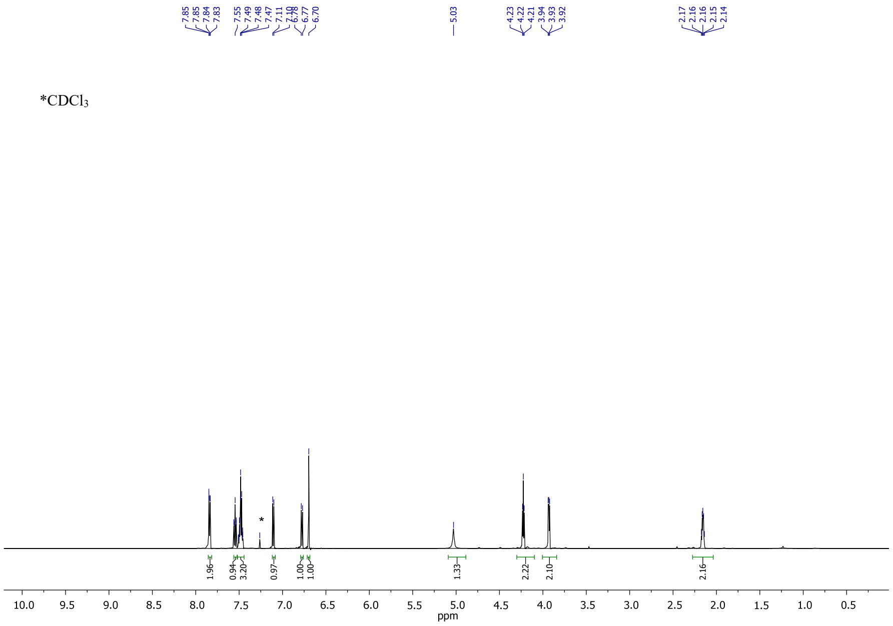

Supporting Information Figure S59. ^{1}H NMR of **11b**.

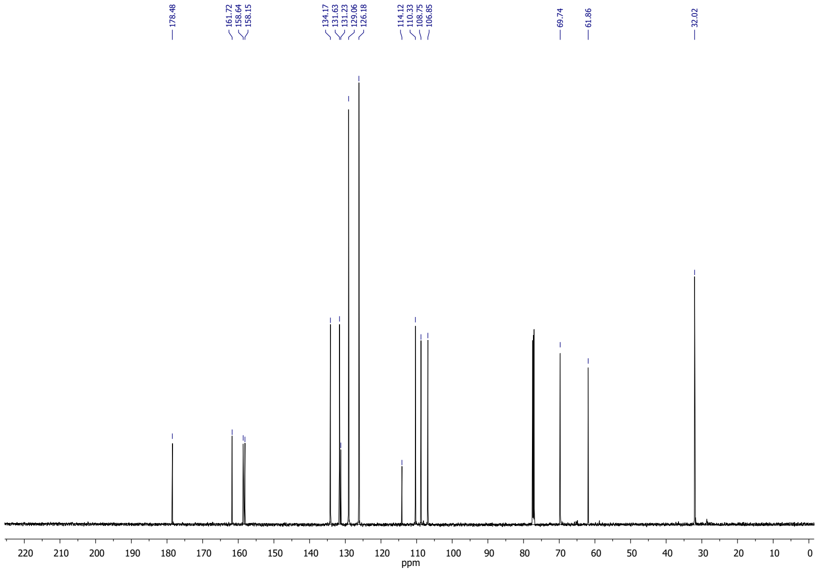

Supporting Information Figure S60. ^{13}C NMR of **11b**.

HRMS (DART) *m/z*: calcd. for $C_{17}H_{14}BrO_3$ $[M+H]^+$: 345.0121, found 345.0132.

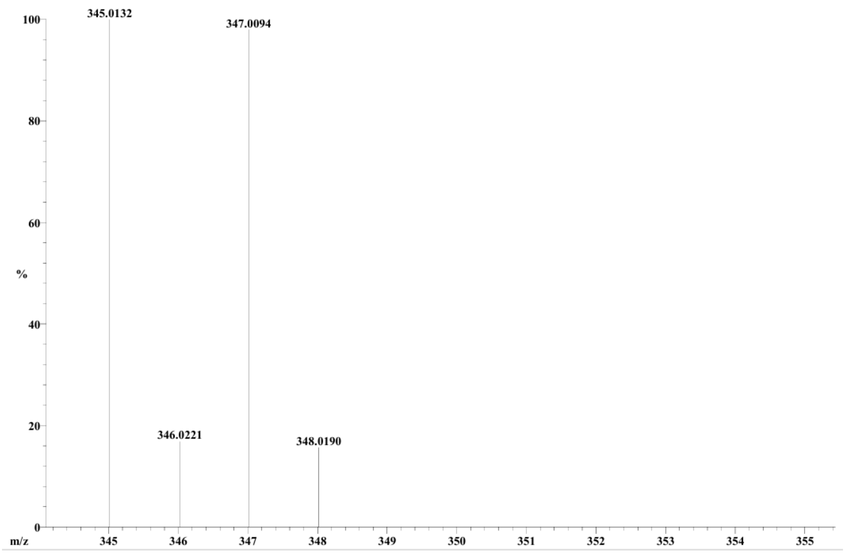

Supporting Information Figure S61. Mass spectrum of **2a.**

HRMS (DART) *m/z*: calcd. for $C_{18}H_{16}BrO_3$ $[M+H]^+$: 359.0277, found 359.0203.

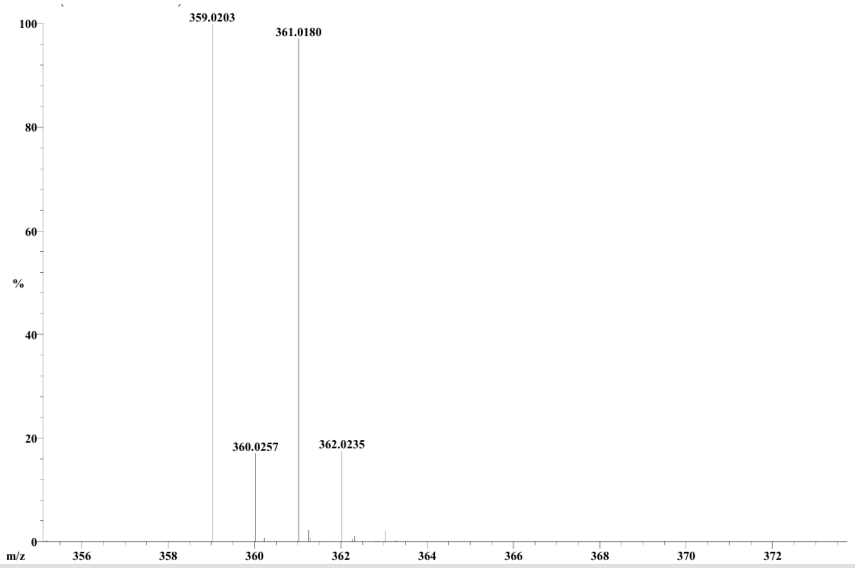

Supporting Information Figure S62. Mass spectrum of **2b.**

HRMS (DART) *m/z*: calcd. for $C_{19}H_{18}BrO_3$ $[M+H]^+$: 373.0434, found 373.0399.

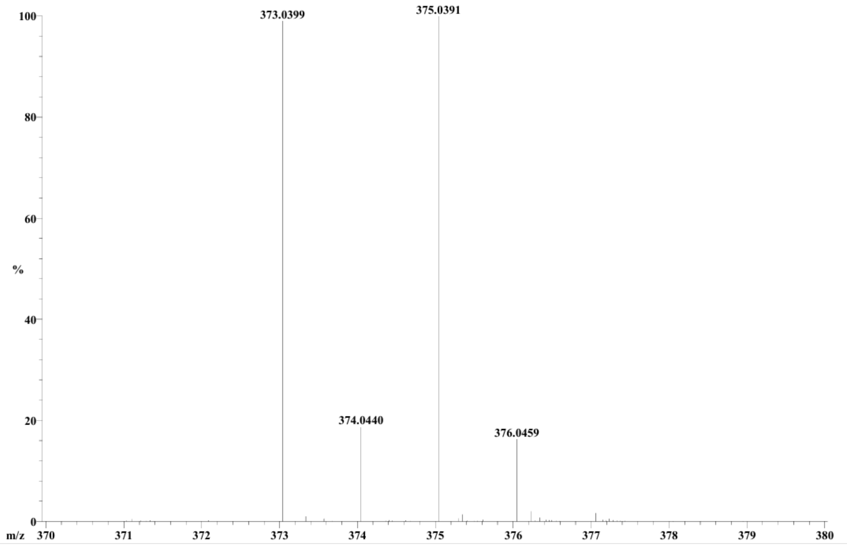

Supporting Information Figure S63. Mass spectrum of **2c.**

HRMS (DART) *m/z*: calcd for $C_{21}H_{22}NO_3$ $[M+H]^+$: 336.1594, found 336.1614.

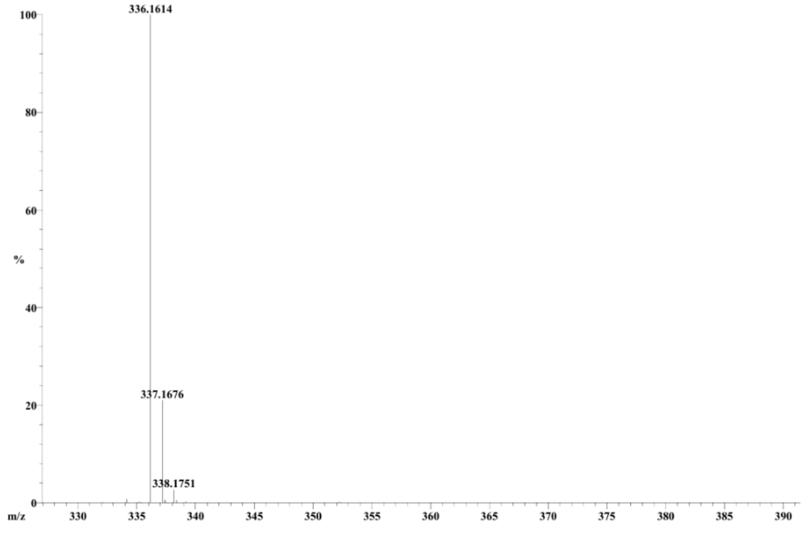

Supporting Information Figure S64. Mass spectrum of **3a.**

HRMS (DART) *m/z*: calcd. for $C_{22}H_{24}NO_3$ $[M+H]^+$: 350.1751, found 350.1724.

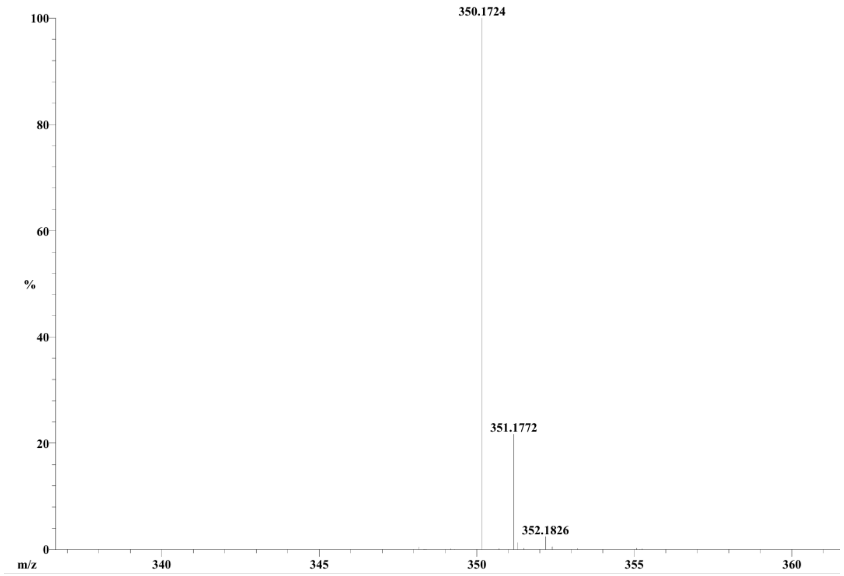

Supporting Information Figure S65. Mass spectrum of **3b.**

HRMS (DART) *m/z*: calcd. for $C_{23}H_{26}NO_3$ $[M+H]^+$: 364.1907, found 364.1908.

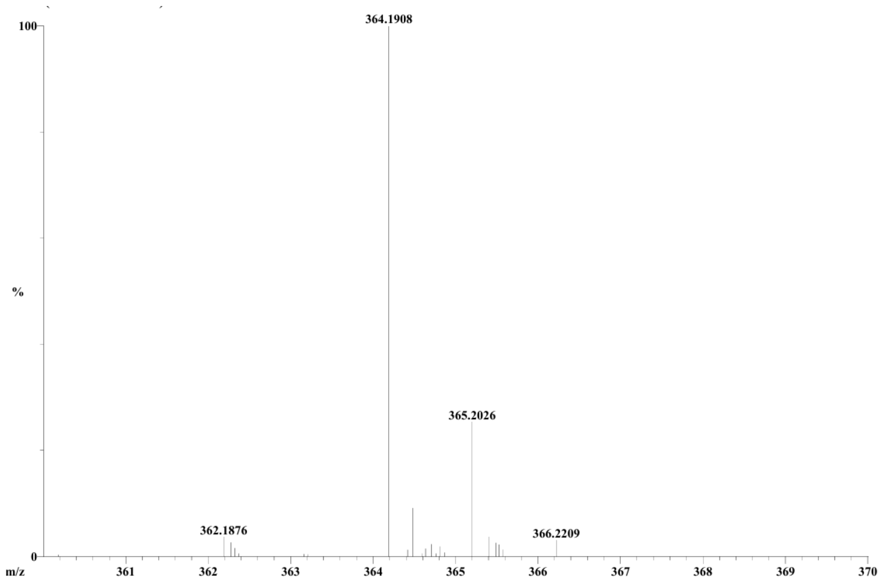

Supporting Information Figure S66. Mass spectrum of **3c.**

HRMS (DART) *m/z*: calcd. for $C_{22}H_{24}NO_3$ $[M+H]^+$: 350.1751, found 350.1752.

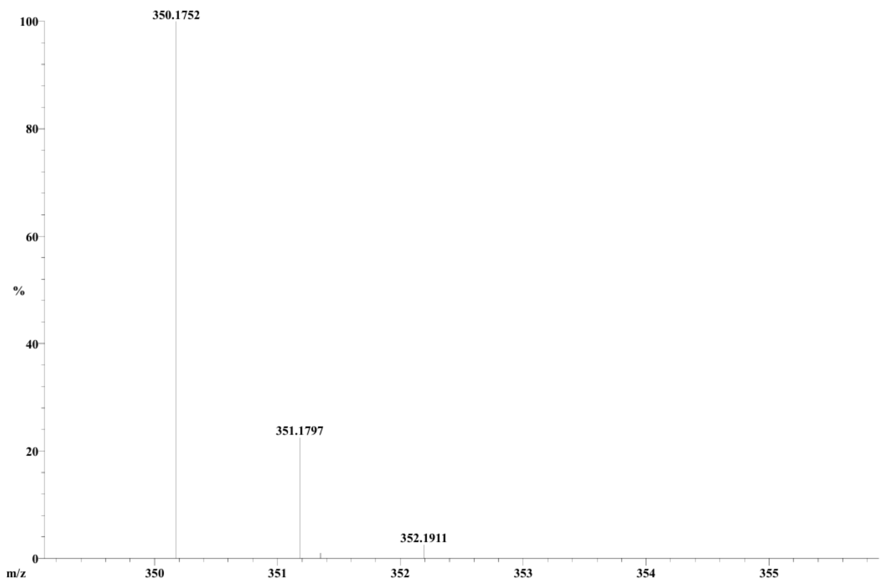

Supporting Information Figure S67. Mass spectrum of **4a.**

HRMS (DART) *m/z*: calcd. for $C_{23}H_{26}NO_3$ $[M+H]^+$: 364.1907, found 364.1860.

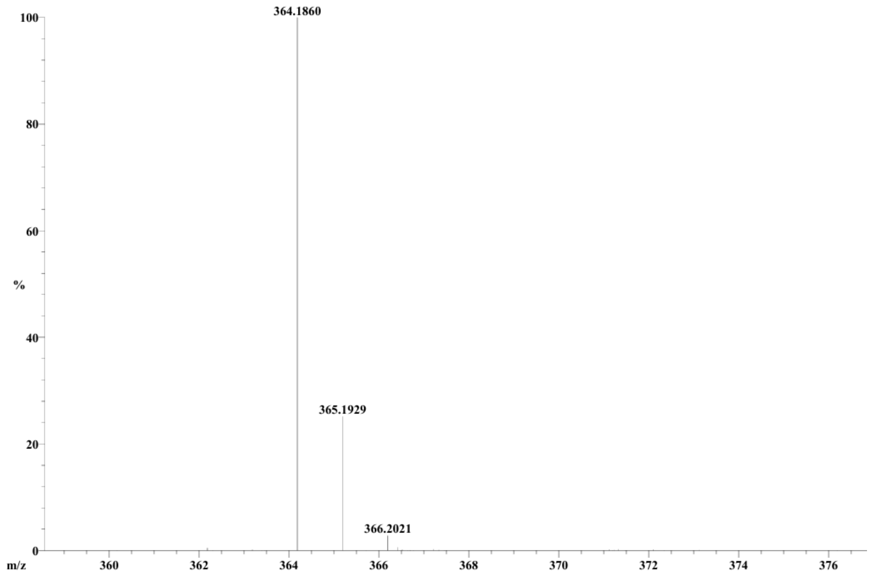

Supporting Information Figure S68. Mass spectrum of **4b.**

HRMS (DART) *m/z*: calcd. for $C_{24}H_{28}NO_3$ $[M+H]^+$: 378.2064, found 378.2038.

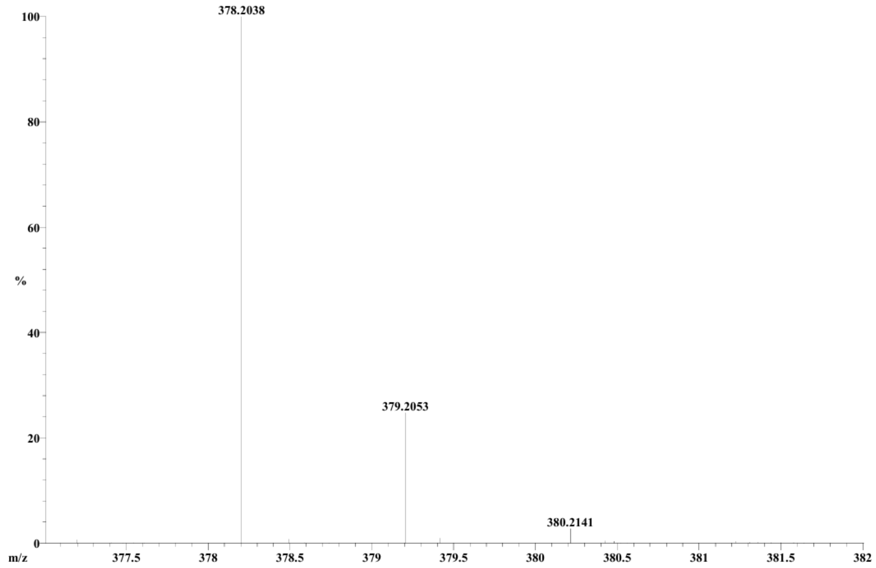

Supporting Information Figure S69. Mass spectrum of **4c.**

HRMS (ESI) *m/z*: calcd. for $C_{21}H_{22}NO_4$ $[M+H]^+$: 352.1543, found 352.1553.

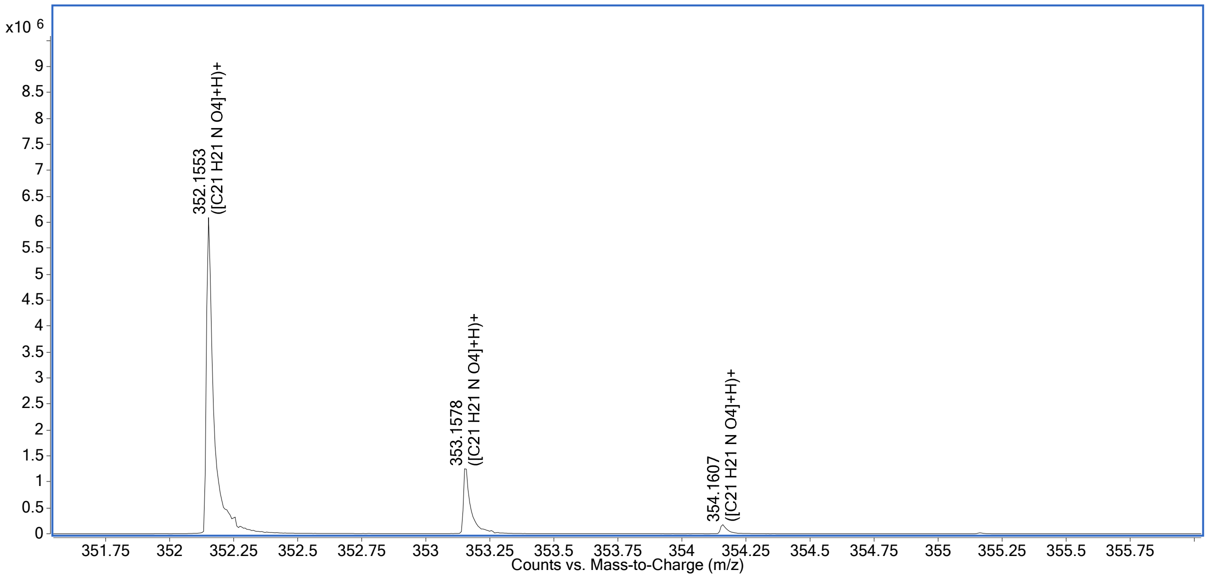

Supporting Information Figure S70. Mass spectrum of **5a.**

HRMS (DART) *m/z*: calcd. for $C_{22}H_{24}NO_4$ $[M+H]^+$: 366.1700, found 366.1674.

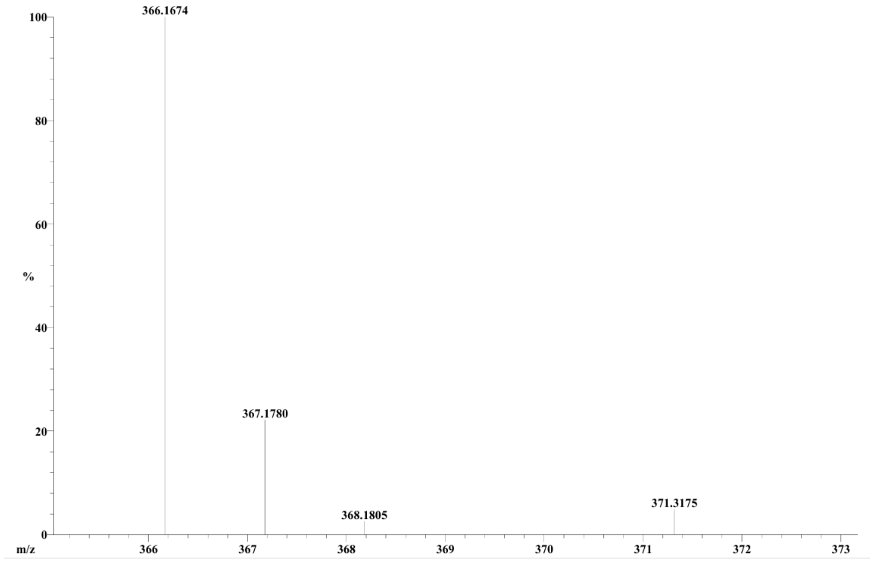

Supporting Information Figure S71. Mass spectrum of **5b.**

HRMS (DART) *m/z*: calcd. for $C_{23}H_{26}NO_4$ $[M+H]^+$: 380.1856, found 380.1804.

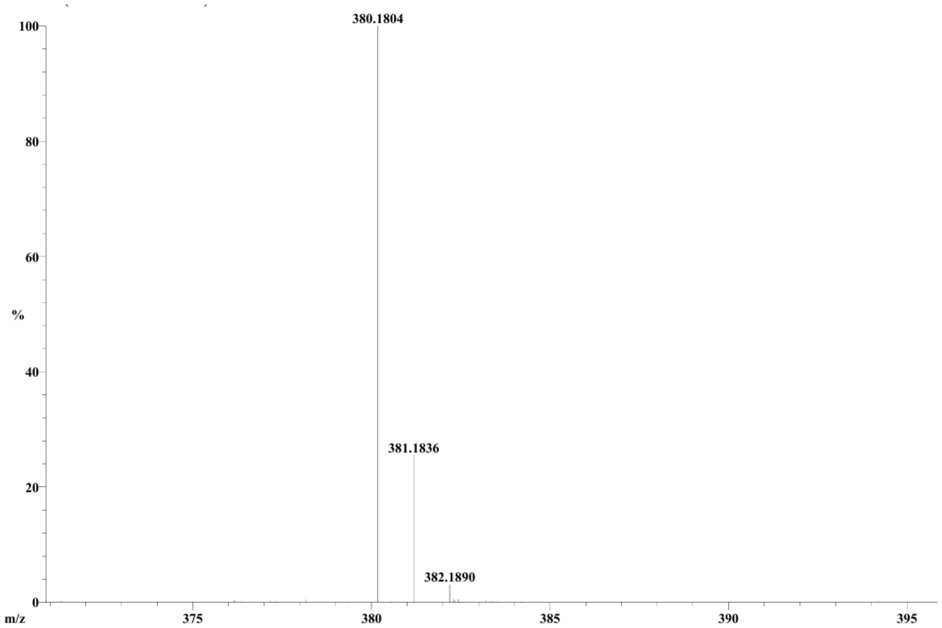

Supporting Information Figure S72. Mass spectrum of **5c.**

HRMS (DART) *m/z*: calcd. for $C_{23}H_{27}N_2O_3$ $[M+H]^+$: 379.2016, found 379.2033.

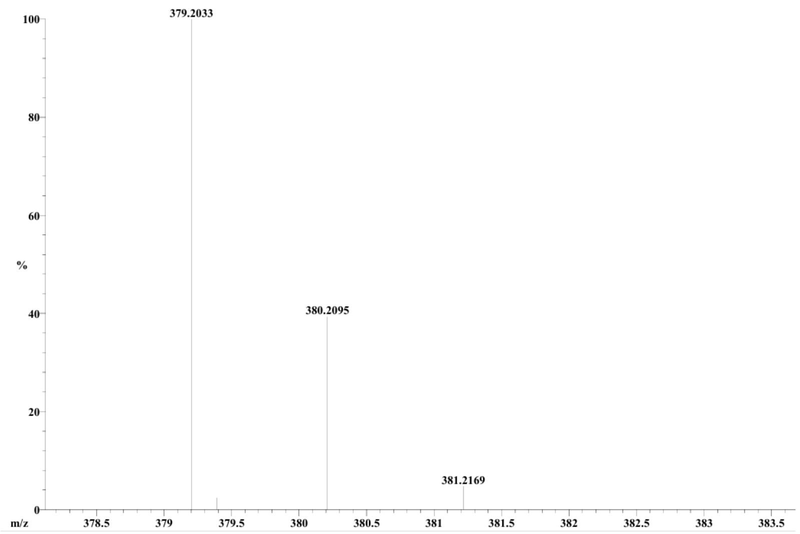

Supporting Information Figure S73. Mass spectrum of **6b.**

HRMS (DART) *m/z*: calcd. for $C_{24}H_{29}N_2O_3$ $[M+H]^+$: 393.2173, found 393.2134.

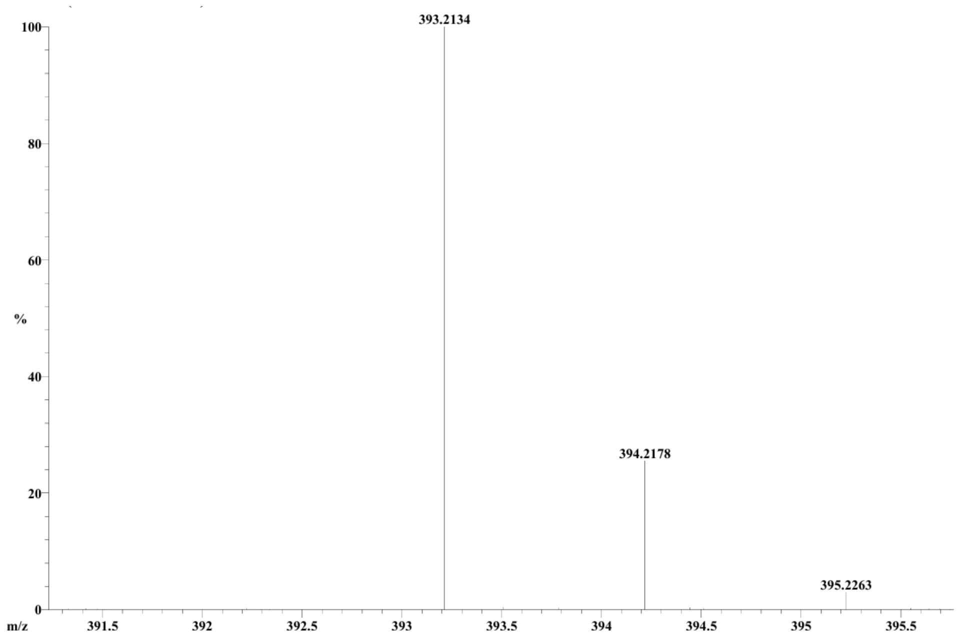

Supporting Information Figure S74. Mass spectrum of **6c.**

HRMS (DART) *m/z*: calcd. for $C_{21}H_{24}NO_3$ $[M+H]^+$: 338.1751, found 338.1752.

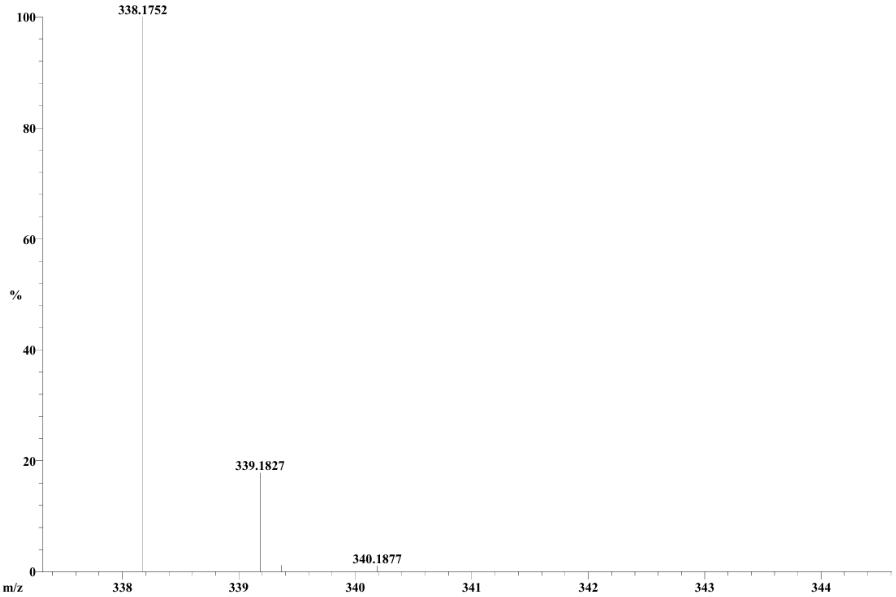

Supporting Information Figure S75. Mass spectrum of **7a.**

HRMS (DART) *m/z*: calcd. for $C_{22}H_{26}NO_3$ $[M+H]^+$: 352.1907, found 352.1862.

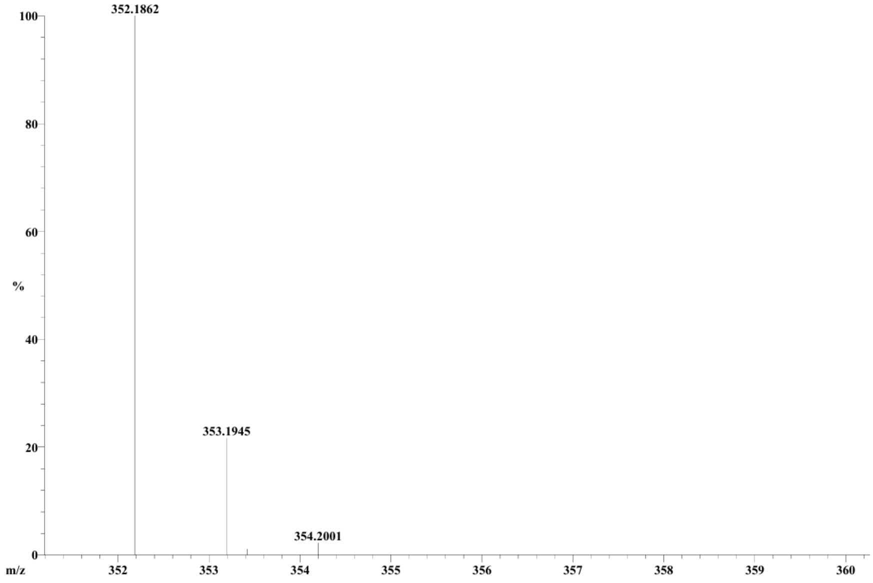

Supporting Information Figure S76. Mass spectrum of **7b.**

HRMS (DART) *m/z*: calcd. for $C_{23}H_{28}NO_3$ $[M+H]^+$: 366.2064, found 366.2096.

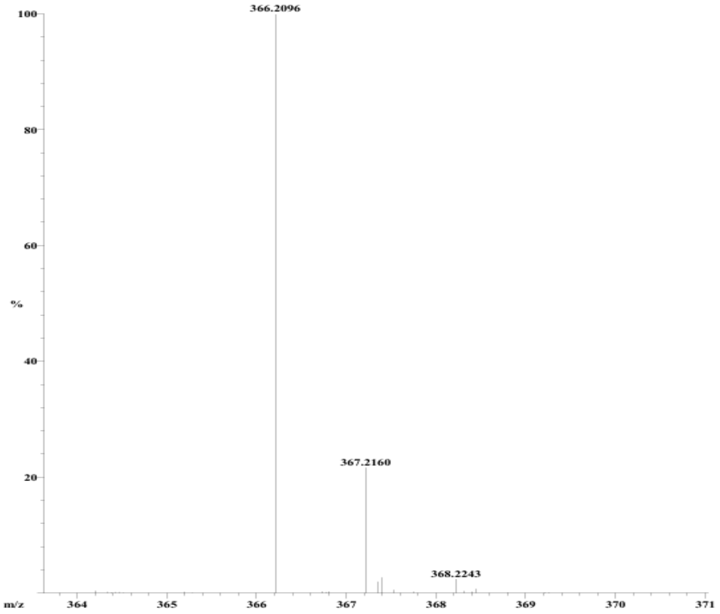

Supporting Information Figure S77. Mass spectrum of **7c.**

HRMS (DART) *m/z*: calcd. for $C_{23}H_{28}NO_3$ $[M+H]^+$: 366.2064, found 366.2116.

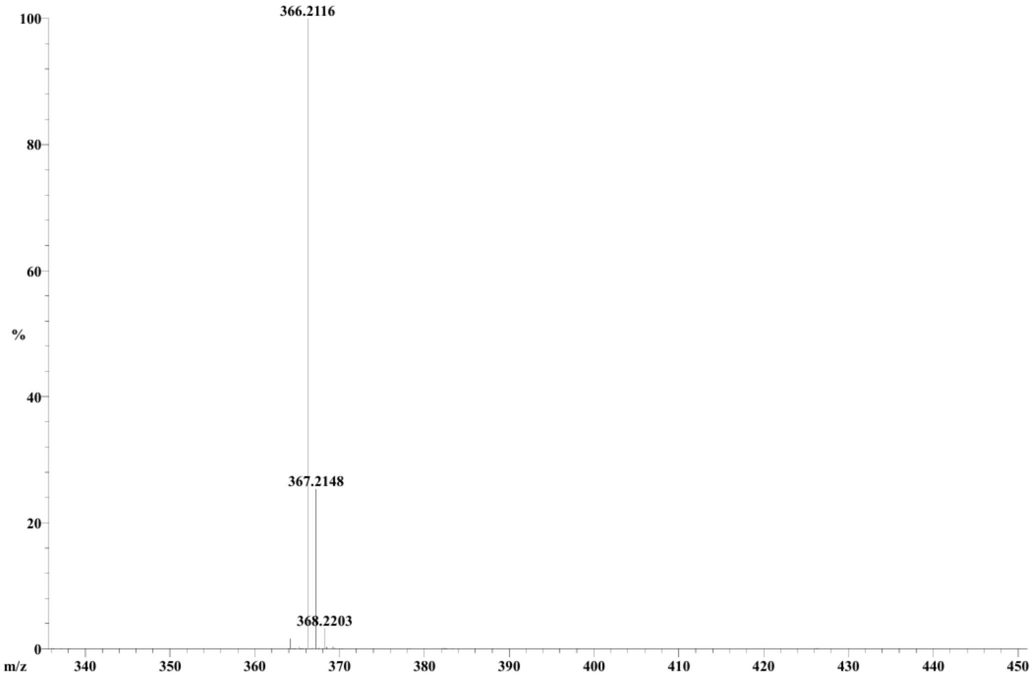

Supporting Information Figure S78. Mass spectrum of **8a.**

HRMS (DART) *m/z*: calcd. for $C_{24}H_{30}NO_3$ $[M+H]^+$: 380.2220, found 380.2213.

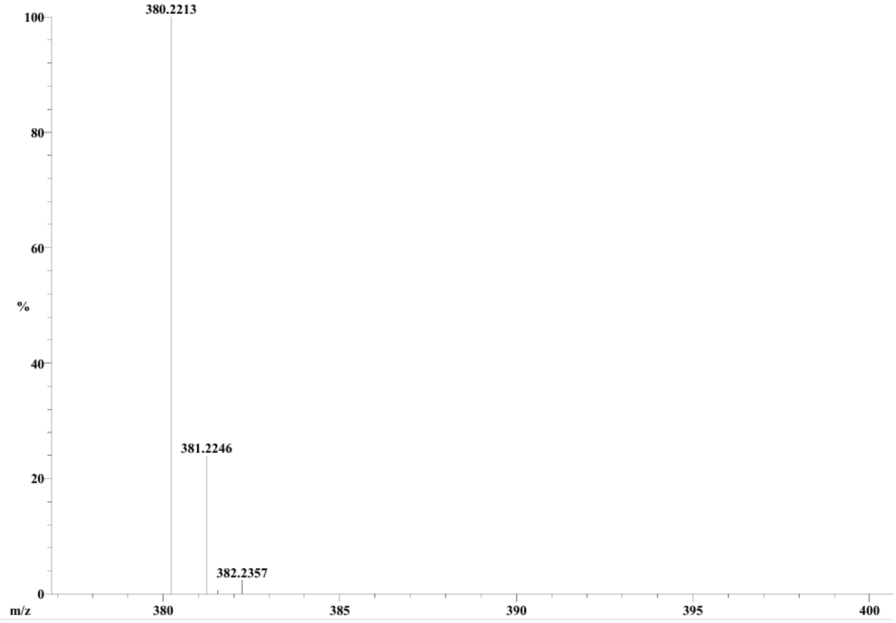

Supporting Information Figure S79. Mass spectrum of **8b.**

HRMS (DART) *m/z*: calcd. for $C_{25}H_{32}NO_3$ $[M+H]^+$: 394.2377, found 394.2305.

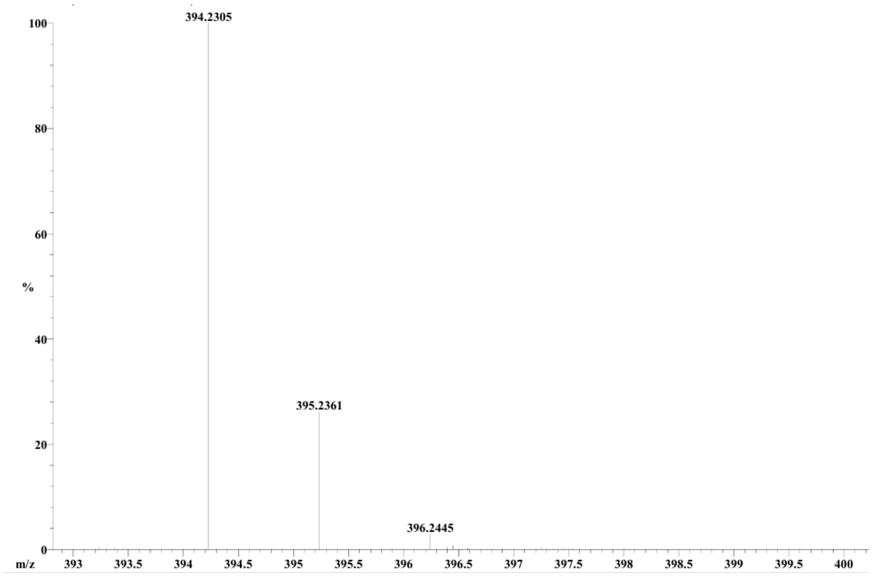

Supporting Information Figure S80. Mass spectrum of **8c.**

HRMS (ESI) *m/z*: calcd. for $C_{21}H_{23}O_3$ $[M+H]^+$: 323.1642, found 323.1643.

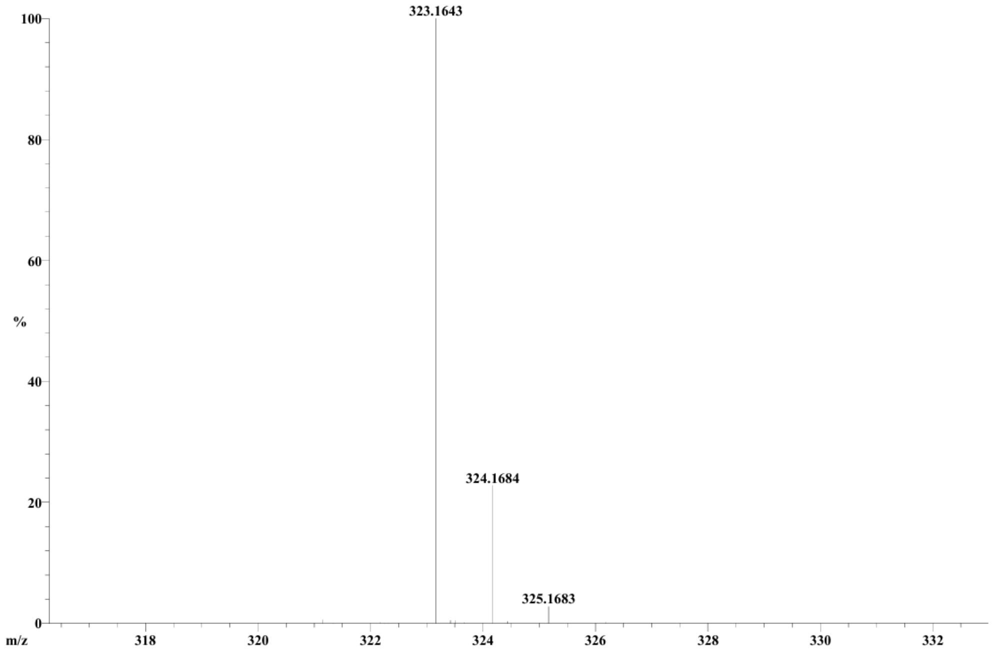

Supporting Information Figure S81. Mass spectrum of **9b.**

HRMS (ESI) *m/z*: calcd. for $C_{18}H_{15}N_3O_3$ $[M+H]^+$: 322.1186, found 322.1216.

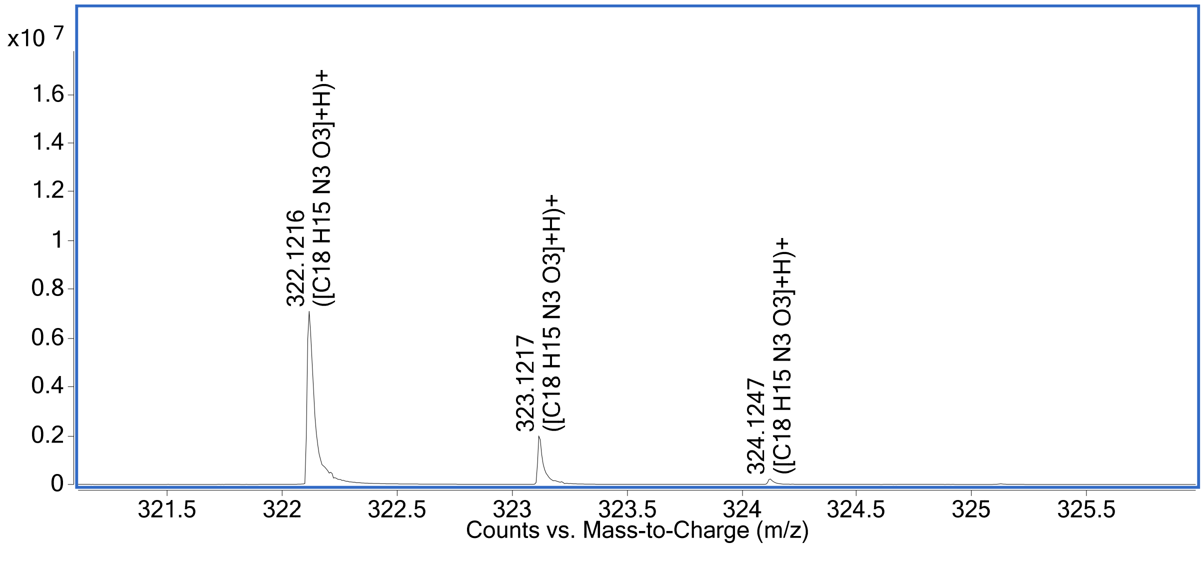

Supporting Information Figure S82. Mass spectrum of **12.**

HRMS (ESI) *m/z*: calcd. for $C_{18}H_{17}NO_3$ $[M+H]^+$: 296.1281, found 296.1301.

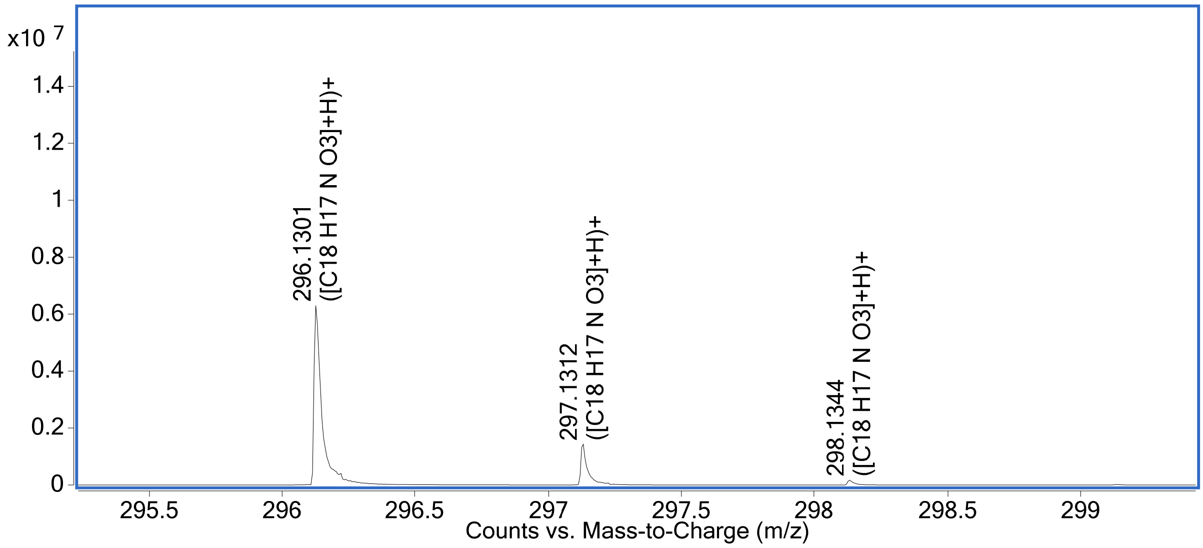

Supporting Information Figure S83. Mass spectrum of **10b.**

HRMS (ESI) *m/z*: calcd. for $C_{18}H_{16}O_4$ $[M+H]^+$: 297.1121, found 297.1133.

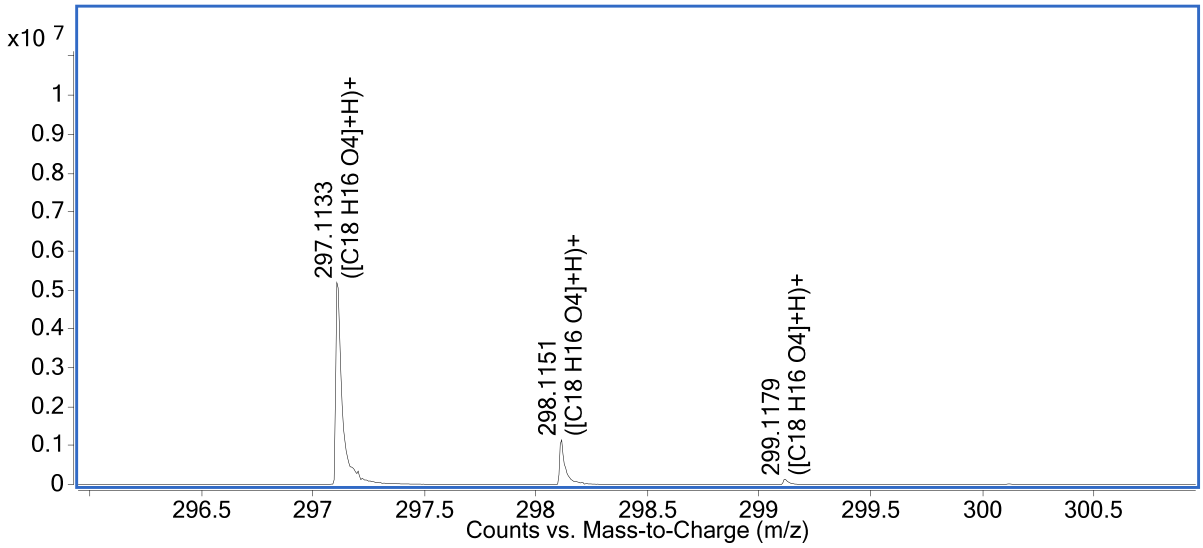

Supporting Information Figure S84. Mass spectrum of **11b.**

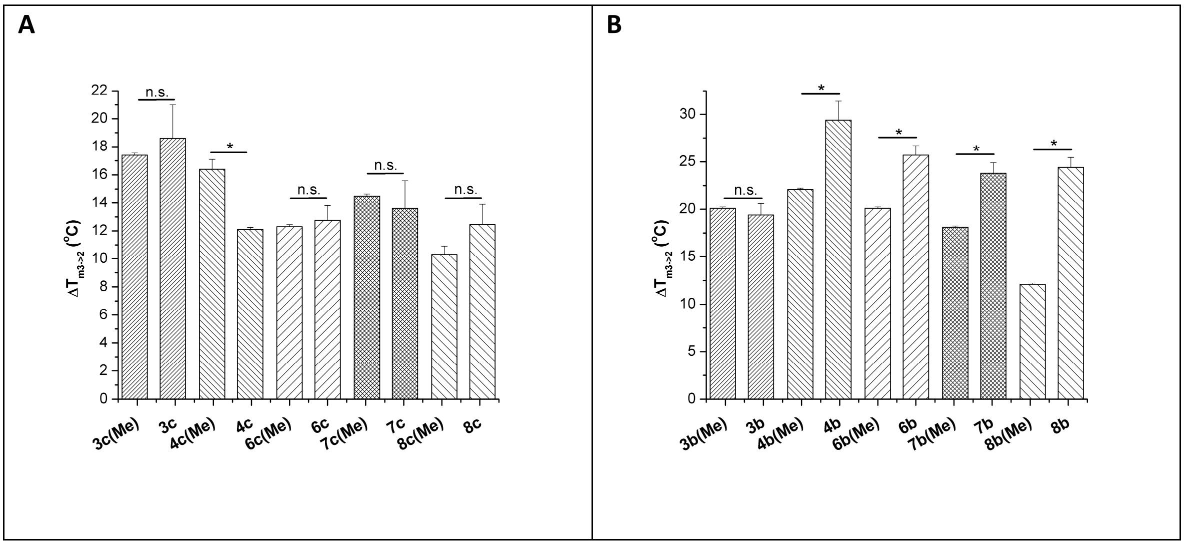

Supporting Information Figure S85. Changes in UV melting temperatures ($\Delta T_{m3\to2}$) of poly(dA)•2poly(dT) at 260 nm in the presence and absence of a 10 µM ligand. A) Comparison of ligands with a butylene (C4) linker shows that most of the 5-substituted flavones with a butylene linker stabilize triplex DNA equally well compared to the corresponding 5-substituted 3,3′,4′,7-tetramethoxyflavonoids. Only 5-substituted 3,3′,4′,7-tetramethoxyflavonoid with a piperidine group [compound **4c(Me)**] stabilizes triplex DNA more significantly than the corresponding 5-substituted flavones (compound **4c**). B) Comparison of ligands with a propylene (C3) linker shows that most of the 5-substituted flavones with a propylene linker stabilize triplex DNA much more strongly than the corresponding 5-substituted 3,3′,4′,7-tetramethoxyflavonoids. Only compound [**3b(Me)**] has a similar stabilization effect to compound **3b**. Compound codes are the same as the ones used in the main text. Compounds with (Me) represent 5-substituted 3,3′,4′,7-tetramethoxyflavonoids. [DNA] = 15 µM/base triplet. Buffer: 10 mM sodium cacodylate, 150 mM KCl, pH 7.0. A two-tailed unpaired t-test was performed using Excel. Statistical differences between 5-substituted 3,3′,4′,7-tetramethoxyflavonoids and the corresponding 5-substituted flavones are represented by the symbols * ($P < 0.05$) and n.s. (not significant, $P > 0.05$).
